# A Summary of Two Decades of QTL and Candidate Genes That Control Seed Tocopherol Contents in Maize (*Zea mays* L.)

**DOI:** 10.3390/genes15040472

**Published:** 2024-04-09

**Authors:** My Abdelmajid Kassem, Dounya Knizia, Khalid Meksem

**Affiliations:** 1 Plant Genomics and Biotechnology Laboratory, Department of Biological and Forensic Sciences, Fayetteville State University, Fayetteville, NC 28301, USA; 2 School of Agricultural Sciences, Southern Illinois University, Carbondale, IL 62901, USA; dounya.knizia@siu.edu (D.K.); meksem@siu.edu (K.M.)

**Keywords:** *Zea mays*, QTL, Tocopherols, α–tocopherol, β–tocopherol, γ–tocopherol, δ–tocopherol

## Abstract

Tocopherols are secondary metabolites synthesized through the shikimate biosynthetic pathway in the plastids of most plants. It is well known that α–Tocopherol (vitamin E) has many health benefits for humans and animals; therefore, it is highly used in human and animal diets. Tocopherols vary considerably in most crop (and plant) species and within cultivars of the same species depending on environmental and growth conditions; tocopherol content is a polygenic, complex traits, and its inheritance is poorly understood. The objective of this review paper was to summarize all identified quantitative trait loci (QTL) that control seed tocopherols and related contents identified in maize (*Zea mays*) during the past two decades (2002–2022). Candidate genes identified within these QTL regions are also discussed. The QTL described here, and candidate genes identified within these genomic regions could be used in breeding programs to develop maize cultivars with high, beneficial levels of seed tocopherol contents.

## 1. Introduction

Tocopherols play a crucial role in plant stress resistance and are considered an essential nutrient for animals, due to their vitamin E activity. In plants, tocopherols exist in four forms: α–tocopherol (α–Toc), β–tocopherol (β–Toc), γ–tocopherol (γ–Toc), and δ–tocopherol (δ–Toc). These forms differ from each other in terms of biological activity and molecular structure. The α–Toc form, also known as vitamin E, is the most bioactive tocopherol molecule [1,2]. These forms are synthesized through the shikimate biosynthetic pathway in the plastids of most plants, including *Brassica napus*, maize (*Z. mays*), sunflower (*Helianthus annuus*) [3,4], and other plant species such as pecans, Brazil nuts, and peanuts [5].

In most plants, stress tolerance is achieved by an increase in vitamin E (α–Toc) content, which deactivates reactive oxygen species (ROS) and prevents lipid peroxidation [6,7,8]. Vitamin E (α–Toc) is also involved in membrane stability, cyclic electron flow during photosynthesis, and signal transduction [9,10,11], while γ–Toc has been shown to be involved in protecting unsaturated fatty acids and improving seed-desiccation tolerance [12]. Also, both α–Toc and γ–Toc have been shown to reduce salt and sorbitol stresses [11,13]. A study provided ample evidence that tocopherols help plants cope with biotic stresses such as drought, cold, heat, and salt stresses as well as biotic stresses such as fungal and bacterial infections [8]. The role of tocopherols in resistance to biotic stresses is more likely achieved by the regulation of fatty acid biosynthesis [8].

Several studies reported that tocopherols, especially vitamin E (α–Toc), have many health benefits for humans and animals [14,15,16,17,18,19]; however, other studies reported that they may cause harm to humans [20,21]. For example, it was reported that vitamin E reduces the risks of vascular diseases, atherosclerosis by low density lipoprotein (LDL) oxidation, and certain types of cancer [18]; therefore, it is widely used in animal and human diets. Furthermore, other studies reported ample evidence that vitamin E plays a major role in reducing inflammation, obesity, hyperglycemia, and high cholesterol (dyslipidemia) in animals [14,15,16,19] and humans [19,22,23];

It is well established that environmental conditions, growth conditions, plant genotype, and genotype by environment (G × E) interactions influence seed tocopherol contents in plants [24,25,26]. For example, a population of elite inbred maize strains (*n* = 87) was grown in China over a period of two years (2004–2005) and researchers quantified seed carotenoids, starch, protein, tocopherol (α–tocopherol, γ–tocopherol, δ–tocopherol, total tocopherol, and (α/γ)–tocopherol ratio) and many other agronomic traits [24]. The mean levels of seed α–Toc, γ–Toc, δ–Toc, and T–Toc contents were found to be 23.98 μg g^−1^, 32.90 μg g^−1^, 2.189 μg g^−1^, and 59.55 μg g^−1^, respectively [24]. In a separate study using a population of inbred maize strains, the seed α–Toc and γ–Toc content ranges were 9.1–64.6 g g^−1^ and 13.6–128.7 g g^−1^, respectively [27]. Yet another study conducted on soybean found the ranges for seed α–Toc, β–Toc, γ–Toc, and δ–Toc contents to be 114.1–139.1 mg kg^−1^ oil, 37.9–51.7 mg kg^−1^ oil, 939.8–1080.4 mg kg^−1^ oil, and 307.8–397.3 mg kg^−1^ oil, respectively [26].

Therefore, given the fact that tocopherol content is a multifactorial complex polygenic trait, several studies mapped QTL that control the seed contents of α–Toc, β–Toc, γ–Toc, δ–Toc, and T–Toc in maize [24,28,29], rapeseed (*B. napus*) [30,31,32], sunflower [33,34], and other plants [35].

The objective of this review paper was to summarize all identified quantitative trait loci (QTL) and candidate genes identified in maize (*Z. mays* L.).

## 2. Tocopherols and Tocotrienols Biosynthetic Pathway

**Figure 1:**
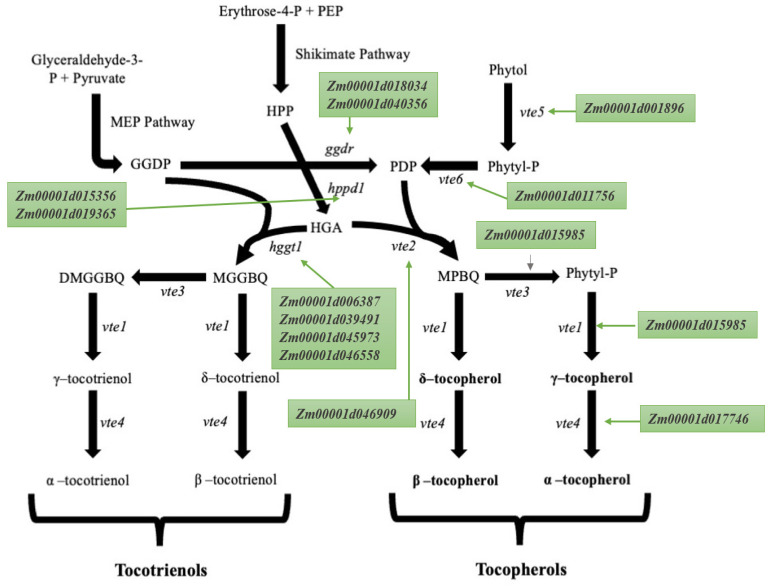
Tocopherol (α–tocopherol, δ–tocopherol, γ–tocopherol, and β–tocopherol) biosynthetic pathway through the shikimic acid and MEP (Methylerythritol Phosphate) biosynthetic pathways, showing the candidate genes in *Z. mays*. Abbreviations: Phytyl-P—Phytyl Monophosphate; PEP—Phosphoenolpyruvate; PDP—Phytyldiphosphate; HPP—p-Hydroxyphenylpyruvate; GGDP—Geranylgeranyl Diphosphate; HGA—Homogentisic acid; MPBQ—2-Methyl-6-Phytyl-1,4-Benzoquinol; DMPBQ—2,3-Dimethyl-5-Phytylbenzoquinol; MGGBQ—2-Methyl-6-Geranylgeranyl-1,4-Benzoquinol; DMGGBQ—2,3-Dimethyl-5-Geranylgeranyl-1,4-Benzoquinol; *hggt1*—Homogentisate Geranylgeranyltransferase; *ggdr*—Geranylgeranyl Diphosphate Reductase; *hppd1*—4-Hydroxyphenylpyruvate Dioxygenase; *vte1*—Tocopherol Cyclase; *vte2*—Homogentisate Phytyltransferase; *vte3*—MPBQ/MGGBQ Methyltransferase; *vte4*—γ-Tocopherol-Methyltransferase; *vte5*—Phytol Kinase; and *vte6*—Phytol Phosphate Kinase. Adopted from Baseggio et al. (2019) [36].

As mentioned earlier, tocopherols (α–tocopherol, β–tocopherol, γ–tocopherol, and δ–tocopherol) are biosynthesized through the shikimate and methylerythritol biosynthetic pathways in the plastids of most plants, including maize [36] (Figure 1). Here, we present a brief overview of the tocopherol biosynthetic pathway in maize (Figure 1). The precursor molecules are phosphoenolpyruvate (PEP) derived from glycolysis and erythrose 4-phosphate (E4P) derived from the pentose phosphate pathway. The condensation of PEP and E4P is catalyzed by 1-deoxy-D-xylulose-5-phosphate synthase (DXS), leading to the formation of 1-deoxy-D-xylulose 5-phosphate (DXP) and then p-Hydroxyphenylpyruvate (HPP) through the shikimate pathway. GGDP (Geranylgeranyl diphosphate), an intermediate in the MEP pathway, can be converted into different forms, including PDP (Phytyldiphosphate) and Phytyl-P (Phytyl monophosphate). PDP and Phytyl-P act as potential donors of the phytyl tail during tocopherol synthesis. HPP (p-Hydroxyphenylpyruvate) acts as a substrate for the enzyme hppd1 (4-hydroxyphenylpyruvate dioxygenase), generating HGA (Homogentisic Acid). HGA is combined with GGDP or PDP to form precursor molecules such as 2,3-dimethyl-6-phytyl-1,4-benzoquinol (DMPBQ) and 2-methyl-6-geranylgeranyl-1,4-benzoquinol (DMGGBQ) (Figure 1). A series of enzymatic steps involving enzymes like CMS (4-diphosphocytidyl-2-C-methyl-D-erythritol synthase), CMK (4-diphosphocytidyl-2-C-methyl-D-erythritol kinase), MCS (2-C-methyl-D-erythritol 2,4-cyclodiphosphate synthase), HDS (4-hydroxy-3-methylbut-2-en-1-yl diphosphate synthase), and HDR (4-hydroxy-3-methylbut-2-enyl diphosphate reductase) make up the MEP (Methylerythritol Phosphate) pathway. This pathway is crucial for the synthesis of isoprenoid precursors, which subsequently feed into the tocopherol biosynthetic pathway. MEP is converted to isopentenyl pyrophosphate (IPP) and dimethylallyl pyrophosphate (DMAPP). IPP and DMAPP are condensed by the enzyme geranylgeranyl diphosphate synthase (GGPPS) to produce geranylgeranyl diphosphate (GGPP) (Figure 1). GGPP is reduced to PDP by the action of homogentisate phytyltransferase (HPT). Homogentisate (HGA) is formed from p-hydroxyphenylpyruvate (HPP) by the action of the enzyme HPP dioxygenase (HPPD). HGA and PDP are coupled by the enzyme homogentisate phytyltransferase (HPT) to form 2-methyl-6-phytyl-1,4-benzoquinol (MPBQ) or 2,3-dimethyl-6-phytyl-1,4-benzoquinol (DMPBQ).

Tocopherol Biosynthesis: MPBQ and DMPBQ undergo a cyclization reaction catalyzed by the enzyme tocopherol cyclase (TC) to form γ–tocopherol and δ–tocopherol, respectively. Subsequently, the enzyme γ–tocopherol methyltransferase (γ-TMT), encoded by the gene *vte4*, methylates both γ–tocopherol and δ–tocopherol and converts them into α–tocopherol and β–tocopherol, respectively (Figure 1 [36]).

Tocotrienol Biosynthesis: The enzyme tocopherol cyclase, encoded by the gene *vte1*, converts DMGGBQ and MGGBQ into δ–tocotrienol and δ–tocotrienol, respectively. Subsequently, the enzyme γ–tocopherol methyltransferase (γ-TMT), encoded by the gene *vte4*, methylates both γ–tocotrienol and δ–tocotrienol and converts them into α–tocotrienol and β–tocotrienol, respectively (Figure 1 [36]).

## 3. QTLs That Control Seed Tocopherol Contents

Two mapping population crosses between cultivars that differ in their seed α–Toc and γ–Toc contents were analyzed and genotyped with simple sequence repeat (SSR) and restriction fragment length polymorphism (RFLP) markers, and a software package was used to identify QTL that control seed α–Toc and γ–Toc contents [28]. Eight QTL that control seed α–Toc and γ–Toc contents have been identified on chromosomes 1, 4, 5, 6, and 7 in one or both populations (Table S1; Figure S1) [37].

In a previous study, two distinct population sets were employed [29]. The first population was composed of Recombinant Inbred Lines (RILs), which were generated from a cross between ‘W64a’ (known for its high seed γ–Toc content) and ‘A632’ (recognized for its high seed α–Toc content) lines, with a total of 200 individuals (*n* = 200). The second population resulted from crossing the 200 F_2_ plants with the ‘AE335’ line, characterized by its high seed oil content, to create the testcross population of 185 individuals (*n* = 185). Both populations were meticulously assessed in Urbana, IL, over two-year spans, which were from 1996 to 1997 for the RIL population and from 1999 to 2000 for the testcross population. The research approach involved employing WinQTL Cartographer’s Composite Interval Mapping (CIM) to identify Quantitative trait loci (QTL) responsible for regulating seed α–Toc, γ–Toc, and δ–Toc contents and the α–Toc/γ–Toc ratio. In the F_2_ population, they successfully pinpointed and mapped four QTL controlling seed α–Toc content, which were distributed across chromosomes 5, 6, and 8. Additionally, they identified and mapped four QTL governing seed γ–Toc content, which were located on chromosomes 1, 2, 5, and 7. Furthermore, three QTL governing seed δ–Toc content were identified and mapped on chromosomes 1, 5, and 8. Finally, they detected one QTL controlling seed T–Toc content and the (α/γ)–Toc ratio, which was mapped on chromosome 5. In the testcross population, Wong et al. identified five QTL regulating seed α–Toc content, which were distributed across chromosomes 3, 5, and 6. They also located three QTL governing seed γ–Toc content, which were mapped on chromosomes 5 and 7. Furthermore, one QTL controlling seed δ–Toc content was identified and mapped on chromosome 5. In addition, they discovered three QTL for seed T–Toc content, which were located on chromosomes 5 and 7, and two QTL for seed (α/γ)–Toc ratio on chromosome 5 (Table S1 and Figure S1) [29].

In their study, a Recombinant Inbred Line (RIL) population was cultivated that was developed from a cross between ‘By804’ and ‘B73’ lines, consisting of 233 individuals, in China, spanning a two-year period from 2004 to 2005. They subjected this population to genotyping using 208 markers and employed Composite Interval Mapping (CIM) with WinQTL Cartographer to pinpoint Quantitative trait loci (QTL) responsible for regulating seed α–Toc, γ–Toc, δ–Toc, and T–Toc contents. Their analysis led to the identification and mapping of seven QTL controlling seed α–Toc content, which were distributed across chromosomes 1, 2, 5, 8, 9, and 10. Additionally, they identified and mapped seven QTL governing seed γ–Toc content, which were located on chromosomes 1, 2, 5, and 8. Furthermore, four QTL controlling seed δ–Toc content were identified and mapped on chromosomes 6, 7, and 8. In addition, they detected eight QTL regulating seed T–Toc content that were distributed across chromosomes 1, 2, 5, 8, and 9. Lastly, they found five QTL controlling seed (α/γ)–Toc ratio, which were mapped on chromosomes 3, 5, and 7, as detailed in Table S1 and Figure S1. In a related aspect of their research, Chander et al. (2008) also identified five candidate genes (P3VTE5, HPPD, VTE3, VTE4, and PSY1) involved in tocopherol biosynthesis within the genomic regions harboring the QTL responsible for regulating seed tocopherol contents. These findings offer valuable insights into the genetic factors influencing tocopherol content in seeds [24].

In 2009, a study was conducted using a natural population comprising 543 individuals in three different locations across China. This population was subjected to genotyping with a set of 56,110 SNPs using the MaizeSNP50 BeadChip. The researchers employed a Genome-Wide Association Study (GWAS) approach to identify SNPs associated with Quantitative trait loci (QTL) responsible for governing seed α–Toc, γ–Toc, δ–Toc, and T–Toc contents. Their investigation led to the identification and mapping of 24 SNPs associated with seed α–Toc content, which were distributed across chromosomes 1, 3, 4, and 5. Additionally, they discovered five SNPs linked to seed δ–Toc content, which were mapped on chromosomes 2, 3, 4, 5, and 9. Furthermore, three SNPs were associated with seed γ–Toc content, which were located on chromosomes 1, 3, and 5. They also identified six SNPs associated with seed total tocopherol (T–Toc) content, which were mapped on chromosomes 1, 2, 3, 4, 5, and 8, as detailed in Table S1 and Figure S1. Among the 24 SNPs related to seed α–Toc content, nine were of particular significance and were concentrated within a 2.4 Mb genomic region on chromosome 5. Notably, three of these highly significant SNPs were found to be situated within the ZmVTE4 gene, which encodes γ–tocopherol methyltransferase [38].

In 2009, a study involving two F_2_ populations, one resulting from a cross between K22 and CI7 (comprising 237 individuals) and the other from a cross between K22 and Dan340 (comprising 218 individuals (*n* = 218)), was conducted. These populations were grown in two separate locations in China. The researchers carried out genotyping for each population using a set of 1536 SNPs and subsequently employed a Genome-Wide Association Study (GWAS) to identify SNPs associated with Quantitative trait loci (QTL) responsible for governing seed α–Toc, γ–Toc, δ–Toc, and T–Toc contents. Within the K22 by CI7 F_2_ population (*n* = 237), they identified and mapped nine QTL controlling seed α–Toc content, which were distributed across chromosomes 2, 5, 6, and 7. Similarly, nine QTL were discovered that regulated seed γ–Toc content, and these were mapped on chromosomes 1, 5, and 6. Furthermore, eight QTL controlling seed T–Toc content were identified and mapped on chromosomes 1, 2, 5, and 7. Additionally, eight QTL responsible for seed (α/γ)–Toc ratio were identified and mapped on chromosomes 5, 6, and 8. These findings can be further explored in Table S1 and Figure S1 of Shutu et al.’s 2012 research. In the K22 by Dan340 F_2_ population (*n* = 218), the researchers identified six QTL governing seed α–Toc content, mapped on chromosomes 1, 5, 8, and 10. They also detected nine QTL associated with seed γ–Toc content, which were mapped on chromosomes 1, 2, 5, and 8. Moreover, eight QTL controlling seed T–Toc content were identified and mapped on chromosomes 1, 2, and 5. Additionally, seven QTL influencing seed (α/γ)–Toc ratio were identified and mapped on chromosomes 1, 5, and 8. These results are detailed in Table S1, Figure S1 [39].

In 2011, a study involving an F_2_ population of sweet corn was conducted, which originated from a cross between the ‘A6’ and ‘A57’ lines, comprising a total of 229 individuals in China. The researchers carried out genotyping for this population using 512 SSR markers and subsequently employed Composite Interval Mapping (CIM) with WinQTL Cartographer to pinpoint Quantitative trait loci (QTL) responsible for regulating seed α–Toc, γ–Toc, δ–Toc, and T–Toc contents. In their investigation, they successfully identified and mapped two QTL governing seed α–Toc content, which were located on chromosomes 1 and 2. Additionally, they discovered one QTL responsible for seed δ–Toc content, which was mapped on chromosome 10. Furthermore, two QTL controlling seed γ–Toc content were identified and mapped on chromosomes 1 and 5. In the case of seed T–Toc content, they identified and mapped four QTL on chromosomes 1, 5, and 6. Lastly, two QTL influencing seed (α/γ)–Toc ratio were identified and mapped on chromosomes 1 and 2. Comprehensive details of these findings are available in Table S1, Figure S1, and ref. [40].

In 2009–2010, a comprehensive study involving an association panel of 252 lines was conducted, each carrying alleles representing a wide range of tropical and temperate regions worldwide. This research was carried out in West Lafayette, IN, USA. The panel was subjected to genotyping using an extensive set of 294,092 SNPs, and the researchers employed Genome-Wide Association Study (GWAS) techniques to identify SNPs linked to Quantitative trait loci (QTL) controlling the seed contents of 20 tocopherol-related traits. These traits included γ–tocotrienol (γT3), δ–tocopherol (δT), total tocotrienols (total T3), δ–tocotrienol (δT3), total tocopherols (total T), α–tocopherol (αT), α–tocotrienol (αT3), γ–tocopherol (γT),and total tocochromanol (total T3 + T) contents, as well as their ratios, including δT/αT, δT/γT, δT3/γT3, δT3/αT3, αT/γT, αT3/γT3, δT/(γT + αT), γT/ (γT + αT), γT3/(γT3 + αT3), δT3/(γT3 + αT3), and total T/total T3. Among the vast array of SNPs examined, the researchers identified a total of 146 SNPs, with 88 of them showing significant associations with QTL governing one or more of the 20 tocopherol-related traits. These SNPs were mapped onto all ten maize chromosomes apart from chromosome 4, as detailed in Table S1. Notably, a distinct cluster of SNPs located on chromosome 5 exhibited strong associations with αT and its related ratios. This genomic region harbors the candidate gene GRMZM2G035213, also known as ZmVTE4, encoding γ–tocopherol methyltransferase (γ–TMT). Additionally, within the same region, other candidate genes were identified, including GRMZM2G161641 (encoding an amino acid permease), GRMZM5G823157 (encoding a WYRKY transcription factor), and GRMZM2G325019 (encoding a pentatricopeptide repeat-containing (PPR) protein). Furthermore, the study revealed the presence of the candidate gene GRMZM5G833760, which is responsible for encoding a phytosulfokine receptor, within the QTL region controlling seed γT3 content on chromosome 9. A detailed summary of these findings is provided in Table S1, Figure S1, and ref. [41].

In their study, the high tocopherol line SY999 was utilized and a series of backcrosses with four different maize lines were conducted, namely K140, K185, M01, and M14. The objective was to introduce the ZmVTE4 gene into these lines and enhance their tocopherol content through marker-assisted backcrossing. The outcome of their efforts resulted in a significant increase in seed tocopherol contents for three out of the four lines, namely K140, K185, and M01 [42].

In 2009–2010, Diepenbrock et al. (2017) conducted a comprehensive study involving two distinct groups: a Nested Association Mapping (NAM) population consisting of 5000 individuals and an association panel comprising 281 individuals. The NAM population was generated through crosses between the ‘B73’ line and 25 inbred lines, leading to the creation of 25 families, each containing 200 Recombinant Inbred Lines (RILs). Genotyping was carried out for these populations using a set of 14,772 markers, including 1106 Single Nucleotide Polymorphisms (SNPs). The researchers utilized both Joint Linkage (JL) and Genome-Wide Association Study (GWAS) analyses to identify Quantitative trait loci (QTL) responsible for controlling the seed contents of ten tocopherol-related traits. The study revealed a total of 162 QTL associated with one or more of these 10 tocopherol-related traits, and they were mapped onto all ten maize chromosomes. Specifically, the researchers identified:

- Thirteen QTL controlling seed α-Tocopherol (αT) on chromosomes 1, 2, 3, 4, 5, 6, 7, 8, and 9.
- Eighteen QTL regulating seed δ-Tocopherol (δT) on chromosomes 1, 2, 3, 4, 5, 6, 7, 9, and 10.
- Twenty-one QTL governing seed γ-Tocopherol (γT) on chromosomes 1, 2, 3, 4, 5, 6, 7, 8, 9, and 10.
- Eighteen QTL controlling seed Total tocopherols (ΣT) on chromosomes 1, 2, 3, 4, 5, 6, 8, 9, and 10.
- Seventeen QTL associated with seed α-Tocotrienol (αT3) on chromosomes 1, 2, 3, 4, 5, 6, 7, 8, 9, and 10.
- Twenty-one QTL regulating seed δ-Tocotrienol (δT3) on chromosomes 1, 3, 4, 5, 6, 7, 8, 9, and 10.
- Fourteen QTL governing seed γ-Tocotrienol (γT3) on chromosomes 1, 2, 3, 4, 5, 6, 7, 8, 9, and 10.
- Twelve QTL controlling seed total tocotrienols (ΣT3) on chromosomes 1, 2, 3, 4, 5, 6, 7, 8, 9, and 10.
- Twenty QTL governing seed total tocochromanols (ΣTT3) on chromosomes 1, 2, 3, 4, 5, 6, 7, 8, 9, and 10.
- Eight QTL regulating seed plastochromanol-8 (PC-8) on chromosomes 1, 2, 3, 5, 6, 7, and 9.

Additionally, the study identified numerous candidate genes within these QTL regions, including genes coding for γ–tocopherol methyltransferase (VTE4), Arogenate/prephenate dehydrogenase family protein, ABC kinase (tocopherol cyclase kinase), 1-deoxy-D-xylulose 5-phosphate synthase 1 (dxs1), 1-deoxy-D-xylulose 5-phosphate synthase 2 (dxs2), chorismate mutase, homogentisic acid geranylgeranyl transferase 1 (HGGT1), shikimate biosynthesis protein, 4-hydroxyphenylpyruvate dioxygenase, isopentenyl pyrophosphate isomerase, 3-deoxy-d-arabino-heptulosonate 7-phosphate synthase, and MPBQ/MSBQ methyl transferase (VTE3), among other protein. Further details can be found in Table S1 and study [43].

In 2014–2015, a study was conducted involving an association panel consisting of 384 sweet corn lines. These lines were cultivated in Aurora, NY, and subjected to genotyping using an extensive set of 174,996 SNPs. The researchers employed Genome-Wide Association Study (GWAS) analysis to pinpoint Quantitative trait loci (QTL) responsible for controlling the seed contents of various tocopherol-related traits, including α-tocotrienol (αT3), δ-tocotrienol (δT3), γ-tocotrienol (γT3), α-tocopherol (αT), δ-tocopherol (δT), γ-tocopherol (γT), total tocotrienols (totalT3), total T/total T3, total tocopherols (total T), δT/(γT + αT), total tocochromanols (total T3 + T), δT3/(γT3 + αT3), δT3/γT3, αT/γT, γT3/(γT3 + αT3), δT3/αT3, δT/γT, δT/αT, αT3/γT3, and γT/(γT + αT) contents, which were each measured in μg g-1 of fresh seed kernel. The study successfully identified a total of 336 SNPs that exhibited significant associations with one or more of these 20 tocopherol-related traits [36].

In 2012–2013, experiments were conducted in West Lafayette, IN, where they cultivated two Recombinant Inbred Line (RIL) populations: N6 by NC296 RIL population (consisting of 213 individuals) and E2558 by Co125 RIL population (comprising 197 individuals). Initially, a total of 955,690 Single Nucleotide Polymorphisms (SNPs) were genotyped for these populations. However, following data refinement, the Genome-Wide Association Study (GWAS) analysis was conducted using 89,125 SNPs for the N6 by NC296 RIL population and 76,081 SNPs for the E2558 by Co125 RIL population. The primary objective was to identify SNPs associated with Quantitative trait loci (QTL) responsible for regulating the content of six tocopherol-related traits in seeds, namely α-tocotrienol (αT3), α-tocopherol (αT), δ-tocopherol (δT), δ-tocotrienol (δT3), γ-tocopherol (γT), and γ-tocotrienol (γT3) contents. In the N6 by NC296 RIL population, a total of 31 QTL responsible for controlling the seed contents of these six tocopherol-related traits were successfully identified and mapped onto chromosomes 1, 5, 6, 7, and 9. In contrast, in the Co125 RIL population, they identified 58 QTL controlling the seed contents of these same traits, which were mapped onto chromosomes 1, 2, 3, 4, 5, 6, 7, 8, 9, and 10 (as illustrated in Table S1 and Figure S1). Furthermore, within these QTL regions, several candidate genes were pinpointed, including *ZmVTE1* on chromosome 5, which encodes a tocopherol cyclase; ZmVTE2 on chromosome 9, which encodes a homogentisate phytyltransferase; ZmVTE4 on chromosome 5, which encodes a γ-T methyltransferase; ZmHPPD1 on chromosome 5, which is responsible for encoding a hydroxyphenylpyruvate deoxygenase; and ZmHGGT1 on chromosome 9, which encodes a homogentisic acid geranyl transferase (as listed in Table S1) [37].

From 2009 to 2011, a study was conducted in China involving seven different populations. One of these populations comprised an association panel of inbred corn lines from across China totaling 508 individuals. They were subjected to genotyping with 56,110 SNPs, and the researchers employed GWAS analysis to pinpoint Quantitative trait loci (QTL) responsible for regulating seed contents of α–tocopherol (AT), γ–tocopherol (GT), δ–tocopherol (DT), and total tocopherol (TT) and the (α/γ)-tocopherol ratio. The remaining populations were composed of Recombinant Inbred Line (RIL) populations, each consisting of approximately 200 lines. For these biparental RIL populations, the researchers utilized CIM in WinQTL Cartographer to identify QTL governing seed contents of α–tocopherol (AT), γ–tocopherol (GT), δ–tocopherol (DT), and total tocopherol (TT) and the (α/γ)-tocopherol ratio (RT). In these RIL populations, the researchers successfully identified and mapped a total of 41 QTL that control one or more of the five tocopherol-related traits, spanning all chromosomes (1–10). Among these QTL, ten were novel, and six of them were considered major QTL, explaining 10% or more of the phenotypic variation in seed kernel tocopherol content. In the natural population, 151 SNPs associated with QTL regulating seed AT were identified and mapped on chromosomes 1, 2, 3, 4, and 5. Additionally, 17 SNPs linked to QTL controlling seed GT were identified and mapped on chromosomes 1, 5, 6, and 8. Moreover, 61 SNPs were associated with QTL governing seed TT and were mapped on chromosomes 1, 2, 3, 4, 5, 6, 8, and 10. Lastly, 51 SNPs were linked to QTL controlling seed RT, and they were identified and mapped on chromosomes 2, 4, 5, 6, 9, and 10, as detailed in Table S1. Furthermore, numerous candidate genes were discovered within these QTL regions, including genes like GRMZM2G436226 on chromosome 1, which encodes a Zinc finger CCCH domain-containing protein; GRMZM2G055752 and GRMZM2G079236 on chromosome 2, which encode a DNA topoisomerase and an Acyl-CoA synthetase long-chain family member, respectively; GRMZM2G170013 on chromosome 3, which encodes a chlorophyll b reductase NYC1; and GRMZM5G892742 and GRMZM2G150714 on chromosome 4, which encode a DNAJ-related chaperone protein and a putative leucine-rich repeat receptor-like protein kinase family protein, respectively. Similar candidate genes were identified on other chromosomes (5–10), as listed in Table S1 [44].

In 2015–2016, a study was conducted in China that cultivated an association panel consisting of 204 inbred corn lines. They performed genotyping on this panel using 150,124 SNPs and subsequently employed GWAS analysis to pinpoint Quantitative trait loci (QTL) responsible for governing the seed contents of eight vitamin E–related traits. These traits included seed α-tocopherol (AT), γ-tocopherol (GT), δ-tocopherol (DT), β-tocopherol (BT), α-tocotrienol (AT3), γ-tocotrienol (GT3), and δ-tocotrienol (DT3). The study successfully identified a total of 119 loci that were significantly associated with the seed content of these vitamin E–related traits. Specifically, they discovered 9 QTL (qAT-1 to qAT-9) related to AT on chromosomes 1, 2, 5, and 6; 32 QTL (qBT-1 to qBT-32) linked to BT on chromosomes 1, 2, 3, 4, 5, 6, 7, and 9; 1 QTL (qDT-1) associated with DT on chromosome 4; 13 QTL (qRT-1 to qRT-13) governing RT on chromosomes 1, 2, 5, and 9; 4 QTL (qTT-1 to qTT-4) controlling TT on chromosomes 2, 5, and 6; 3 QTL (qAT3-1 to qAT3-3) affecting AT3 on chromosomes 4 and 5; 56 QTL (qRT3-1 to qRT3-56) influencing RT3 on chromosomes 1, 2, 3, 4, 5, 6, 7, 8, 9, and 10; and 1 QTL (qTT3-1) regulating TT3 on chromosome 7, as detailed in Table S1 and (Xiao et al., 2020) study [45].

In 2014–2015, a study was conducted at Cornell University in Aurora, NY, where they cultivated an association panel comprising 384 inbred sweet corn lines. Subsequently, they performed genotyping on this panel using 174,996 SNPs and employed GWAS analysis to pinpoint Quantitative trait loci (QTL) responsible for regulating the contents of α-tocopherol (αT), γ-tocopherol (γT), δ-tocopherol (δT), α-tocotrienol (αT3), γ-tocotrienol (γT3), and δ-tocotrienol (δT3). The study successfully identified a total of 336 SNPs that exhibited significant associations with seed tocopherol traits. These SNPs were primarily mapped to chromosomes 2, 3, 4, 5, 6, 7, and 8. Furthermore, these SNPs were linked to the genes vte1, which encodes tocopherol cyclase; vte4, responsible for encoding γ-tocopherol methyltransferase; and hggt1, which encodes homogentisate geranylgeranyltransferase [36].

In 2011–2012, Alves et al. (2020) conducted a study in Portugal that grew an association panel comprising 132 inbred corn lines. They performed genotyping on this panel using 48,778 SNPs and applied GWAS analysis to identify Quantitative trait loci (QTL) governing the seed contents of α-tocopherol (AT), γ-tocopherol (GT), and δ-tocopherol (DT). Additionally, they investigated seed carotenoid, phenolic compound, and hydroxycinnamic acid contents. The study successfully identified and mapped a total of 14 QTL controlling seed AT (ATLOG) content, located on chromosomes 4, 5, and 10. They also found 19 QTL regulating seed DT (DTLOG) content, which were distributed across chromosomes 1, 2, 3, 4, 5, 6, and 7. Furthermore, five QTL governing seed GT content were identified and mapped to chromosomes 1, 4, and 8, as detailed in Table S1 and [46].

In a recent study by Hershberger et al. (2022), an association panel of Recombinant Inbred Lines (RILs) consisting of 382 individuals, as previously described by Baseggio et al. (2019), was grown in Aurora, NY, in 2019. Genotyping was performed using 174,966 SNPs, and transcriptome-wide association studies (TWAS) were used to identify candidate genes responsible for regulating seed carotenoid and tocochromanol traits, which included α-tocopherol (αT), α-tocotrienol (αT3), γ-tocopherol (γT), γ-tocotrienol (γT3), δ-tocopherol (δT), δ-tocotrienol (δT3), and their cumulative total contents [36]. In this recent study, four genes were identified as associated with these traits: γ-tocopherol methyltransferase (vte4), lycopene epsilon cyclase (lcyE), β-carotene hydroxylase (crtRB1), and homogentisate geranylgeranyltransferase (hggt1) [47].

## 4. Seed Tocopherol Candidate Genes

As mentioned earlier, Li et al. (2012) identified 24 SNPs associated with seed α–, δ–, γ–, and total tocopherol contents, and 3 of these SNPs fell within the *ZmVTE4* gene encoding γ–tocopherol methyltransferase (Table S1) [38]. Lipka et al. (2013) identified 88 SNPs associated with tocopherol-related traits among which a strong cluster of SNPs on chromosome 5 that contains the candidate gene *GRMZM2G035213* (*ZmVTE4*) encoding γ–tocopherol methyltransferase (γ–TMT). Other candidate genes have been identified in the same region such as *GRMZM2G161641* encoding an amino acid permease, *GRMZM5G823157* encoding a WYRKY transcription factor, and *GRMZM2G325019* encoding a pentatricopeptide repeat-containing (PPR) protein. The candidate gene *GRMZM5G833760* that encodes a phytosulfokine receptor has been identified within the QTL region that controls seed γT3 content on chromosome 9 (Table S1) [41]. Diepenbrock et al. (2017) identified 162 QTL that control seed tocopherol-related traits and many candidate genes have been identified within these QTL regions, including genes that encode γ–tocopherol methyltransferase (*VTE4*), Arogenate/prephenate dehydrogenase family protein, ABC kinase (tocopherol cyclase kinase), 1-deoxy-D-xylulose 5-phosphate synthase 1 (*dxs1*), 1-deoxy-D-xylulose 5-phosphate synthase 2 (*dxs2*), chorismate mutase, homogentisic acid geranylgeranyl transferase 1 (*HGGT1*), shikimate biosynthesis protein, 4-hydroxyphenylpyruvate dioxygenase, isopentenyl pyrophosphate isomerase, 3-deoxy-d-arabino-heptulosonate 7-phosphate synthase, MPBQ/MSBQ methyl transferase (*VTE3*), and many other proteins (Table S1) [43]. Fenton et al. (2018) identified 81 QTL that control seed contents of six tocopherol-related traits in two RIL populations and several candidate genes within these QTL regions, including *ZmVTE1* that encodes a tocopherol cyclase chromosome 5; *ZmVTE2* that encodes a homogentisate phytyltransferase on chromosome 9; ZmVTE4 that encodes a γ-T methyltransferase on chromosome 5; *ZmHPPD1* that encodes a hydroxyphenylpyruvate deoxygenase on chromosome 5; and *ZmHGGT1* that encodes a homogentisic acid geranyl transferase on chromosome 9 (Table S1) [37]. Wang et al. (2018) identified 41 QTL and 151 SNPs associated with seed tocopherol-related traits and many candidate genes within these QTL regions, including *GRMZM2G436226* that encodes a Zinc finger CCCH domain-containing protein on chromosome 1, *GRMZM2G055752* that encodes a DNA topoisomerase and *GRMZM2G079236* that encodes a Acyl-CoA synthetase long-chain family member on chromosome 2, *GRMZM2G170013* that encodes a chlorophyll b reductase NYC1 on chromosome 3, GRMZM5G892742 that encodes a DNAJ-related chaperone protein and *GRMZM2G150714* that encodes a putative leucine-rich repeat receptor-like protein kinase family protein on chromosome 4, *GRMZM2G035213* that encodes a γ-tocopherol methyltransferase (*VTE4*) and *GRMZM2G024739* that encodes a Cryptochrome-1 (*CRY1*) on chromosome 5, *GRMZM2G127299* that encodes a transcription factor GTE8 and *GRMZM2G146190* that encodes a peptidyl-prolyl cis-trans isomerase (CYP59) on chromosome 6, *GRMZM2G003022* that encodes a COPII vesicle coat on chromosome 8, *GRMZM5G818232* that encodes a Clavata3/esr-related16 on chromosome 9, *GRMZM2G004534* that encodes a pyruvate kinase 2 on chromosome 10, and many other genes (Table S1) [44]. Zhang et al. (2019) created transgenic seeds of maize and *Arabidopsis thaliana* and overexpressed the gene *ZmTMT* (*VTE4*) that encodes γ–tocopherol methyltransferase in those seeds and observed a 4–5-fold increase in α–tocopherol content in *Arabidopsis* and 6.5-fold increase in maize transgenic plants while the (α/γ)–tocopherol ratio increased by 15-fold and 17-fold, respectively, which proves that increasing tocopherol content in corn is feasible [48]. Zhan et al. (2019) grew three RIL populations (*n* = 170–188 lines) from the crosses of Zong3 by Yu8701 (ZXY), K22XCI7, and B73 by BY804 (BXB) in two environments in China and genotyped them with 50 k SNPs. They used CIM of WinQTLCart2.5 for QTL analysis and identified one major QTL that controls seed γ–tocopherol and total tocopherol (qVE5) on chromosome 5 [48]. The major QTL qVE5 was also identified in a previous study [44] and confirmed here in these three RIL populations [49]. This qVE5 genomic region between NF129 and NF11 markers was narrowed to 170 kb within which the candidate gene GRMZM2G073351 that encodes a chloroplastic protochlorophyllide oxidoreductase (ZmPORB2) has been identified, and its overexpression increases tocopherol contents in both seeds and leaves, which was determined to occur mainly by maternal effect [49]. Most genes involved in domestication-related traits in maize are summarized by Liu et al. (2020) [50].

As mentioned earlier, Baseggio et al. (2019) identified 336 SNPs significantly associated with seed tocopherol traits on chromosomes 2, 3, 4, 5, 6, 7, and 8. Within these genomic regions, the SNPs identified the genes *vte1* encoding tocopherol cyclase, *vte4* encoding γ-tocopherol methyltransferase, and *hggt1* encoding homogentisate geranylgeranyltransferase [36].

It is well established that γ–tocopherol methyltransferase (γ–TMT, encoded by the gene VTE4) synthesizes α-tocopherol from γ–tocopherol. In a recent study, Zhang et al. (2020) isolated the γ–TMT coding sequence (1059 pb), called it ZmTMT, and demonstrated that its expression in transgenic plants of *A. thaliana* increased the production of α-Toc from γ–Toc by 4- to 5-fold while α-Toc content increased by 5- to 6-fold in corn seed kernels [51]. Dai et al. (2021) summarized maize QTL and genes involved in seed kernel development (*Dek*), embryo development (*emb*), starch-related genes, tocopherol biosynthesis, yield, seed oil, protein, fatty acids contents, photosynthesis optimization, genes involved in RNA processing, ribosome biogenesis, and many other processes [52]. Interestingly, a recent study identified a gene (AC212835.3_FG001) involved in popping expansion that is located within the region that harbors a pentatricopeptide repeat (PPR) protein involved in seed tocopherol and amylose biosynthesis [53]. In a recent study, Hershberger et al. (2022) grew an association panel of RILs (*n* = 382) [47] as described earlier [36] in 2019 in Aurora, NY, and they genotyped it with 174,966 SNPs and used transcriptome-wide association studies (TWAS) to identify candidate genes that control seed carotenoid and tocochromanol (vitamin E and antioxidants) traits (α-tocopherol, αT, α-tocotrienol, αT3, γ-tocopherol, γT, γ-tocotrienol, γT3, δ-tocopherol, δT, δ-tocotrienol, and δT3 contents and their totals) [36]. They identified four genes associated with these traits: γ-tocopherol methyltransferase (vte4), lycopene epsilon cyclase (lcyE), β-carotene hydroxylase (crtRB1), and homogentisate geranylgeranyltransferase (hggt1) [47].

## 5. Expression Analysis of the Candidate Gene Involved in the Tocopherol and Tocotrienol Biosynthetic Pathway

The tocopherol and tocotrienol biosynthetic pathway, including the genes and compounds involved, was previously reported in the model plant *A. thaliana* [36,54]. The reconstruction of this pathway in *Z. mays* was conducted using the reverse BLAST of the previously reported genes in *A. thaliana* [54].

**Figure 2:**
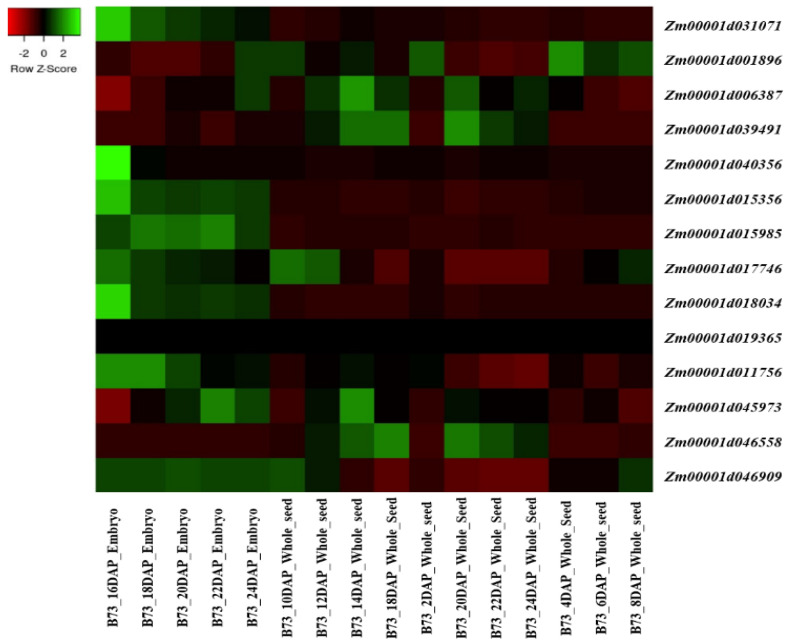
Seed and embryo expression heatmap of the tocopherol and tocotrienol biosynthesis candidate genes in *Z. mays*.

In total, 14 genes underlying the tocopherol and tocotrienol pathway in *Z. mays* were identified (Figure 2, Table 1). HGGT is present only in monocots [55], and not in Arabidopsis. The gene ID of this gene in *Z. mays* was obtained by searching the available data at Phytozome database (https://phytozome-next.jgi.doe.gov/, accessed on 7 April 2024). The name of the gene was used as a query in a search of the *Z. mays* reference genome (*Z. mays RefGen_V4*), and the obtained genes are represented in Figure 2 and Table 1.

The publicly available RNA-seq database from MaizeGDB [56] was used to perform expression analysis of the genes involved in the corn tocopherol and tocotrienol biosynthetic pathway. The expression pattern of these genes in corn seeds and embryos was produced in version 4 of Gene Model ID. The obtained data was converted into a heatmap using the Heatmapper website [57]. Most of the analyzed genes were expressed in the analyzed tissues except for the ZmHPPD1 gene, *Zm00001d019365*, that was not expressed in any of the analyzed tissues (Figure 2). In contrast, the *ZmVTE3* gene, *Zm00001d031071*, and the *ZmGGDR* gene, *Zm00001d018034*, were highly expressed in the embryo. Five genes had moderate expression profiles, including the *ZmHPPD1* gene, *Zm00001d015356*; the *ZmGGDR* gene, *Zm00001d040356*; the *ZmVTE5* gene, *Zm00001d001896*; the *ZmVTE1* gene, *Zm00001d015985*; and the *ZmVTE2* gene, *Zm00001d046909*. The remaining genes presented lower expression patterns (Figure 2).

Three genes presented a moderate expression pattern in the seeds, including the *ZmVTE2* gene, *Zm00001d046909*; the *ZmVTE3* gene, *Zm00001d031071*; and the *ZmVTE5* gene, *Zm00001d001896*. The remaining genes presented lower expression profiles (Figure 2).

The QTL and candidate genes identified by these studies may be used in MAS to develop maize cultivars with high seed tocopherol contents.

## 6. Conclusions

In conclusion, the exploration of Quantitative trait loci (QTL) and candidate genes associated with seed tocopherol contents in maize (*Z. mays* L.) has significantly advanced our understanding of the genetic bases underpinning the biosynthesis and accumulation of tocopherols. The identification and mapping of numerous QTL across diverse maize populations and environments underscore the complex polygenic nature of tocopherol content, which is influenced by both genetic and environmental factors. Studies utilizing mapping populations, Recombinant Inbred Lines (RILs), and genome-wide association studies (GWAS) have identified critical genomic regions and candidate genes that contribute to variations in α–Toc, β–Toc, γ–Toc, δ–Toc, and total tocopherol contents. Notably, genes such as ZmVTE4, which encodes γ–tocopherol methyltransferase, have been highlighted for their central role in converting γ–tocopherol to α–tocopherol, which is the most bioactive form of vitamin E.

The discovery of these QTL and candidate genes not only enriches our genetic knowledge of tocopherol biosynthesis in maize but also opens avenues for marker-assisted selection (MAS) and genetic improvement strategies aimed at enhancing tocopherol content in maize seeds. Such genetic improvements hold promise for developing maize cultivars with increased nutritional quality, offering potential health benefits to consumers and adding value to maize as a crop. Moreover, the identification of specific genes and their expression patterns provides a molecular foundation for further research into the regulatory mechanisms controlling tocopherol biosynthesis, offering insights that could be leveraged across different plant species.

Future research efforts should focus on fine-mapping identified QTL, validating the function of candidate genes, and elucidating the complex regulatory networks that govern tocopherol accumulation. Additionally, integrating genomic, transcriptomic, and metabolomic data could yield comprehensive insights into the tocopherol biosynthetic pathway, facilitating the development of biofortified maize varieties that are tailored to meet nutritional requirements and adapt to changing environmental conditions. Ultimately, the integration of genetic, molecular, and breeding approaches will be crucial in harnessing the full potential of maize tocopherols for improving crop nutritional quality and addressing global health challenges.

## Figures and Tables

**Table 1:** Genes involved in the corn tocopherol and tocotrienol biosynthetic pathway.

Gene	v4 Gene Model ID	v2 Gene Model ID	Full Name	Chromosome	Start	End
*ZmVTE4*	*Zm00001d017746*	GRMZM2G035213	vitamin E synthesis 4	5	205825585	205829216
*ZmVTE2*	*Zm00001d046909*	GRMZM2G048472	homogentisate phytyltransferase 1	9	110047718	110058786
*ZmVTE1*	*Zm00001d015985*	GRMZM2G009785	Tocopherol cyclase/sucrose export defective 1 (SXD1)	5	136805707	136822194
*ZmVTE3*	*Zm00001d031071*	GRMZM2G082998	2-methyl-6-phytyl-1,4-hydroquinone methyltransferase/MPBQ/MSBQ methyltransferase	1	175969708	175971563
*ZmHGGT2*	*Zm00001d006387*	GRMZM2G410644	Homogentisate geranylgeranyltransferase/HGGT	2	206364248	206367453
*ZmHGGT3*	*Zm00001d039491*	GRMZM5G848876	Homogentisate geranylgeranyltransferase/HGGT	3	5766073	5768360
*ZmHGGT4*	*Zm00001d045973*	GRMZM2G398628	Homogentisate geranylgeranyltransferase/HGGT	9	51047616	51049885
*ZmHGGT1*	*Zm00001d046558*	GRMZM2G173358	Homogentisate geranylgeranyltransferase/HGGT	9	95895574	95899061
*ZmVTE5*	*Zm00001d001896*	GRMZM2G104538	vte5—vitamin E synthesis 5	2	2509566	2511414
*ZmVTE6*	*Zm00001d011756*	GRMZM2G166383	KOG4491—Predicted membrane protein			
*ZmGGDR*	*Zm00001d018034*	GRMZM2G105741	Geranylgeranyl diphosphate reductase/Geranylgeranyl reductase	5	212543655	212546035
	*Zm00001d040356*	GRMZM2G419111	Geranylgeranyl diphosphate reductase/Geranylgeranyl reductase	3	39647288	39648626
*ZmHPPD1*	*Zm00001d015356*	GRMZM2G088396	hppd1—4-hydroxyphenylpyruvate dioxygenase 1	5	86084654	86086755
	*Zm00001d019365*	GRMZM2G374213	4-hydroxyphenylpyruvate dioxygenase/p-hydroxyphenylpyruvate oxidase	7	29973891	29976259

## Data Availability

The original contributions presented in the study are included in the article/Supplementary Material, further inquiries can be directed to the corresponding author.

## Supplementary Materials

The following supporting information can be downloaded at: https://www.mdpi.com/article/10.3390/genes15040472/s1, Figure S1: Chromosome locations of QTL that control seed α–tocopherol (α–Toc), γ–tocopherol (γ–Toc), δ–tocopherol (δ–Toc), total tocopherol (T–Toc) contents and (α/γ)–Toc ratio in maize from 2002 to 2022.; Table S1: Details of QTL that control seed α–tocopherol (α–Toc), γ–tocopherol (γ–Toc), δ–tocopherol (δ–Toc), total tocopherol (T–Toc) contents and (α/γ)–Toc ratio in maize from 2002 to 2022. Candidate genes identified with these QTL regions are also shown.

## Abbreviations

QTL, quantitative trait loci; α–Toc, α–tocopherol; β–Toc, β–tocopherol; γ–Toc, γ–tocopherol; δ–Toc, δ–tocopherol.
