# Supplementary material for: A Summary of Two Decades of QTL and Candidate Genes That Control Seed Tocopherol Contents in Maize (Zea mays L.)
Source: Genes (Basel). 2024 Apr 9;15(4):472. doi: 10.3390/genes15040472 (PMC11049817; doi:10.3390/genes15040472)
Supplement: Supplementary file 1 [file genes-15-00472-s001.zip › genes-2823121-supplementary.pdf]

Supplementary Data.

Table S1. Details of QTL that control seed α–tocopherol (α–Toc), γ–tocopherol (γ–Toc), δ–tocopherol (δ–Toc), total tocopherol (T–Toc) contents and (α/γ)–Toc ratio in maize from 2002 to 2022. Candidate genes identified with these QTL regions are also shown.

Figure S1. Chromosome locations of QTL that control seed α–tocopherol (α–Toc), γ–tocopherol (γ–Toc), δ–tocopherol (δ–Toc), total tocopherol (T–Toc) contents and (α/γ)–Toc ratio in maize from 2002 to 2022.

Table S1. Details of QTL that control seed α–tocopherol (α–Toc), γ–tocopherol (γ–Toc), δ–tocopherol (δ–Toc), total tocopherol (T–Toc) contents and (α/γ)–Toc ratio in maize from 2002 to 2022. Candidate genes identified with these QTL regions are also shown.

Trait	QTL	Chr.	Marker / Interval	Position (cM)	LOD/P	R^2 (%)	Candid. Gene/Product	Environment	Reference
**α–Toc & γ–Toc**	*qα–Toc-1|qγ–Toc-1*	1	p-umc1073-p-phi109275	81.9-118.4	*	*	*	IL, USA	(Rocheford et al., 2002)
	qα–Toc-2|qγ–Toc-2		p-umc2237-p-umc1245	216.3-258.2	*	*	*	IL, USA	
	qα–Toc-3|qγ–Toc-3	4	p-umc1808-p-umc2188	162.1-181.7	*	*	*	IL, USA	
	qα–Toc-4|qγ–Toc-4	5	p-umc0081-p-umc2304	133.8-141.3	*	*	*	IL, USA	
	qα–Toc-5|qγ–Toc-5		p-umc1019-p-phi085	162.8-193.9	*	*	*	IL, USA	
	qα–Toc-6|qγ–Toc-6		p-phi085-p-phi048	193.9-194.4	*	*	*	IL, USA	
	qα–Toc-7|qγ–Toc-7	6	p-umc1006-p-bnlg2191	24.4-27.2	*	*	*	IL, USA	
	qα–Toc-8|qγ–Toc-8	7	p-umc1713-p-umc1301	107.8-144.5	*	*	*	IL, USA	
			**QTL Identified in (W64a X A632) RIL population (*n*=200)**						(Wong et al., 2003)
**α–Toc**	*qα–Toc-1*	5	mmc0081-umc1155	51.0-53.0	4.2	2.9	*	IL, USA	
	qα–Toc-2		umc1155-phi085	89.0-91.0	13.9	19.6	*	IL, USA	
	qα–Toc-3	6	umc21-bmc1154	93.0-95.0	3.6	2.5	*	IL, USA	
	qα–Toc-4	8	phi115-bmc1176	63.0-65.0	3.1	2.7	*	IL, USA	
**δ–Toc**	*qδ–Toc-1*	1	bmc1016-dupssr026	145.0-147.0	3.9	2.8	*	IL, USA	
	qδ–Toc-2	5	mmc0081-umc1155	55.0-57.0	5.3	9.0	*	IL, USA	
	qδ–Toc-3	8	umc1149-mmc0181	97.0-99.0	3.1	5.3	*	IL, USA	
**γ–Toc**	*qγ–Toc-1*	1	bmc1016-dupssr026	141.0-143.0	3.2	5.4	*	IL, USA	
	qγ–Toc-2	2	dupssr21-umc1156	59.0-61.0	3.2	3.2	*	IL, USA	
	qγ–Toc-3	5	phi113-dupssr010	19.0-21.0	8.7	23.6	*	IL, USA	
	qγ–Toc-4	7	bmc1305-dupssr009	111.0-113	4.4	7.7	*	IL, USA	
**Total-Toc**	*qT-Toc-1*	5	phi113-dupssr010	15.0-17.0	7.4	10.9	*	IL, USA	
**(α/γ)–Toc Ratio**	*q(α/γ)–Toc-1*	5	umc1155-phi085	91.0-93.0	9.4	33.0	*	IL, USA	
			**QTL Identified in (W64a X A632) X AE335 Testcross population (*n*=185)**						
**α–Toc**	*qα–Toc-1*	3	phi046-bnlg197	221.0-223.0	4.3	3.5	*	IL, USA	
	qα–Toc-2	5	mmc0081-umc1155	51.0-53.0	7.7	2.3	*	IL, USA	
	qα–Toc-3		umc1155-phi085	79.0-81.0	6.4	0.4	*	IL, USA	
	qα–Toc-4		phi085-bmc1346	101.0-103.0	12.3	4.7	*	IL, USA	
	qα–Toc-5	6	dupssr015-bmc1136	191.0-193.0	4.0	4.2	*	IL, USA	
**δ–Toc**	*qδ–Toc-1*	5	umc1155-phi085	65.0-67.0	3.5	8.8	*	IL, USA	
**γ–Toc**	*γ–Toc-1*	5	dupssr010-umc1221	35.0-37.0	5.3	4.0	*	IL, USA	
	γ–Toc-2		umc1155-phi085	83.0-85.0	3.9	7.5	*	IL, USA	
	γ–Toc-3	7	bmc1305-dupssr009	111.0-113.0	6.6	11.1	*	IL, USA	
**Total-Toc**	*qT-Toc-1*	5	dupssr010-umc1221	37.0-39.0	3.7	6.4	*	IL, USA	
	qT-Toc-2	7	zdsRFLP-phi034	81.0-83.0	3.3	5.1	*	IL, USA	
**(α/γ)–Toc Ratio**	*q(α/γ)–Toc-1*	5	mmc0081-umc1155	51.0-53.0	8.0	12.8	*	IL, USA	
	q(α/γ)–Toc-2		umc1155-phi085	91.0-93.0	16.8	30.5	*	IL, USA	
**α–Toc**	*qα–Toc-1*	1	umc1598–bnlg1811	79–94	8.3	15.5	*	China	(Chander et al., 2008)
	qα–Toc-2	1	umc1725–phi064	273–280	3.2	4.6	*	China	
	qα–Toc-3	2	P3VTE5–umc1265	3–19	3.1	5.3	*P3VTE5*	China	
	qα–Toc-4	5	bnlg1237–VTE4–phi085	130–140	7.9	13.8	*VTE4*	China	
	qα–Toc-5	8	umc1075–umc1304	17–33	6.1	7.9	*	China	
	qα–Toc-6	9	phi065–umc1258	33–44	6.3	9.6	*	China	
	qα–Toc-7	10	phi118–umc2053	0–5	4.3	5.4	*	China	
**δ–Toc**	*qδ–Toc-1*	6	umc1195–Y1ssr–umc1595	28–38	6.6	9.6	*PSY1*	China	
	qδ–Toc-2	7	mmc0171–VTE3	30–40	5.7	13.5	*VTE3*	China	
	qδ–Toc-3	7	bnlg1792–phi091	45–54	9.0	18.4	*	China	

	qδ–Toc-4	8	umc1075–umc1304	10–29	3.2	11.4	*	China	
**γ–Toc**	*qγ–Toc-1*	1	umc1403–phi001	57–68	5.3	9.5	*	China	
	qγ–Toc-2	1	phi001–bnlg1811	72–85	5.5	8.4	*	China	
	qγ–Toc-3	2	P3VTE5–umc1215	3–17	3.0	5.2	*P3VTE5*	China	
	qγ–Toc-4	5	umc2115–umc1447	58–72	7.0	16.4	*	China	
	qγ–Toc-5	5	umc1692–HPPD	76–84	4.6	7.1	*HPPD*	China	
	qγ–Toc-6	5	bnlg1237–VTE4–phi085	131–141	4.2	5.6	*VTE4*	China	
	qγ–Toc-7	8	umc1075–umc1304	16–31	11.2	29.3	*	China	
**T-Toc**	*qT-Toc-1*	1	umc1403–phi001	59–69	7.0	13.7	*	China	
	qT-Toc-2	1	phi001–umc1598	72–86	7.7	12.3	*	China	
	qT-Toc-3	2	P3VTE5–umc1215	3–16	3.4	7.0	*P3VTE5*	China	
	qT-Toc-4	5	umc2115–umc1447	59–73	5.6	11.7	*	China	
	qT-Toc-5	5	umc1692–HPPD	76–84	4.5	7.1	*HPPD*	China	
	qT-Toc-6	8	umc1075–umc1304	12–30	14.1	33.5	*	China	
	qT-Toc-7	9	umc1258–umc1688	39–45	3.0	3.6	*	China	
	qT-Toc-8	9	umc1078–umc1471	54–63	3.9	6.9	*	China	
**(α/γ)–Toc Ratio**	*q(α/γ)–Toc-1*	3	phi053–dupssr23	101–111	3.0	3.3	*	China	
	q(α/γ)–Toc-2	5	umc2115–umc1447	59–74	6.8	9.0	*	China	
	q(α/γ)–Toc-3	5	umc1692–HPPD	75–84	6.3	9.3	*HPPD*	China	
	q(α/γ)–Toc-4	5	bnlg1237–VTE4–phi085	134–138	10.2	13.3	*VTE4*	China	
	q(α/γ)–Toc-5	7	umc1112–umc2332	73–86	3.2	6.7	*	China	
**α–Toc**	*qα–Toc-1*	1	A/G-41,597	3,009,997	*	*	*	China	(Li et al., 2012)
	qα–Toc-2	1	A/C-54,850	3,379,175	*	*	*	China	
	qα–Toc-3	1	A/G-1,213	60,212,244	*	*	*	China	
	qα–Toc-4	3	A/T-17,550	228,955,373	*	*	*	China	
	qα–Toc-5	4	C/G-18,772	65,286,187	*	*	*	China	
	qα–Toc-6	4	A/G-18,773	65,286,238	*	*	*	China	
	qα–Toc-7	5	A/G-25,801	198,800,752	*	*	*ZmVTE4*	China	
	qα–Toc-8	5	A/G-25,814	199,266,329	*	*	*ZmVTE4*	China	
	qα–Toc-9	5	T/C-25,815	199,442,506	*	*	*ZmVTE4*	China	
	qα–Toc-10	5	A/G-49,239	199,444,812	*	*	*ZmVTE4*	China	
	qα–Toc-11	5	A/G-25,817	199,461,718	*	*	*ZmVTE4*	China	
	qα–Toc-12	5	A/G-3,462	199,528,414	*	*	*ZmVTE4*	China	
	qα–Toc-13	5	T/C-25,820	199,530,006	*	*	*ZmVTE4*	China	
	qα–Toc-14	5	A/G-25,821	199,530,947	*	*	*ZmVTE4*	China	
	qα–Toc-15	5	A/G-408	199,970,863	*	*	*ZmVTE4*	China	
	qα–Toc-16	5	T/C-53,345	200,017,666	*	*	*ZmVTE4*	China	
	qα–Toc-17	5	T/G-25,826	200,023,434	*	*	*ZmVTE4*	China	
	qα–Toc-18	5	A/G-45,458	200,776,518	*	*	*ZmVTE4*	China	
	qα–Toc-19	5	A/G-51,039	201,207,792	*	*	*ZmVTE4*	China	
	qα–Toc-20	5	T/G-51,038	201,211,449	*	*	*ZmVTE4*	China	
	qα–Toc-21	5	T/C-51,046	201,222,026	*	*	*ZmVTE4*	China	
	qα–Toc-22	5	A/G-51,045	201,222,043	*	*	*ZmVTE4*	China	
	qα–Toc-23	5	A/G-50,039	201,276,495	*	*	*ZmVTE4*	China	
	qα–Toc-24	5	A/G-25,857	202,616,083	*	*	*ZmVTE4*	China	
**δ–Toc**	*qδ–Toc-1*	2	A/G-10,638	86,253,631	*	*	*	China	
	qδ–Toc-2	3	A/G-48,923	225,227,814	*	*	*	China	
	qδ–Toc-3	4	A/G-18,695	61,876,947	*	*	*	China	
	qδ–Toc-4	5	A/G-24,089	103,306,223	*	*	*	China	
	qδ–Toc-5	9	A/G2,725	94,285,743	*	*	*	China	
**γ–Toc**	*qγ–Toc-1*	1	T/C-7,874	227,897,687	*	*	*	China	

	qγ–Toc-2	3	T/G-51,195	213,191,036	*	*	*	China	
	qγ–Toc-3	4	A/G-18,241	34,181,986	*	*	*	China	
**T-Toc**	*qT-Toc-1*	1	T/C-7,874	227,897,687	*	*	*	China	
	qT-Toc-2	2	A/G-11,758	143,273,942	*	*	*	China	
	qT-Toc-3	3	T/G-51,195	213,191,036	*	*	*	China	
	qT-Toc-4	4	A/G-18,241	34,181,986	*	*	*	China	
	qT-Toc-5	5	A/G-25,291	171,214,128	*	*	*	China	
	qT-Toc-6	8	A/G-32,502	22,962,134	*	*	*	China	
**QTL Identified in K22 by C17 RIL population (*n*=237)**									(Shutu et al., 2012)
**α–Toc**	*qc1-1*	1	PHM2130.29-PHM1950.71	97.59-104.09	4.0	3.9	*	China	
	qc5-2	5	PZA01327.1-PZB00869.4	62.04-74.57	5.3	6.2	*	China	
	qc5-1	5	PZA00352.23-PZA02060.1	152.75-166.18	24.0	29.6	*VTE4*	China	
	qc6-2	6	PZA02262.3-PZB01308.2	87.7-95.2	4.7	4.7	*	China	
	qc5-2	5	PZA03226.3-PZA02207.1	67.14-79.77	4.1	6.1	*	China	
	qc5-1	5	PZA00352.23-PZA02060.1	152.75-166.18	12.3	20.1	*VTE4*	China	
	qc1-1	1	PZA00081.18-PZA03189.4	85.05-101.69	4.1	5.5	*	China	
	qc5-2	5	PZA01327.1-PHM16854.3	62.04-77.24	14.6	18.2	*	China	
	qc5-1	5	PZA00352.23-PZA02060.1	152.75-166.18	7.9	9.8	*VTE4*	China	
**γ–Toc**	*qc2-1*	2	PZB00901.3-PZA03228.4	24.02-54.47	4.3	6.0	*	China	
	qc5-2	5	PZA01327.1-PHM16854.3	62.04-77.24	11.8	17.6	*	China	
	qc5-3	5	PHM3612.19-PHM13639.13	196.03-207.23	4.0	5.2	*	China	
	qc7-1	7	PZA03149.4-PZA02643.1	48.86-62.83	6.1	8.8	*	China	
	qc5-4	5	PZA01371.1-PZA01327.1	43.04-62.04	5.0	11.0	*	China	
	qc5-5	5	PZA00067.10-PZA00148.3	109.1-115.8	10.2	17.3	*	China	
	qc5-2	5	PZA01327.1-PHM16854.3	62.04-77.24	15.8	25.0	*	China	
	qc5-1	5	PZA03161.1-PZA00545.26	144.6-177.68	5.5	7.8	*TE4*	China	
	qc6-1	6	PHM15961.13-PZA03069.8	7.0-25.9	3.8	5.3	*	China	
**T-Toc**	*qc1-1*	1	PZA00081.18-PHM1932.51	85.05-118.11	5.3	7.1	*	China	
	qc2-2	2	PHM12952.13-PZB00901.4	12.9-25.32	4.1	6.0	*	China	
	qc5-4	5	PZA01371.1-PZA01327.1	43.04-62.04	6.5	9.5	*	China	
	qc5-2	5	PZA02113.1-PHM13675.17	72.97-90.34	12.5	16.9	*	China	
	qc5-1	5	PZA00352.23-PZA02060.1	152.75-166.18	4.3	5.4	*VTE4*	China	
	qc7-1	7	PZA03149.4-PZA02643.1	48.86-62.83	5.3	6.9	*	China	
	qc5-2	5	PZA01327.1-PHM13675.17	62.04-90.34	14.9	26.5	*	China	
	qc5-2	5	PZA01327.1-PZA02207.1	62.04-79.77	11.2	15.5	*	China	
**(α/γ)–Toc Ratio**	*qc5-2*	5	PZA01327.1-PHM2769.43	62.04-83.27	6.8	9.2	*	China	
	qc6-2	6	PZA01729.1-PZA02328.5	60.5-76.8	4.9	8.7	*	China	
	qc8-1	8	PHM934.19-PZA02011.1	69.68-82.39	4.2	5.5	*	China	
	qc5-2	5	PZA03298.1-PHM16854.3	68.87-77.24	5.8	10.7	*	China	
	qc5-1	5	PZA02751.1-PZA02513.1	151.05-165.68	9.1	18.4	*VTE4*	China	
	qc5-2	5	PZA01327.1-PHM2769.43	62.04-83.27	10.5	12.7	*	China	
	qc5-1	5	PZA00352.23-PZA02015.11	152.75-180.48	19.3	26.0	*VTE4*	China	
	qc6-2	6	PZA00473.5-PZA02262.3	60.4-87.7	6.1	8.3	*	China	
**QTL Identified in K22 by Dan340 RIL population (*n*=218)**									
**α–Toc**	*qd5-1*	5	PZA02751.1-PZA02068.1	151.05-169.78	21.9	52.8	*VTE4*	China	
	qd8-1	8	PZB00592.1-PHM4203.11	76.29-77.22	3.7	5.0		China	
	qd5-1	5	PZA02751.1-PZA02068.1	151.05-169.78	5.6	12.4	*VTE4*	China	
	qd1-1	1	PHM3690.23-kip1.3	158.46-190.8	7.3	11.1		China	
	qd5-1	5	PZA03161.1-PZA02068.1	144.6-169.78	21.8	39.1	*VTE4*	China	
	qd10-1	10	PZA01642.1-PZA00079.1	28.06-29.45	4.1	5.4		China	
**γ–Toc**	*qd1-2*	1	PZA02750.3-PHM2187.34	119.28-127.7	4.5	3.0		China	

	qd1-1	1	PZA02117.1-PHM4926.16	165.46-179.76	35.0	30.8		China	
	qd5-1	5	PZA02751.1-PZA02068.1	151.05-169.78	15.1	12.1	*VTE4*	China	
	qd8-1	8	PZA02748.3-PZA02011.1	68.92-82.39	6.5	4.4		China	
	qd1-1	1	PHM3690.23-PZB01647.1	158.46-174.76	13.1	23.5		China	
	qd1-2	1	PZA00887.1-PZA00358.12	38.74-49.57	5.3	5.4		China	
	qd1-1	1	PHM3690.23-kip1.3	158.46-190.8	24.3	32.6		China	
	qd2-1	2	PZA03228.4-PHM10404.8	54.47-70.52	3.8	4.1		China	
	qd5-1	5	PZA03161.1-PZA01265.1	144.6-163.15	14.4	18.2	*VTE4*	China	
**T-Toc**	*qd1-3*	1	PZA02292.1-PZA01267.3	89.29-109.03	4.9	4.0		China	
	qd1-1	1	PHM3690.23-PZB01647.1	158.46-174.76	42.1	50.7		China	
	qd5-2	5	PZA01779.1-PZA00643.13	103.8-107.1	6.2	5.7	*HPPD-5*	China	
	qd5-1	5	PZA03161.1-PZA01265.1	144.6-163.15	7.5	6.2	*VTE4*	China	
	qd1-1	1	PHM3690.23-PZB01647.1	158.46-174.76	13.4	25.3		China	
	qd1-4	1	PZA00887.1-PZA00358.12	38.74-49.57	4.7	5.6		China	
	qd1-1	1	PHM3690.23-PZB01647.1	158.46-174.76	24.1	42.5		China	
	qd2-1	2	PZA03228.4-PHM10404.8	54.47-70.52	3.7	4.7		China	
**(α/γ)–Toc Ratio**	*qd1-1*	1	PHM3690.23-PHM4926.16	158.46-179.76	24.2	26.7		China	
	qd5-1	5	PZA02751.1-PZA02068.1	151.05-169.78	24.3	28.2	*VTE4*	China	
	qd8-1	8	PZB00592.1-LYCE.1	76.29-79.82	4.3	4.3		China	
	qd1-1	1	PHM3690.23-PZB01647.1	158.46-174.76	9.8	17.9		China	
	qd5-1	5	PZA02751.1-PZA02068.1	151.05-169.78	5.5	10.6	*VTE4*	China	
	qd1-1	1	PHM3690.23-PZB01647.1	158.46-174.76	3.6	4.7		China	
	qd5-1	5	PZA03161.1-PZA02068.1	144.6-169.78	26.8	51.0	*VTE4*	China	
**α-Toc**	*qα-Toc-1*	1	umc1177–bnlg1429	0–2	4.7	20.8	*	China	(Feng et al., 2013)
	qα-Toc-2	2	phi96100–umc1265	0–4	2.8	5.3	*	China	
**δ–Toc**	*qδ–Toc-1*	10	umc2180–umc2163	145–148	2.6	4.7	*	China	
**γ–Toc**	*qγ–Toc-1*	1	umc1177–bnlg1429	0–7	11.4	32.1	*	China	
	qγ–Toc-2	5	umc2036–HPPD–umc1692	67–112	7.4	13.7	*	China	
**T-Toc**	*qT-Toc-1*	1	umc1177–bnlg1429	1–6	6.5	24.6	*	China	
	qT-Toc-2	1	bnlg1811–bnlg2086	92–99	2.7	6.2	*	China	
	qT-Toc-3	5	umc2036–HPPD–umc1692	67–113	8.6	15.4	*	China	
	qT-Toc-4	6	bnlg1740–phi364545	272–301	3.1	6.3	*	China	
**(α/γ)–Toc Ratio**	*q(α/γ)–Toc-1*	1	umc1177–bnlg1429	0–1	12.4	41.2	*	China	
	q(α/γ)–Toc-2	2	phi96100–umc1265	0–7	3.1	5.8	*	China	
**αT/γT**	*qαT/γT*	1	S1_16043898	16,043,898	7.25E-06	31.0	*	IN, USA	(Lipka et al., 2013)
**δT/αT**	*qδT/αT*	1	S1_42184756	42,184,756	7.43E-06	34.0	*	IN, USA	
**αT**	*qαT*	1	S1_69880751	69,880,751	8.96E-06	32.0	*	IN, USA	
**αT/γT**	*qαT/γT*	1	S1_207726397	207,726,397	3.89E-06	31.0	*	IN, USA	
**αT/γT**	*qαT/γT*	1	S1_207726398	207,726,398	3.89E-06	31.0	*	IN, USA	
**δT3/(γT3+αT3)**	*qδT3/(γT3+αT3)*	2	S2_71606588	71,606,588	6.14E-06	18.0	*	IN, USA	
**αT**	*qαT*	2	S2_196514167	196,514,167	7.19E-06	32.0	*	IN, USA	
**αT**	*qαT*	2	S2_219396629	219,396,629	8.33E-06	32.0	*	IN, USA	
**αT**	*qαT*	2	S2_219396633	219,396,633	8.33E-06	32.0	*	IN, USA	
**γT/(γT+αT)**	*qγT/(γT+αT)*	3	ss196506839	197,518,747	5.72E-06	35.0	*	IN, USA	
**δT3**	*qδT3*	5	S5_132656905	132,656,905	2.15E-06	20.0	*	IN, USA	
**δT3/(γT3+αT3)**	*qδT3/(γT3+αT3)*	5	S5_133189938	133,189,938	2.29E-06	18.0	*	IN, USA	
**δT3**	*qδT3*	5	S5_133331096	133,331,096	1.01E-06	21.0	*GRMZM2G105494*	IN, USA	
**δT3**	*qδT3*	5	S5_133331106	133,331,106	1.01E-06	21.0	*GRMZM2G105494*	IN, USA	
**δT3**	*qδT3*	5	S5_133333397	133,333,397	3.06E-07	22.0	*GRMZM2G105494*	IN, USA	
**δT3**	*qδT3*	5	ss196465626	133,333,397	1.69E-06	21.0	*GRMZM2G105494*	IN, USA	
**δT3/(γT3+αT3)**	*qδT3/(γT3+αT3)*	5	S5_133333397	133,333,397	6.13E-06	18.0	*GRMZM2G105494*	IN, USA	

**δT3**	*qδT3*	5	S5_133333561	133,333,561	3.12E-07	22.0	*GRMZM2G105494*	IN, USA
**δT3/(γT3+αT3)**	*qδT3/(γT3+αT3)*	5	S5_133333561	133,333,561	8.28E-06	17.0	*GRMZM2G105494*	IN, USA
**δT3**	*qδT3*	5	S5_133335078	133,335,078	1.34E-06	21.0	*GRMZM2G105494*	IN, USA
**δT3/(γT3+αT3)**	*qδT3/(γT3+αT3)*	5	S5_133499169	133,499,169	1.77E-06	19.0	*GRMZM2G009785*	IN, USA
**δT3/(γT3+αT3)**	*qδT3/(γT3+αT3)*	5	S5_133499269	133,499,269	3.66E-06	18.0	*GRMZM2G009785*	IN, USA
**δT3/(γT3+αT3)**	*qδT3/(γT3+αT3)*	5	S5_133501858	133,501,858	1.29E-07	21.0	*GRMZM2G009785*	IN, USA
**δT3/(γT3+αT3)**	*qδT3/(γT3+αT3)*	5	S5_133501992	133,501,992	9.72E-07	19.0	*GRMZM2G009785*	IN, USA
**δT3/(γT3+αT3)**	*qδT3/(γT3+αT3)*	5	PZB00969.1	133,502,506	8.27E-07	19.0	*GRMZM2G009785*	IN, USA
**δT3/(γT3+αT3)**	*qδT3/(γT3+αT3)*	5	ss196465634	133,510,613	7.54E-06	17.0	*GRMZM2G009785*	IN, USA
**δT3/(γT3+αT3)**	*qδT3/(γT3+αT3)*	5	PZB02491.1	133,517,065	6.69E-06	18.0	*GRMZM2G009785*	IN, USA
**δT3/(γT3+αT3)**	*qδT3/(γT3+αT3)*	5	S5_133618308	133,618,308	7.89E-06	17.0	*	IN, USA
**δT3/(γT3+αT3)**	*qδT3/(γT3+αT3)*	5	S5_133618309	133,618,309	7.89E-06	17.0	*	IN, USA
**δT3/(γT3+αT3)**	*qδT3/(γT3+αT3)*	5	S5_133618344	133,618,344	7.89E-06	17.0	*	IN, USA
**δT3/(γT3+αT3)**	*qδT3/(γT3+αT3)*	5	S5_133691297	133,691,297	5.02E-06	18.0	*	IN, USA
**δT3/(γT3+αT3)**	*qδT3/(γT3+αT3)*	5	S5_133776316	133,776,316	6.51E-07	19.0	*	IN, USA
**δT3/(γT3+αT3)**	*qδT3/(γT3+αT3)*	5	S5_134191258	134,191,258	1.66E-06	19.0	*	IN, USA
**δT3/(γT3+αT3)**	*qδT3/(γT3+αT3)*	5	S5_134195301	134,195,301	2.92E-06	18.0	*	IN, USA
**δT3/(γT3+αT3)**	*qδT3/(γT3+αT3)*	5	S5_134471200	134,471,200	1.13E-06	19.0	*	IN, USA
**δT3/(γT3+αT3)**	*qδT3/(γT3+αT3)*	5	S5_134489952	134,489,952	1.13E-06	19.0	*	IN, USA
**δT3/(γT3+αT3)**	*qδT3/(γT3+αT3)*	5	S5_134751850	134,751,850	2.34E-06	18.0	*	IN, USA
**δT3/(γT3+αT3)**	*qδT3/(γT3+αT3)*	5	S5_134751883	134,751,883	7.69E-06	17.0	*	IN, USA
**δT3/(γT3+αT3)**	*qδT3/(γT3+αT3)*	5	S5_134751924	134,751,924	6.83E-06	18.0	*	IN, USA
**δT3/(γT3+αT3)**	*qδT3/(γT3+αT3)*	5	S5_134787527	134,787,527	6.62E-06	18.0	*	IN, USA
**δT3/(γT3+αT3)**	*qδT3/(γT3+αT3)*	5	S5_135908766	135,908,766	8.47E-06	17.0	*	IN, USA
**δT3/(γT3+αT3)**	*qδT3/(γT3+αT3)*	5	S5_135908783	135,908,783	8.47E-06	17.0	*	IN, USA
**γT/(γT+αT)**	*qγT/(γT+αT)*	5	S5_190739882	190,739,882	1.15E-06	36.0	*GRMZM2G031952*	IN, USA
**αT/γT**	*qαT/γT*	5	S5_190739882	190,739,882	7.45E-06	31.0	*GRMZM2G031952*	IN, USA
**γT/(γT+αT)**	*qγT/(γT+αT)*	5	S5_190739951	190,739,951	9.59E-08	37.0	*GRMZM2G031952*	IN, USA
**αT/γT**	*qαT/γT*	5	S5_190739951	190,739,951	5.40E-07	33.0	*GRMZM2G031952*	IN, USA
**αT**	*qαT*	5	S5_198205644	198,205,644	3.56E-06	32.0	*GRMZM2G058943*	IN, USA
**αT**	*qαT*	5	S5_199647093	199,647,093	3.54E-06	32.0	*GRMZM5G823157*	IN, USA
**δT/αT**	*qδT/αT*	5	S5_199647093	199,647,093	4.26E-06	34.0	*GRMZM5G823157*	IN, USA
**αT/γT**	*qαT/γT*	5	S5_199706178	199,706,178	1.07E-06	32.0	*	IN, USA
**γT/(γT+αT)**	*qγT/(γT+αT)*	5	S5_199706178	199,706,178	1.22E-06	36.0	*	IN, USA
**δT/αT**	*qδT/αT*	5	S5_199706178	199,706,178	1.68E-06	35.0	*	IN, USA
**αT/γT**	*qαT/γT*	5	S5_199706244	199,706,244	8.86E-06	31.0	*	IN, USA
**αT**	*qαT*	5	S5_200114137	200,114,137	2.63E-07	34.0	*GRMZM2G108996*	IN, USA
**γT/(γT+αT)**	*qγT/(γT+αT)*	5	S5_200293693	200,293,693	1.24E-06	36.0	*GRMZM2G034876*	IN, USA
**αT**	*qαT*	5	S5_200293693	200,293,693	3.33E-06	32.0	*GRMZM2G034876*	IN, USA
**αT/γT**	*qαT/γT*	5	S5_200293693	200,293,693	8.10E-06	31.0	*GRMZM2G034876*	IN, USA
**δT/αT**	*qδT/αT*	5	ss196468356	200,300,836	6.33E-06	34.0	*	IN, USA
**αT**	*qαT*	5	S5_200318615	200,318,615	1.05E-06	33.0	*	IN, USA
**αT/γT**	*qαT/γT*	5	S5_200318615	200,318,615	2.50E-06	32.0	*	IN, USA
**αT**	*qαT*	5	ss196416269	200,367,532	7.36E-14	44.0	*GRMZM2G035213*	IN, USA
**αT**	*qαT*	5	PZB02283.1	200,367,532	7.36E-14	44.0	*GRMZM2G035213*	IN, USA
**αT/γT**	*qαT/γT*	5	PZB02283.1	200,367,532	9.21E-13	42.0	*GRMZM2G035213*	IN, USA
**αT/γT**	*qαT/γT*	5	ss196416269	200,367,532	9.21E-13	42.0	*GRMZM2G035213*	IN, USA
**γT/(γT+αT)**	*qγT/(γT+αT)*	5	PZB02283.1	200,367,532	4.82E-12	44.0	*GRMZM2G035213*	IN, USA
**γT/(γT+αT)**	*qγT/(γT+αT)*	5	ss196416269	200,367,532	4.82E-12	44.0	*GRMZM2G035213*	IN, USA
**δT/αT**	*qδT/αT*	5	PZB02283.1	200,367,532	1.88E-11	42.0	*GRMZM2G035213*	IN, USA
**δT/αT**	*qδT/αT*	5	ss196416269	200,367,532	1.88E-11	42.0	*GRMZM2G035213*	IN, USA
**αT**	*qαT*	5	ss196468362	200,369,124	7.36E-14	44.0	*GRMZM2G035213*	IN, USA

**αT/γT**	*qαT/γT*	5	ss196468362	200,369,124	9.21E-13	42.0	*GRMZM2G035213*	IN, USA
**γT/(γT+αT)**	*qγT/(γT+αT)*	5	ss196468362	200,369,124	4.82E-12	44.0	*GRMZM2G035213*	IN, USA
**δT/αT**	*qδT/αT*	5	ss196468362	200,369,124	1.88E-11	42.0	*GRMZM2G035213*	IN, USA
**αT**	*qαT*	5	S5_200369481	200,369,481	1.97E-08	35.0	*GRMZM2G035213*	IN, USA
**αT/γT**	*qαT/γT*	5	S5_200369481	200,369,481	7.24E-07	32.0	*GRMZM2G035213*	IN, USA
**γT/(γT+αT)**	*qγT/(γT+αT)*	5	S5_200369481	200,369,481	6.15E-09	39.0	*GRMZM2G035213*	IN, USA
**δT/αT**	*qδT/αT*	5	S5_200369481	200,369,481	7.21E-07	35.0	*GRMZM2G035213*	IN, USA
**αT**	*qαT*	5	S5_200369508	200,369,508	6.85E-07	33.0	*GRMZM2G035213*	IN, USA
**αT/γT**	*qαT/γT*	5	S5_200369508	200,369,508	9.69E-07	32.0	*GRMZM2G035213*	IN, USA
**γT/(γT+αT)**	*qγT/(γT+αT)*	5	S5_200369508	200,369,508	6.57E-07	36.0	*GRMZM2G035213*	IN, USA
**αT**	*qαT*	5	S5_200369534	200,369,534	2.59E-06	32.0	*GRMZM2G035213*	IN, USA
**αT/γT**	*qαT/γT*	5	S5_200369534	200,369,534	5.45E-06	31.0	*GRMZM2G035213*	IN, USA
**αT**	*qαT*	5	S5_200369625	200,369,625	5.00E-08	35.0	*GRMZM2G035213*	IN, USA
**δT/αT**	*qδT/αT*	5	S5_200369625	200,369,625	2.02E-06	35.0	*GRMZM2G035213*	IN, USA
**αT**	*qαT*	5	S5_200369644	200,369,644	9.36E-06	32.0	*GRMZM2G035213*	IN, USA
**αT/γT**	*qαT/γT*	5	S5_200369644	200,369,644	9.97E-06	31.0	*GRMZM2G035213*	IN, USA
**αT**	*qαT*	5	S5_200369665	200,369,665	9.36E-06	32.0	*GRMZM2G035213*	IN, USA
**αT/γT**	*qαT/γT*	5	S5_200369665	200,369,665	9.97E-06	31.0	*GRMZM2G035213*	IN, USA
**αT**	*qαT*	5	S5_200369667	200,369,667	1.67E-07	34.0	*GRMZM2G035213*	IN, USA
**αT/γT**	*qαT/γT*	5	S5_200369667	200,369,667	1.70E-07	33.0	*GRMZM2G035213*	IN, USA
**δT/αT**	*qδT/αT*	5	S5_200369667	200,369,667	7.09E-06	34.0	*GRMZM2G035213*	IN, USA
**αT**	*qαT*	5	PZB02424.2	200,370,309	4.09E-07	33.0	*GRMZM2G035213*	IN, USA
**αT**	*qαT*	5	ss196468368	200,371,057	6.73E-06	32.0	*GRMZM2G035213*	IN, USA
**αT/γT**	*qαT/γT*	5	ss196468368	200,371,057	7.37E-06	31.0	*GRMZM2G035213*	IN, USA
**γT/(γT+αT)**	*qγT/(γT+αT)*	5	S5_200382168	200,382,168	9.59E-09	39.0	*GRMZM2G167431*	IN, USA
**αT**	*qαT*	5	S5_200382168	200,382,168	3.01E-08	35.0	*GRMZM2G167431*	IN, USA
**αT/γT**	*qαT/γT*	5	S5_200382168	200,382,168	3.08E-08	34.0	*GRMZM2G167431*	IN, USA
**δT/αT**	*qδT/αT*	5	S5_200382168	200,382,168	6.31E-08	37.0	*GRMZM2G167431*	IN, USA
**γT/(γT+αT)**	*qγT/(γT+αT)*	5	S5_200435108	200,435,108	1.13E-10	42.0	*GRMZM2G325001*	IN, USA
**αT/γT**	*qαT/γT*	5	S5_200435108	200,435,108	3.28E-10	38.0	*GRMZM2G325001*	IN, USA
**αT**	*qαT*	5	S5_200435108	200,435,108	4.39E-10	38.0	*GRMZM2G325001*	IN, USA
**δT/αT**	*qδT/αT*	5	S5_200435108	200,435,108	6.37E-09	39.0	*GRMZM2G325001*	IN, USA
**αT**	*qαT*	5	S5_200435117	200,435,117	1.21E-07	34.0	*GRMZM2G325001*	IN, USA
**γT/(γT+αT)**	*qγT/(γT+αT)*	5	S5_200435117	200,435,117	1.66E-07	37.0	*GRMZM2G325001*	IN, USA
**αT/γT**	*qαT/γT*	5	S5_200435117	200,435,117	1.79E-07	33.0	*GRMZM2G325001*	IN, USA
**δT/αT**	*qδT/αT*	5	S5_200435117	200,435,117	7.93E-07	35.0	*GRMZM2G325001*	IN, USA
**αT**	*qαT*	5	ss196468352	200,435,300	8.32E-10	38.0	*GRMZM2G325019*	IN, USA
**γT/(γT+αT)**	*qγT/(γT+αT)*	5	ss196468352	200,435,300	7.67E-09	39.0	*GRMZM2G325019*	IN, USA
**αT/γT**	*qαT/γT*	5	ss196468352	200,435,300	2.68E-08	35.0	*GRMZM2G325019*	IN, USA
**δT/αT**	*qδT/αT*	5	ss196468352	200,435,300	2.87E-08	38.0	*GRMZM2G325019*	IN, USA
**αT**	*qαT*	5	S5_200437468	200,437,468	2.29E-08	35.0	*GRMZM2G325019*	IN, USA
**γT/(γT+αT)**	*qγT/(γT+αT)*	5	S5_200437468	200,437,468	4.13E-08	38.0	*GRMZM2G325019*	IN, USA
**αT/γT**	*qαT/γT*	5	S5_200437468	200,437,468	9.02E-08	34.0	*GRMZM2G325019*	IN, USA
**δT/αT**	*qδT/αT*	5	S5_200437468	200,437,468	3.72E-07	36.0	*GRMZM2G325019*	IN, USA
**αT/γT**	*qαT/γT*	5	ss196517251	200,437,606	4.45E-07	33.0	*GRMZM2G325019*	IN, USA
**αT**	*qαT*	5	ss196517251	200,437,606	1.38E-06	33.0	*GRMZM2G325019*	IN, USA
**γT/(γT+αT)**	*qγT/(γT+αT)*	5	ss196517251	200,437,606	2.99E-06	35.0	*GRMZM2G325019*	IN, USA
**δT/αT**	*qδT/αT*	5	ss196517251	200,437,606	5.53E-06	34.0	*GRMZM2G325019*	IN, USA
**αT**	*qαT*	5	S5_200438801	200,438,801	4.30E-06	32.0	*GRMZM2G325038*	IN, USA
**αT/γT**	*qαT/γT*	5	S5_200438801	200,438,801	7.67E-06	31.0	*GRMZM2G325038*	IN, USA
**αT/γT**	*qαT/γT*	5	S5_204064933	204,064,933	3.15E-08	34.0	*GRMZM2G161641*	IN, USA
**γT/(γT+αT)**	*qγT/(γT+αT)*	5	S5_204064933	204,064,933	3.32E-07	36.0	*GRMZM2G161641*	IN, USA

**αT**	*qαT*	5	S5_204064933	204,064,933	1.16E-06	33.0	*GRMZM2G161641*	IN, USA	
**δT/αT**	*qδT/αT*	5	S5_204064933	204,064,933	8.84E-07	35.0	*GRMZM2G161641*	IN, USA	
**αT/γT**	*qαT/γT*	5	S5_204065009	204,065,009	3.42E-07	33.0	*GRMZM2G161641*	IN, USA	
**δT/αT**	*qδT/αT*	5	S5_204065009	204,065,009	3.87E-07	36.0	*GRMZM2G161641*	IN, USA	
**γT/(γT+αT)**	*qγT/(γT+αT)*	5	S5_204065009	204,065,009	1.74E-06	35.0	*GRMZM2G161641*	IN, USA	
**αT**	*qαT*	5	S5_204065009	204,065,009	4.64E-06	32.0	*GRMZM2G161641*	IN, USA	
**δT/αT**	*qδT/αT*	5	S5_204065055	204,065,055	4.68E-08	37.0	*GRMZM2G161641*	IN, USA	
**αT/γT**	*qαT/γT*	5	S5_204065055	204,065,055	2.36E-06	32.0	*GRMZM2G161641*	IN, USA	
**αT/γT**	*qαT/γT*	6	S6_149776596	149,776,596	9.71E-07	32.0	*	IN, USA	
**αT**	*qαT*	6	S6_149776596	149,776,596	6.87E-06	32.0	*	IN, USA	
**αT/γT**	*qαT/γT*	6	S6_149776597	149,776,597	4.37E-06	31.0	*	IN, USA	
**αT**	*qαT*	6	S6_149776597	149,776,597	1.03E-05	31.0	*	IN, USA	
**αT/γT**	*qαT/γT*	7	ss196475603	13,959,219	1.25E-05	31.0	*	IN, USA	
**αT/γT**	*qαT/γT*	8	ss196486649	128,548,850	7.50E-06	31.0	*	IN, USA	
**γT3**	*qγT3*	9	S9_92718671	92,718,671	4.48E-07	28.0	*GRMZM5G833760*	IN, USA	
**γT3**	*qγT3*	9	S9_92718674	92,718,674	4.48E-07	28.0	*GRMZM5G833760*	IN, USA	
**γT3**	*γT3*	9	S9_92718709	92,718,709	4.48E-07	28.0	*GRMZM5G833760*	IN, USA	
**Total Tocotrienols**	*qT-Tocotrienols*	9	S9_107839821	107,839,821	2.00E-07	30.0	*	IN, USA	
**αT/γT**	*qαT/γT*	9	S9_151726463	151,726,463	4.81E-06	31.0	*	IN, USA	
**αT/γT**	*qαT/γT*	9	S9_151726511	151,726,511	1.07E-05	31.0	*	IN, USA	
**αT/γT**	*qαT/γT*	9	S9_151726870	151,726,870	9.55E-06	31.0	*	IN, USA	
**αT/γT**	*qαT/γT*	9	S9_155585508	155,585,508	2.62E-06	32.0	*	IN, USA	
**δT/αT**	*qδT/αT*	9	S9_155585508	155,585,508	2.75E-06	35.0	*	IN, USA	
**δT/αT**	*qδT/αT*	10	S10_129705301	129,705,301	6.39E-06	34.0	*	IN, USA	
**δT/αT**	*qδT/αT*	10	S10_129705304	129,705,304	6.39E-06	34.0	*	IN, USA	
**αT**	*qαT-1*	1	S_30027714	48.9	1.21	29.53	Chorismate synthase	IN, USA	(Diepenbrock et al., 2017)
	qαT-2	1	S_230132209	136.3	0.01	27.18	*	IN, USA	
	qαT-3	2	S_210456683	116.3	0.01	26.32	*	IN, USA	
	qαT-4	3	S_160847275	72.4	0.01	24.09	*	IN, USA	
	qαT-5	3	S_188410390	98	0.01	22.92	4-diphosphocytidyl-2-C-methyl-D-erythritol kinase, chorismate mutase	IN, USA	
	qαT-6	4	S_13319985	35.2	0.02	39.60	*	IN, USA	
	qαT-7	4	S_171741420	79	0.01	30.45	*	IN, USA	
	qαT-8	5	S_24668030	53.9	0.01	25.70	*	IN, USA	
	qαT-9	5	S_200356718	106.8	0.48	755.11	Gamma-tocopherol methyltransferase (VTE4)	IN, USA	
	qαT-10	6	S_140347572	54.4	0.01	32.73	*	IN, USA	
	qαT-11	7	S_140150735	73	0.02	44.64	*	IN, USA	
	qαT-12	8	S_141756913	71.6	0.02	50.62	*	IN, USA	
	qαT-13	9	S_27141915	43.6	0.02	40.57	Arogenate/prephenate dehydrogenase family protein, Homogentisate 1,2-dioxygenase	IN, USA	
**δT**	*qδT-1*	1	S_32200366	51.2	0.01	22.23	Chorismate synthase	IN, USA	
	qδT-2	1	S_174102804	94.3	0.08	136.26	MPBQ/MSBQ methyl transferase (VTE3)	IN, USA	
	qδT-3	1	S_227227502	133.8	0.06	99.97	*	IN, USA	
	qδT-4	2	S_37143380	65.7	0.01	24.02	*	IN, USA	
	qδT-5	2	S_210003103	115.7	0.04	78.35	*	IN, USA	
	qδT-6	3	S_173244742	82.1	0.01	22.78	Prephenate amino transferase	IN, USA	

	qδT-7	4	S_26785112	48.4	0.01	22.44		IN, USA
	qδT-8	5	S_25536964	54.2	0.14	167.62		IN, USA
	qδT-9	5	S_133824940	70.9	0.05	55.83	Tocopherol cyclase (VTE1)	IN, USA
	qδT-10	5	S_196287168	103.8	0.02	22.55	2-C-methyl-D-erythritol 2,4-cyclodiphosphate synthase	IN, USA
	qδT-11	5	S_204809285	114	0.03	34.17	*	IN, USA
	qδT-12	6	S_108619629	32.1	0.02	32.38	*	IN, USA
	qδT-13	6	S_159984809	83.9	0.01	19.62	*	IN, USA
	qδT-14	7	S_34185670	48.1	0.02	45.62	Chlorophyllase, 4-hydroxyphenylpyruvate dioxygenase	IN, USA
	qδT-15	9	S_31124337	44.6	0.04	62.90	homogentisate 1,2-dioxygenase	IN, USA
	qδT-16	9	S_149164320	93.4	0.02	33.30	*	IN, USA
	qδT-17	10	S_78533820	38.7	0.01	22.17	*	IN, USA
	qδT-18	10	S_144982565	83.1	0.02	42.59	*	IN, USA
**γT**	*qγT-1*	1	S_12293829	24.4	0.01	19.24	Solanesyl diphosphate synthase	IN, USA
	qγT-2	1	S_32578218	51.6	0.02	34.62	Chorismate synthase	IN, USA
	qγT-3	1	S_227227502	133.8	0.11	210.16	*	IN, USA
	qγT-4	2	S_32255033	62.8	0.01	28.09	*	IN, USA
	qγT-5	2	S_195781723	103.9	0.02	22.98	3-dehydroquinate synthase	IN, USA
	qγT-6	2	S_209749910	115.5	0.08	95.76		IN, USA
	qγT-7	3	S_25884552	52.5	0.01	21.59		IN, USA
	qγT-8	3	S_199624131	104.9	0.01	18.78	Chorismate mutase	IN, USA
	qγT-9	4	S_27272892	48.6	0.02	36.57	*	IN, USA
	qγT-10	5	S_25247319	54.1	0.18	####	*	IN, USA
	qγT-11	5	S_90566175	69.5	0.01	19.09	Chorismate mutase, Tyrosine transaminase, 4-hydroxyphenylpyruvate dioxygenase, Tocopherol cyclase (VTE1)	IN, USA
	qγT-12	5	S_200161433	106.7	0.03	54.04	Gamma-tocopherol methyltransferase (VTE4)	IN, USA
	qγT-13	6	S_75052175	10.3	0.01	20.75	Arogenate/prephenate dehydrogenase family protein	IN, USA
	qγT-14	6	S_118012160	43.2	0.02	34.36	*	IN, USA
	qγT-15	6	S_157048924	76.4	0.01	22.27	*	IN, USA
	qγT-16	7	S_129999310	64.4	0.01	22.35	*	IN, USA
	qγT-17	8	S_136940374	70	0.01	31.19	*	IN, USA
	qγT-18	9	S_94334531	47.4	0.03	46.94	Homogentisic acid geranylgeranyl transferase 1 (HGGT1)	IN, USA
	qγT-19	9	S_138294095	70	0.01	21.21	Arogenate/prephenate dehydratase	IN, USA
	qγT-20	10	S_24852570	35.4	0.02	38.14	Shikimate biosynthesis protein aroDE, maleylacetoacetate isomerase	IN, USA
	qγT-21	10	S_144982565	83.1	0.02	37.56	*	IN, USA
**TotalT**	*qTotalT-1*	1	S_33258826	52.5	0.02	35.59	Chorismate synthase	IN, USA
	qTotalT-2	1	S_227227502	133.8	0.10	175.70	*	IN, USA
	qTotalT-3	2	S_22420256	53.6	0.01	23.98	*	IN, USA
	qTotalT-4	2	S_195781723	103.9	0.02	20.02	3-dehydroquinate synthase	IN, USA
	qTotalT-5	2	S_209749910	115.5	0.07	78.34	*	IN, USA

	qTotalT-6	3	S_24965860	52.2	0.01	23.49	*	IN, USA
	qTotalT-7	3	S_190652083	99.4	0.01	21.57	Chorismate mutase	IN, USA
	qTotalT-8	4	S_29529943	49.5	0.02	34.82	*	IN, USA
	qTotalT-9	5	S_25247319	54.1	0.18	####	*	IN, USA
	qTotalT-10	5	S_145388055	72.3	0.01	21.61	Chorismate mutase, tocopherol cyclase (VTE1)	IN, USA
	qTotalT-11	6	S_75052175	10.3	0.01	18.03	Arogenate/prephenate dehydrogenase family protein	IN, USA
	qTotalT-12	6	S_117936528	43.1	0.02	30.59	*	IN, USA
	qTotalT-13	6	S_157048924	76.4	0.01	19.25	*	IN, USA
	qTotalT-14	8	S_143642609	72.8	0.02	34.52	*	IN, USA
	qTotalT-15	9	S_94334531	47.4	0.03	49.65	Homogentisic acid geranylgeranyl transferase 1 (HGGT1)	IN, USA
	qTotalT-16	9	S_138294095	70	0.01	22.48	Arogenate/prephenate dehydratase	IN, USA
	qTotalT-17	10	S_24852570	35.4	0.02	32.71	Shikimate biosynthesis protein aroDE, maleylacetoacetate isomerase	IN, USA
	qTotalT-18	10	S_144982565	83.1	0.02	33.65	*	IN, USA
**αT3**	*qαT3-1*	1	S_37464200	55.7	0.01	25.24	*	IN, USA
	qαT3-2	1	S_259191668	155.1	0.02	27.53	*	IN, USA
	qαT3-3	1	S_280349239	172.7	0.01	23.22	*	IN, USA
	qαT3-4	2	S_39280552	67.1	0.01	21.67	*	IN, USA
	qαT3-5	3	S_143898953	63.7	0.03	59.29	*	IN, USA
	qαT3-6	3	S_229858254	153.8	0.01	30.65	*	IN, USA
	qαT3-7	4	S_75010949	56.6	0.02	51.01	Chorismate mutase	IN, USA
	qαT3-8	5	S_73132746	65.9	0.02	30.98	Light harvesting-like protein 3, arogenate/prephenate dehydrogenase family protein, arogenate/prephenate dehydrogenase family protein	IN, USA
	qαT3-9	5	S_200356718	106.8	0.32	*	Gamma-tocopherol methyltransferase (VTE4)	IN, USA
	qαT3-10	6	S_108288402	31.9	0.01	20.52	*	IN, USA
	qαT3-11	6	S_161454721	86.6	0.01	21.68	*	IN, USA
	qαT3-12	7	S_84844392	49.3	0.03	66.87	*	IN, USA
	qαT3-13	7	S_171757675	121.1	0.01	26.28	*	IN, USA
	qαT3-14	8	S_173864256	129.7	0.01	20.60	*	IN, USA
	qαT3-15	9	S_87459733	46.3	0.03	65.31	*	IN, USA
	qαT3-16	10	S_9441157	24.8	0.02	29.26	*	IN, USA
	qαT3-17	10	S_113911262	45.4	0.01	22.83	Arogenate/prephenate dehydratase	IN, USA
**δT3**	*qδT3-1*	1	S_5804545	10.2	0.02	41.07	*	IN, USA
	qδT3-2	1	S_16005600	32.0	0.01	21.51	*	IN, USA
	qδT3-3	1	S_174102804	94.3	0.02	49.72	MPBQ/MSBQ methyl transferase (VTE3)	IN, USA
	qδT3-4	1	S_227978512	134.4	0.01	27.05	*	IN, USA
	qδT3-5	1	S_289692611	185.1	0.01	19.76	*	IN, USA
	qδT3-6	3	S_58294211	57.6	0.01	21.73	1-deoxy-D-xylulose 5-phosphate reductoisomerase, geranylgeranyl hydrogenase, shikimate biosynthesis protein aroDE	IN, USA
	qδT3-7	3	S_201306180	106.7	0.01	18.79	*	IN, USA
	qδT3-8	4	S_86504397	57.6	0.01	32.72	Chorismate mutase	IN, USA
	qδT3-9	5	S_1491318	1.7	0.01	35.45	*	IN, USA

	qδT3-10	5	S_18348509	48.9	0.01	19.19	*	IN, USA
	qδT3-11	5	S_83712444	68.5	0.11	182.20	4-hydroxyphenylpyruvate dioxygenase	IN, USA
	qδT3-12	5	S_204809285	114.0	0.03	71.91	*	IN, USA
	qδT3-13	6	S_72953671	10.1	0.03	60.38	*	IN, USA
	qδT3-14	6	S_111902384	36.5	0.01	26.36	*	IN, USA
	qδT3-15	6	S_160013836	84.0	0.01	21.96	*	IN, USA
	qδT3-16	7	S_13934903	40.5	0.06	65.80	1-deoxy-D-xylulose 5-phosphate synthase 2 (dxs2)	IN, USA
	qδT3-17	7	S_31773777	48.0	0.07	72.38	*	IN, USA
	qδT3-18	8	S_144742598	73.5	0.01	20.71	*	IN, USA
	qδT3-19	8	S_173232589	128.0	0.01	24.20	Chorismate mutase, chorismate mutase	IN, USA
	qδT3-20	9	S_92175763	47.0	0.24	####	Homogentisic acid geranylgeranyl transferase 1 (HGGT1)	IN, USA
	qδT3-21	10	S_16505881	33.3	0.01	28.96	*	IN, USA
**γT3**	*qγT3-1*	1	S_77561162	79.9	0.02	52.52	*	IN, USA
	qγT3-2	1	S_228086777	134.5	0.01	16.61	*	IN, USA
	qγT3-3	2	S_39891173	67.5	0.01	18.26	Arogenate/prephenate dehydratase	IN, USA
	qγT3-4	3	S_122684562	59.5	0.01	19.08	*	IN, USA
	qγT3-5	4	S_40700355	54	0.01	31.41	*	IN, USA
	qγT3-6	5	S_22579093	52.6	0.01	19.40	*	IN, USA
	qγT3-7	5	S_84251695	68.6	0.08	144.13	4-hydroxyphenylpyruvate dioxygenase	IN, USA
	qγT3-8	5	S_200356718	106.8	0.04	100.56	Gamma-tocopherol methyltransferase (VTE4)	IN, USA
	qγT3-9	6	S_82648835	11.5	0.03	64.73	Arogenate/prephenate dehydrogenase family protein	IN, USA
	qγT3-10	6	S_132730267	51.1	0.01	25.92	ABC kinase (tocopherol cyclase kinase)	IN, USA
	qγT3-11	7	S_14043551	40.6	0.05	139.07	1-deoxy-D-xylulose 5-phosphate synthase 2 (dxs2)	IN, USA
	qγT3-12	8	S_173232589	128	0.01	25.49	Chorismate mutase, chorismate mutase	IN, USA
	qγT3-13	9	S_92175763	47	0.40	731.30	Homogentisic acid geranylgeranyl transferase 1 (HGGT1)	IN, USA
	qγT3-14	10	S_14819626	32.4	0.01	27.47	*	IN, USA
**TotalT3**	*qTotalT3-1*	1	S_77561162	79.9	0.01	23.29	*	IN, USA
	qTotalT3-2	2	S_40196483	67.7	0.01	19.61	*	IN, USA
	qTotalT3-3	3	S_144813964	63.8	0.01	20.20	shikimate biosynthesis protein aroDE, shikimate biosynthesis protein aroDE	IN, USA
	qTotalT3-4	4	S_40700355	54	0.01	32.35	*	IN, USA
	qTotalT3-5	5	S_59819753	62.8	0.02	17.36	Arogenate/prephenate dehydrogenase family protein, Arogenate/prephenate dehydrogenase family protein	IN, USA
	qTotalT3-6	5	S_84251695	68.6	0.10	82.46	4-hydroxyphenylpyruvate dioxygenase	IN, USA
	qTotalT3-7	6	S_82648835	11.5	0.03	57.91	Arogenate/prephenate dehydrogenase family protein	IN, USA
	qTotalT3-8	6	S_132730267	51.1	0.01	19.26	1-deoxy-D-xylulose 5-phosphate synthase 1 (dxs1), isopentenyl pyrophosphate isomerase, ABC kinase (tocopherol cyclase kinase)	IN, USA
	qTotalT3-9	7	S_13934903	40.5	0.06	123.32	1-deoxy-D-xylulose 5-phosphate synthase 2 (dxs2)	IN, USA
	qTotalT3-10	8	S_170044134	114.2	0.01	25.70		IN, USA
	qTotalT3-11	9	S_92175763	47	0.39	####	Homogentisic acid geranylgeranyl transferase 1 (HGGT1)	IN, USA

	qTotalT3-12	10	S_108093611	43.9	0.01	28.52	Arogenate/prephenate dehydratase	IN, USA	
**TotalT+T3**	*qTotalT+T3-1*	1	S_32578218	51.6	0.02	26.56	Chorismate synthase	IN, USA	
	qTotalT+T3-2	1	S_173178805	94	0.01	23.62	3-deoxy-d-arabino-heptulosonate 7-phosphate synthase, MPBQ/MSBQ methyl transferase (VTE3)	IN, USA	
	qTotalT+T3-3	1	S_228086777	134.5	0.06	94.22	*	IN, USA	
	qTotalT+T3-4	2	S_185246926	92.1	0.01	18.60	*	IN, USA	
	qTotalT+T3-5	2	S_210003103	115.7	0.04	62.49	*	IN, USA	
	qTotalT+T3-6	3	S_181011607	90.8	0.01	19.65	*	IN, USA	
	qTotalT+T3-7	4	S_41538292	54.1	0.02	42.65	*	IN, USA	
	qTotalT+T3-8	5	S_2437804	5.4	0.01	17.91	*	IN, USA	
	qTotalT+T3-9	5	S_25247319	54.1	0.12	138.56	*	IN, USA	
	qTotalT+T3-10	5	S_84885005	68.7	0.03	41.03	Chorismate mutase, 4-hydroxyphenylpyruvate dioxygenase	IN, USA	
	qTotalT+T3-11	5	S_204322970	111.9	0.01	17.58	*	IN, USA	
	qTotalT+T3-12	6	S_75052175	10.3	0.01	22.55	Arogenate/prephenate dehydrogenase family protein	IN, USA	
	qTotalT+T3-13	6	S_109778922	32.8	0.02	16.99	*	IN, USA	
	qTotalT+T3-14	6	S_127663433	47.2	0.02	20.89	*	IN, USA	
	qTotalT+T3-15	7	S_10636101	35.4	0.02	46.30	1-deoxy-D-xylulose 5-phosphate synthase 2 (dxs2)	IN, USA	
	qTotalT+T3-16	8	S_9998058	25.7	0.01	18.23	*	IN, USA	
	qTotalT+T3-17	8	S_143642609	72.8	0.01	26.14	*	IN, USA	
	qTotalT+T3-18	9	S_94016922	47.3	0.09	153.24	Homogentisic acid geranylgeranyl transferase 1 (HGGT1)	IN, USA	
	qTotalT+T3-19	10	S_14993749	32.5	0.01	25.88	*	IN, USA	
	qTotalT+T3-20	10	S_145163581	83.8	0.01	25.53	*	IN, USA	
**PC-8**	*qPC-8-1*	1	S_11167845	22.2	0.02	23.47	Solanesyl diphosphate synthase	IN, USA	
	qPC-8-2	2	S_219507883	129.3	0.01	15.01	*	IN, USA	
	qPC-8-3	3	S_161788660	73.3	0.02	25.03	*	IN, USA	
	qPC-8-4	5	S_1824267	2.6	0.01	16.61	*	IN, USA	
	qPC-8-5	5	S_140093904	71.2	0.04	42.15	Chorismate mutase, tocopherol cyclase (VTE1)	IN, USA	
	qPC-8-6	6	S_165195245	101.9	0.03	36.09	*	IN, USA	
	qPC-8-7	7	S_14912737	41.4	0.04	42.78	1-deoxy-D-xylulose 5-phosphate synthase 2 (dxs2)	IN, USA	
	qPC-8-8	9	S_20333901	36.1	0.03	30.03	1-deoxy-D-xylulose 5-phosphate synthase 3 (dxs3)	IN, USA	
**QTL Identified in the N6 by NC296 RIL population (*n*=213)**									(Fenton et al., 2018)
**δT/αT**	*qδT/αT*	1	*	18.8–23.9	4.60	2.61	*	IN, USA	
**δT3**	*qδT3*	1	*	28.9–34.2	4.46	1.24	*	IN, USA	
**TT**	*qTT*	1	*	28.8–35	4.42	2.31	*	IN, USA	
**TT3**	*qTT3*	1	*	112.5–121.5	3.98	4.62	*	IN, USA	
**αT/γT**	*qαT/γT*	5	*	42.3–47	7.33	4.57	*	IN, USA	
**δT**	*qδT*	5	*	46.9–50	7.84	20.20	*ZmVTE4*	IN, USA	
**γT**	*qγT*	5	*	47.9–49.6	12.48	24.97	*ZmVTE4*	IN, USA	
**γT/(γT+αT)**	*qγT/(γT+αT)*	5	*	47.9–49.3	30.07	52.85	*ZmVTE4*	IN, USA	
**δT/αT**	*qδT/αT*	5	*	50–53.1	4.87	13.29	*	IN, USA	
**δT/(γT+αT)**	*qδT/(γT+αT)*	5	*	56.1–60.2	5.13	12.98	*	IN, USA	
**αT3/γT3**	*qαT3/γT3*	5	*	56.6–60.9	12.49	19.80	*	IN, USA	
**γT3/(γT3+αT3)**	*qγT3/(γT3+αT3)*	5	*	57.7–60.9	15.75	26.56	*	IN, USA	
**αT3**	*qαT3*	5	*	59.8–63.9	6.43	13.97	*	IN, USA	

**TT+TT3**	*qTT+TT3*	5	*	75.6–78.6	3.98	7.01	*	IN, USA
**δT3/(γT3+αT3)**	*qδT3/(γT3+αT3)*	5	*	83.4–86.4	4.54	8.90	*	IN, USA
**δT3/γT3**	*qδT3/γT3*	5	*	83.4–86.4	4.54	8.90	*	IN, USA
**γT**	*qγT*	5	*	99.4–102.5	11.93	25.65	*	IN, USA
**αT**	*qαT*	5	*	95.9–103.6	4.39	5.53	*	IN, USA
**TT3**	*qTT3*	5	*	98.8–103.5	12.17	23.70	*	IN, USA
**TT/TT3**	*qTT/TT3*	5	*	100.8–107.4	10.53	16.40	*	IN, USA
**δT/γT**	*qδT/γT*	6	*	57.7–59	4.64	6.15	*	IN, USA
**TT+TT3**	*qTT+TT3*	7	*	66.6–72.5	5.07	2.04	*	IN, USA
**γT3**	*qγT3*	7	*	69.6–72.2	6.63	0.78	*	IN, USA
**TT**	*qTT*	7	*	69.4–73.9	5.98	2.46	*	IN, USA
**γT3/(γT3+αT3)**	*qγT3/(γT3+αT3)*	9	*	51.9–55.9	13.54	19.58	*	IN, USA
**αT3/γT3**	*qαT3/γT3*	9	*	56.2–59.3	9.37	14.16	*	IN, USA
**TT3**	*qTT3*	9	*	62.8–65.6	14.83	21.67	*ZmVTE2*	IN, USA
**TT/TT3**	*qTT/TT3*	9	*	61–65.4	8.24	8.01	*ZmVTE2*	IN, USA
**δT3**	*qδT3*	9	*	67–74.8	8.14	17.32	*ZmHGGT1*	IN, USA
**δT3/(γT3+αT3)**	*qδT3/(γT3+αT3)*	9	*	68.4–73.5	11.71	25.05	*ZmHGGT1*	IN, USA
**δT3/γT3**	*qδT3/γT3*	9	*	68.4–73.5	11.71	25.05	*ZmHGGT1*	IN, USA
**QTL Identified in the E2558 by Co125 RIL population (*n*=197)**								
**αT/γT**	*qαT/γT*	1	*	80.7–82.9	2.94	0.10	*	IN, USA
**αT**	*qαT*	1	*	100.2–104.2	4.45	0.39	*	IN, USA
**γT3**	*qγT3*	1	*	100.3–103.9	4.24	0.66	*	IN, USA
**TT+TT3**	*qTT+TT3*	1	*	99.7–105	2.84	0.10	*	IN, USA
**δT3/γT3**	*qδT3/γT3*	1	*	158.7–163.8	2.63	0.38	*	IN, USA
**TT**	*qTT*	2	*	33.3–38.5	3.03	3.74	*	IN, USA
**αT3/γT3**	*qαT3/γT3*	2	*	46.5–49.6	5.57	16.51	*	IN, USA
**αT3/γT3**	*qαT3/γT3*	2	*	55.7–59.2	2.84	2.41	*	IN, USA
**αT3/γT3**	*qαT3/γT3*	3	*	51.6–56.5	3.01	3.03	*	IN, USA
**δT**	*qδT*	3	*	62.5–65.6	6.27	8.26	*	IN, USA
**δT(γT+αT)**	*qδT(γT+αT)*	3	*	62.4–65.7	5.29	7.63	*	IN, USA
**δT/γT**	*qδT/γT*	3	*	63–65.6	5.67	5.71	*	IN, USA
**δT/αT**	*qδT/αT*	4	*	33.8–39	3.03	0.10	*	IN, USA
**TT/TT3**	*qTT/TT3*	5	*	35.7–37.3	3.53	9.94	*	IN, USA
**δT3(γT3+αT3)**	*qδT3(γT3+αT3)*	5	*	44.4–47.9	3.36	2.31	*	IN, USA
**δT/γT**	*qδT/γT*	5	*	47.2–51	9.63	18.53	*ZmVTE4*	IN, USA
**αT/γT**	*qαT/γT*	5	*	49.1–52.2	6.96	15.68	*ZmVTE4*	IN, USA
**αT**	*qαT*	5	*	49.4–58.8	2.92	2.99	*	IN, USA
**δT/γT**	*qδT/γT*	5	*	58.9–62.1	2.92	7.92	*	IN, USA
**αT3/γT3**	*qαT3/γT3*	5	*	69.4–74.3	3.86	6.43	*ZmVTE1*	IN, USA
**δT3**	*qδT3*	5	*	69.2–71.7	2.68	1.41	*ZmVTE1*	IN, USA
**TT+TT3**	*qTT+TT3*	5	*	72–78.4	7.63	9.74	*	IN, USA
**δT**	*qδT*	5	*	74–78.9	4.89	3.25	*ZmHPPD1*	IN, USA
**TT3**	*qTT3*	5	*	73.3–85.3	2.61	6.69	*ZmHPPD1*	IN, USA
**αT3/γT3**	*qαT3/γT3*	5	*	86.2–92.1	5.45	6.92	*	IN, USA
**δT(γT+αT)**	*qδT(γT+αT)*	5	*	105.9–109.6	3.36	4.35	*	IN, USA
**γT**	*qγT*	6	*	12.4–14.4	2.69	4.39	*	IN, USA
**γT/(γT+αT)**	*qγT/(γT+αT)*	6	*	12.4–14.4	2.69	4.39	*	IN, USA
**γT3/(γT3+αT3)**	*qγT3/(γT3+αT3)*	7	*	57.5–64.2	2.75	7.05	*	IN, USA
**γT**	*qγT*	7	*	57.1–64.2	2.58	5.63	*	IN, USA
**TT3**	*qTT3*	8	*	57.9–62.9	2.70	5.28	*	IN, USA
**δT/αT**	*qδT/αT*	8	*	57.8–66	2.52	2.45	*	IN, USA

**αT/γT**	*qαT/γT*	9	*	50.4–56.5	3.99	15.26	*	IN, USA	
**δT3/γT3**	*qδT3/γT3*	9	*	52.2–53.2	21.95	38.95	*	IN, USA	
**TT+TT3**	*qTT+TT3*	9	*	54.9–55.8	18.48	26.87	*	IN, USA	
**δT/αT**	*qδT/αT*	9	*	54.2–55.4	10.98	13.96	*	IN, USA	
**δT3/αT3**	*qδT3/αT3*	9	*	53.5–58.8	7.91	13.52	*	IN, USA	
**αT/γT**	*qαT/γT*	9	*	54–57.6	13.04	22.98	*	IN, USA	
**δT**	*qδT*	9	*	54.1–56.5	7.82	18.41	*	IN, USA	
**γT**	*qγT*	9	*	55.1–55.9	57.19	29.45	*	IN, USA	
**γT/(γT+αT)**	*qγT/(γT+αT)*	9	*	55.1–55.9	57.19	29.45	*	IN, USA	
**γT3/(γT3+αT3)**	*qγT3/(γT3+αT3)*	9	*	55.5–57.8	12.65	39.15	*	IN, USA	
**δT3(γT3+αT3)**	*qδT3(γT3+αT3)*	9	*	55.5–57.8	23.53	63.74	*	IN, USA	
**δT3**	*qδT3*	9	*	56.5–60	42.26	74.09	*ZmHGGT1*	IN, USA	
**γT3**	*qγT3*	9	*	56.5–60	36.46	65.11	*ZmHGGT1*	IN, USA	
**TT**	*qTT*	9	*	56.5–58.8	13.02	21.44	*	IN, USA	
**αT3**	*qαT3*	9	*	56.5–61	2.81	6.32	*ZmHGGT1*	IN, USA	
**TT3**	*qTT3*	9	*	56.7–58.8	10.34	24.43	*	IN, USA	
**TT+TT3**	*qTT+TT3*	9	*	56.5–61	3.18	0.10	*ZmHGGT1*	IN, USA	
**αT3/γT3**	*qαT3/γT3*	9	*	59–60.3	62.60	29.63	*ZmHGGT1*	IN, USA	
**γT/(γT+αT)**	*qγT/(γT+αT)*	9	*	59–60.3	62.60	29.63	*ZmHGGT1*	IN, USA	
**γT3/(γT3+αT3)**	*qγT3/(γT3+αT3)*	9	*	59–63	13.42	4.08	*ZmHGGT1*	IN, USA	
**δT3/γT3**	*qδT3/γT3*	9	*	67.9–72	2.84	0.45	*	IN, USA	
**δT3**	*qδT3*	9	*	71.5–73.2	6.80	5.43	*	IN, USA	
**γT/(γT+αT)**	*qγT/(γT+αT)*	9	*	99–104.4	2.98	1.33	*	IN, USA	
**δT3/γT3**	*qδT3/γT3*	10	*	44.7–48.5	3.63	0.10	*	IN, USA	
**γT3/(γT3+αT3)**	*qγT3/(γT3+αT3)*	10	*	52–55	3.42	2.85	*	IN, USA	
**αT3**	*qαT3*	10	*	51.6–56.1	2.99	3.01	*	IN, USA	
**QTL Identified in one or more of the 6 RIL populations**									(Wang et al., 2018)
**AT**	*qAT*	1	*	2.93-3.41	3.91	9.06	*	China	
**GT**	*qGT*	1	*	6.95-10.06	3.85	7.48	*GRMZM2G112728*	China	
**TT**	*qTT*	1	*	7.43-9.81	4.81	9.3	*	China	
**DT**	*qDT*	1	*	6.25-11.39	3.58	6.23	*	China	
**GT**	*qGT*	1	*	12.13-21.50	3.17	2.86	*	China	
**TT**	*qTT*	1	*	22.38-46.68	3.49	3.6	*GRMZM2G164562*	China	
**GT**	*qGT*	1	*	27.80-56.89	3.58	4.89	*GRMZM2G141273*	China	
**TT**	*qTT*	1	*	30.20-58.46	4.85	6.92	*	China	
**AT**	*qAT*	1	*	51.01-76.87	6.98	11.58	*GRMZM2G396212*	China	
**DT**	*qDT*	1	*	84.63-142.37	3.75	6.53	*GRMZM2G359822*	China	
**DT**	*qDT*	1	*	98.16-174.37	7.17	13.12	*GRMZM2G082998*	China	
**TT**	*qTT*	1	*	213.57-234.79	3.52	6.6	*	China	
**DT**	*qDT*	1	*	223.76-235.46	4.42	7.65	*	China	
**AT**	*qAT*	1	*	224.38-230.83	7.28	8.91	*	China	
**GT**	*qGT*	1	*	225.04-228.30	26.19	34.29	*	China	
**TT**	*qTT*	1	*	226.14-228.72	24.98	36.34	*	China	
**RT**	*qRT*	1	*	251.16-262.40	3.59	6.61	*GRMZM2G448446*	China	
**AT**	*qAT*	1	*	253.09-273.45	4.48	8.27	*GRMZM2G027059*	China	
**RT**	*qRT*	1	*	273.4-285.13	3.97	7.09	*	China	
**AT**	*qAT*	1	*	286.51-292.13	3.65	7.63	*GRMZM2G156516*	China	
**AT**	*qAT*	1	*	287.71-298.05	3.15	5.01	*	China	
**GT**	*qGT*	2	*	30.19-69.18	3.26	4.16	*GRMZM2G437912*	China	
**GT**	*qGT*	2	*	34.67-114.20	3.05	4	*	China	
**DT**	*qDT*	2	*	77.70-181.42	3.22	6.7	*GRMZM2G466543*	China	

**AT**	*qAT*	2	*	208.21-222.47	3.13	3.98	*GRMZM2G113476*	China
**DT**	*qDT*	2	*	203.15-222.30	4.81	8.59	*AC194970.5 FG001*	China
**RT**	*qRT*	2	*	212.54-218.46	8.29	15.8	*	China
**GT**	*qGT*	3	*	9.04-22.39	3.74	7.08	*GRMZM2G056975*	China
**GT**	*qGT*	3	*	22.00-48.81	4.25	6	*GRMZM2G419111*	China
**AT**	*qAT*	3	*	162.15-210.52	4.49	8.91	*GRMZM5G856881*	China
**RT**	*qRT*	3	*	220.40-227.70	3.03	4.48	*	China
**RT**	*qRT*	3	*	223.91-227.41	4.44	8.37	*	China
**GT**	*qGT*	4	*	2.86-5.83	5.62	7	*GRMZM2G006480*	China
**AT**	*qAT*	4	*	175.27-186.06	4.53	7.4	*GRMZM2G161566*	China
**DT**	*qDT*	4	*	181.23-189.61	4.18	8.9	*	China
**GT**	*qGT*	5	*	2.28-5.90	3.21	6.33	*	China
**DT**	*qDT*	5	*	15.14-25.39	4.42	9.4	*GRMZM2G454719*	China
**GT**	*qGT*	5	*	19.34-25.05	26.79	46.56	*GRMZM2G084942*	China
**RT**	*qRT*	5	*	19.67-25.39	7.41	12.05	*GRMZM2G085117*	China
**TT**	*qTT*	5	*	22.10-24.71	29.47	48.69	*	China
**AT**	*qAT*	5	*	22.10-24.71	13.56	19.99	*	China
**RT**	*qRT*	5	*	19.68-26.56	6.55	9.32	*	China
**TT**	*qTT*	5	*	18.67-43.42	7.83	11.44	*	China
**GT**	*qGT*	5	*	14.84-49.80	6.3	8.61	*	China
**GT**	*qGT*	5	*	20.49-29.99	11.92	19.11	*	China
**TT**	*qTT*	5	*	21.42-29.92	12.89	21.78	*	China
**DT**	*qDT*	5	*	18.54-51.43	3.1	6.47	*	China
**DT**	*qDT*	5	*	14.02-69.30	3.24	5.26	*	China
**DT**	*qDT*	5	*	63.32-81.27	5.77	10.87	*GRMZM2G477236*	China
**GT**	*qGT*	5	*	191.37-200.60	11.87	13.08	*AC209374.4_FG002*	China
**RT**	*qRT*	5	*	191.09-202.81	3.73	7.95	*GRMZM2G035213*	China
**RT**	*qRT*	5	*	197.70-205.02	3.91	7.26	*GRMZM2G105644*	China
**RT**	*qRT*	5	*	198.50-202.05	14.74	26.34	*GRMZM2G070218*	China
**AT**	*qAT*	5	*	196.68-208.76	3.46	6.92	*	China
**RT**	*qRT*	5	*	197.11-200.60	25.9	40.59	*	China
**AT**	*qAT*	5	*	196.18-202.16	9.55	13.16	*	China
**RT**	*qRT*	5	*	197.84-201.61	15.48	28.3	*	China
**AT**	*qAT*	5	*	197.11-200.60	22.44	35.07	*	China
**GT**	*qGT*	5	*	196.68-203.80	5.77	7.75	*	China
**RT**	*qRT*	5	*	211.94-216.50	3.04	4.63	*	China
**RT**	*qRT*	5	*	213.88-215.59	3.47	4.44	*	China
**DT**	*qDT*	6	*	16.17-89.91	3.6	6.32	*GRMZM2G365961*	China
**DT**	*qDT*	6	*	97.28-108.26	4.91	8.72	*	China
**AT**	*qAT*	6	*	133.05-150.40	3.85	8.69	*GRMZM5G878070*	China
**TT**	*qTT*	6	*	137.69-150.48	3.62	7.18	*GRMZM2G137151*	China
**RT**	*qRT*	7	*	5.56-9.70	3.66	6.94	*	China
**DT**	*qDT*	7	*	21.27-73.50	4.96	8.97	*GRMZM2G374213*	China
**DT**	*qDT*	7	*	86.32-92.94	4.44	8.16	*	China
**TT**	*qTT*	7	*	27.98-128.71	3.59	4.11	*	China
**AT**	*qAT*	7	*	123.67-136.86	3.84	7.82	*GRMZM2G121546*	China
**RT**	*qRT*	7	*	125.14-143.29	3.85	4.32	*	China
**AT**	*qAT*	7	*	130.90-155.37	3.25	3.62	*	China
**DT**	*qDT*	8	*	7.39-11.36	3.11	5.54	*GRMZM2G058404*	China
**GT**	*qGT*	8	*	6.14-10.09	7	9.65	*GRMZM2G036290*	China
**TT**	*qTT*	8	*	7.87-8.73	11.05	17.1	*	China
**GT**	*qGT*	8	*	10.00-13.69	3.08	4.26	*	China

**AT**	*qAT*	8	*	21.30-123.80	3.47	7.93	*GRMZM2G315125*	China
**RT**	*qRT*	8	*	118.86-150.15	3.7	4.11	*	China
**DT**	*qDT*	8	*	157.40-162.50	3.23	6.86	*	China
**GT**	*qGT*	8	*	162.50-168.93	4.66	8.94	*GRMZM2G172032*	China
**TT**	*qTT*	8	*	162.50-168.93	4.14	8.14	*	China
**DT**	*qDT*	8	*	165.35-168.24	6.92	12.68	*	China
**AT**	*qAT*	9	*	17.12-23.46	4.77	7.65	*GRMZM2G173641*	China
**GT**	*qGT*	9	*	129.19-141.78	4.67	6.65	*GRMZM2G145451*	China
**TT**	*qTT*	9	*	129.19-143.03	3.16	4.59	*GRMZM2G036861*	China
**RT**	*qRT*	10	*	10.19-80.40	3.76	6.75	*GRMZM2G014376*	China
**AT**	*qAT*	10	*	117.27-132.54	6.86	12.53	*	China
**GT**	*qGT*	10	*	127.35-136.01	6.79	10.42	*	China
**TT**	*qTT*	10	*	125.13-132.12	5.67	9.06	*	China
**SNPs Identified by GWAS in the Association panel population**								
**AT**	*qAT-1*	1	chr1.S_3038971	3,038,971	4.87E-07	*	*GRMZM2G436226*	China
	qAT-2	1	chr1.S_4040365	4,040,365	1.47E-06	*	*GRMZM2G013634*	China
	qAT-1	2	chr2.S_136272058	136,272,058	5.03E-07	*	*GRMZM2G043226*	China
	qAT-2	2	chr2.S_136272060	136,272,060	5.03E-07	*	*GRMZM2G043226*	China
	qAT-3	2	chr2.S_136272061	136,272,061	5.03E-07	*	*GRMZM2G043226*	China
	qAT-4	2	chr2.S_136272084	136,272,084	5.03E-07	*	*GRMZM2G043226*	China
	qAT-5	2	chr2.S_136272140	136,272,140	5.03E-07	*	*GRMZM2G043226*	China
	qAT-6	2	chr2.S_136272141	136,272,141	5.03E-07	*	*GRMZM2G043226*	China
	qAT-7	2	chr2.S_136272169	136,272,169	5.03E-07	*	*GRMZM2G043226*	China
	qAT-8	2	chr2.S_136272394	136,272,394	5.23E-07	*	*GRMZM2G043226*	China
	qAT-9	2	chr2.S_136272396	136,272,396	5.23E-07	*	*GRMZM2G043226*	China
	qAT-10	2	chr2.S_136272403	136,272,403	5.14E-07	*	*GRMZM2G043226*	China
	qAT-11	3	chr3.S_2139263	2,139,263	5.23E-07	*	*GRMZM2G170013*	China
	qAT-12	3	chr3.S_232043044	232,043,044	1.47E-06	*	*GRMZM5G875655*	China
	qAT-13	3	chr3.S_232043153	232,043,153	1.47E-06	*	*GRMZM5G875655*	China
	qAT-14	3	chr3.S_232043402	232,043,402	1.47E-06	*	*GRMZM5G875655*	China
	qAT-15	3	chr3.S_232044135	232,044,135	1.63E-06	*	*GRMZM5G875655*	China
	qAT-16	4	chr4.S_201887561	201,887,561	6.58E-07	*	*GRMZM2G000608*	China
	qAT-17	4	chr4.S_239794450	239,794,450	6.95E-07	*	*GRMZM2G150251*	China
	qAT-18	5	chr5.S_90530346	90,530,346	1.28E-06	*	*GRMZM2G046155*	China
	qAT-19	5	chr5.S_196508571	196,508,571	2.40E-07	*	*GRMZM2G022645*	China
	qAT-20	5	chr5.S_196510693	196,510,693	4.99E-07	*	*GRMZM2G022645*	China
	qAT-21	5	chr5.S_196511036	196,511,036	1.35E-08	*	*GRMZM2G022645*	China
	qAT-22	5	chr5.S_198879413	198,879,413	4.30E-07	*	*GRMZM2G413829*	China
	qAT-23	5	chr5.S_198879415	198,879,415	4.30E-07	*	*GRMZM2G413829*	China
	qAT-24	5	chr5.S_199371029	199,371,029	1.09E-08	*	*GRMZM2G376067*	China
	qAT-25	5	chr5.S_199373005	199,373,005	1.09E-08	*	*GRMZM2G376067*	China
	qAT-26	5	chr5.S_199373231	199,373,231	1.09E-08	*	*GRMZM2G376067*	China
	qAT-27	5	chr5.S_199373289	199,373,289	1.09E-08	*	*GRMZM2G376067*	China
	qAT-28	5	chr5.S_199373414	199,373,414	4.41E-09	*	*GRMZM2G376067*	China
	qAT-29	5	chr5.S_199377626	199,377,626	1.09E-08	*	*GRMZM2G027741*	China
	qAT-30	5	chr5.S_199378010	199,378,010	1.09E-08	*	*GRMZM2G027741*	China
	qAT-31	5	chr5.S_199379904	199,379,904	1.09E-08	*	*GRMZM2G027741*	China
	qAT-32	5	chr5.S_199380227	199,380,227	1.09E-08	*	*GRMZM2G027741*	China
	qAT-33	5	chr5.S_199380407	199,380,407	2.15E-09	*	*GRMZM2G027741*	China
	qAT-34	5	PZE-105146513	199,647,442	3.82E-11	*	*GRMZM5G823157*	China
	qAT-35	5	chr5.S_200114897	200,114,897	6.01E-09	*	*GRMZM2G108996*	China

qAT-36	5	chr5.S_200114905	200,114,905	1.84E-06	*	*GRMZM2G108996*	China
qAT-37	5	chr5.S_200114909	200,114,909	4.19E-07	*	*GRMZM2G108996*	China
qAT-38	5	chr5.S_200114947	200,114,947	2.25E-09	*	*GRMZM2G108996*	China
qAT-39	5	chr5.S_200114950	200,114,950	2.83E-10	*	*GRMZM2G108996*	China
qAT-40	5	chr5.S_200114951	200,114,951	2.83E-10	*	*GRMZM2G108996*	China
qAT-41	5	chr5.S_200114953	200,114,953	2.83E-10	*	*GRMZM2G108996*	China
qAT-42	5	chr5.S_200114954	200,114,954	2.83E-10	*	*GRMZM2G108996*	China
qAT-43	5	chr5.S_200294718	200,294,718	3.10E-10	*	*GRMZM2G034876*	China
qAT-44	5	chr5.S_200294736	200,294,736	3.89E-09	*	*GRMZM2G034876*	China
qAT-45	5	chr5.S_200294763	200,294,763	3.31E-07	*	*GRMZM2G034876*	China
qAT-46	5	PZE-105147666	200,300,836	6.81E-11	*	*GRMZM2G034876*	China
qAT-47	5	chr5.S_200367420	200,367,420	4.41E-21	*	*GRMZM2G035213*	China
qAT-48	5	PZB02283.1	200,367,532	2.55E-18	*	*GRMZM2G035213*	China
qAT-49	5	chr5.S_200369079	200,369,079	8.93E-17	*	*GRMZM2G035213*	China
qAT-50	5	PZE-105147738	200,369,124	1.86E-16	*	*GRMZM2G035213*	China
qAT-51	5	chr5.S_200369163	200,369,163	4.08E-22	*	*GRMZM2G035213*	China
qAT-52	5	chr5.S_200370009	200,370,009	5.03E-15	*	*GRMZM2G035213*	China
qAT-53	5	chr5.S_200370013	200,370,013	2.00E-13	*	*GRMZM2G035213*	China
qAT-54	5	chr5.S_200370675	200,370,675	6.78E-08	*	*GRMZM2G035213*	China
qAT-55	5	chr5.S_200370683	200,370,683	6.75E-13	*	*GRMZM2G035213*	China
qAT-56	5	chr5.S_200370743	200,370,743	5.92E-07	*	*GRMZM2G035213*	China
qAT-57	5	chr5.S_200370781	200,370,781	3.76E-15	*	*GRMZM2G035213*	China
qAT-58	5	chr5.S_200370782	200,370,782	8.65E-16	*	*GRMZM2G035213*	China
qAT-59	5	chr5.S_200380468	200,380,468	4.22E-07	*	*GRMZM2G035213*	China
qAT-60	5	chr5.S_200380534	200,380,534	8.09E-07	*	*GRMZM2G035213*	China
qAT-61	5	chr5.S_200380572	200,380,572	1.02E-15	*	*GRMZM2G167431*	China
qAT-62	5	chr5.S_200380573	200,380,573	2.94E-08	*	*GRMZM2G167431*	China
qAT-63	5	chr5.S_200380575	200,380,575	1.02E-15	*	*GRMZM2G167431*	China
qAT-64	5	chr5.S_200380576	200,380,576	2.96E-19	*	*GRMZM2G167431*	China
qAT-65	5	chr5.S_200380614	200,380,614	2.28E-09	*	*GRMZM2G167431*	China
qAT-66	5	chr5.S_200380699	200,380,699	1.44E-15	*	*GRMZM2G167431*	China
qAT-67	5	chr5.S_200380732	200,380,732	1.44E-15	*	*GRMZM2G167431*	China
qAT-68	5	chr5.S_200380825	200,380,825	1.21E-12	*	*GRMZM2G167431*	China
qAT-69	5	chr5.S_200381453	200,381,453	1.21E-12	*	*GRMZM2G167431*	China
qAT-70	5	chr5.S_200381475	200,381,475	1.21E-12	*	*GRMZM2G167431*	China
qAT-71	5	chr5.S_200382168	200,382,168	5.25E-13	*	*GRMZM2G167431*	China
qAT-72	5	chr5.S_200382301	200,382,301	2.55E-13	*	*GRMZM2G167431*	China
qAT-73	5	chr5.S_200382311	200,382,311	1.71E-06	*	*GRMZM2G167431*	China
qAT-74	5	chr5.S_200435108	200,435,108	1.39E-17	*	*GRMZM2G325019*	China
qAT-75	5	chr5.S_200435117	200,435,117	1.98E-08	*	*GRMZM2G325019*	China
qAT-76	5	PZE-105147471	200,435,300	2.11E-14	*	*GRMZM2G325019*	China
qAT-77	5	chr5.S_200436292	200,436,292	5.10E-21	*	*GRMZM2G325019*	China
qAT-78	5	chr5.S_200437084	200,437,084	9.09E-13	*	*GRMZM2G325019*	China
qAT-79	5	chr5.S_200437319	200,437,319	2.17E-09	*	*GRMZM2G325019*	China
qAT-80	5	chr5.S_200437366	200,437,366	9.99E-09	*	*GRMZM2G325019*	China
qAT-81	5	chr5.S_200437468	200,437,468	1.53E-10	*	*GRMZM2G325019*	China
qAT-82	5	chr5.S_200437599	200,437,599	4.64E-10	*	*GRMZM2G325019*	China
qAT-83	5	chr5.S_200437607	200,437,607	1.26E-09	*	*GRMZM2G325019*	China
qAT-84	5	chr5.S_200437642	200,437,642	5.67E-13	*	*GRMZM2G325019*	China
qAT-85	5	chr5.S_200437660	200,437,660	7.81E-08	*	*GRMZM2G325019*	China
qAT-86	5	chr5.S_200437708	200,437,708	1.46E-10	*	*GRMZM2G325019*	China
qAT-87	5	chr5.S_200437813	200,437,813	6.49E-07	*	*GRMZM2G325019*	China

qAT-88	5	chr5.S_200437879	200,437,879	5.60E-17	*	*GRMZM2G325019*	China
qAT-89	5	chr5.S_200437882	200,437,882	6.44E-09	*	*GRMZM2G325019*	China
qAT-90	5	chr5.S_200437909	200,437,909	6.44E-09	*	*GRMZM2G325019*	China
qAT-91	5	chr5.S_200438027	200,438,027	2.51E-15	*	*GRMZM2G325019*	China
qAT-92	5	chr5.S_200438197	200,438,197	3.35E-13	*	*GRMZM2G325019*	China
qAT-93	5	chr5.S_200438220	200,438,220	1.51E-09	*	*GRMZM2G325019*	China
qAT-94	5	chr5.S_200438234	200,438,234	5.21E-09	*	*GRMZM2G325019*	China
qAT-95	5	chr5.S_200438247	200,438,247	4.18E-07	*	*GRMZM2G325019*	China
qAT-96	5	chr5.S_200438314	200,438,314	3.50E-12	*	*GRMZM2G325019*	China
qAT-97	5	chr5.S_200438352	200,438,352	8.14E-12	*	*GRMZM2G325038*	China
qAT-98	5	chr5.S_200438462	200,438,462	5.80E-12	*	*GRMZM2G325038*	China
qAT-99	5	chr5.S_200438465	200,438,465	3.65E-12	*	*GRMZM2G325038*	China
qAT-100	5	chr5.S_200438466	200,438,466	3.65E-12	*	*GRMZM2G325038*	China
qAT-101	5	chr5.S_200438485	200,438,485	3.19E-09	*	*GRMZM2G325038*	China
qAT-102	5	chr5.S_200438560	200,438,560	1.80E-10	*	*GRMZM2G325038*	China
qAT-103	5	chr5.S_200438590	200,438,590	2.27E-11	*	*GRMZM2G325038*	China
qAT-104	5	chr5.S_200438694	200,438,694	1.24E-13	*	*GRMZM2G325038*	China
qAT-105	5	chr5.S_200438801	200,438,801	1.91E-07	*	*GRMZM2G325038*	China
qAT-106	5	chr5.S_200438905	200,438,905	6.38E-07	*	*GRMZM2G325038*	China
qAT-107	5	chr5.S_200528650	200,528,650	2.02E-07	*	*GRMZM2G108087*	China
qAT-108	5	chr5.S_200528660	200,528,660	2.02E-07	*	*GRMZM2G108087*	China
qAT-109	5	chr5.S_200528663	200,528,663	6.56E-07	*	*GRMZM2G108087*	China
qAT-110	5	chr5.S_200552005	200,552,005	7.18E-11	*	*GRMZM2G092749*	China
qAT-111	5	chr5.S_200552079	200,552,079	9.38E-09	*	*GRMZM2G092749*	China
qAT-112	5	chr5.S_200684527	200,684,527	4.82E-10	*	*GRMZM2G150374*	China
qAT-113	5	chr5.S_200684528	200,684,528	4.82E-10	*	*GRMZM2G150374*	China
qAT-114	5	chr5.S_200684529	200,684,529	4.82E-10	*	*GRMZM2G150374*	China
qAT-115	5	chr5.S_200684530	200,684,530	4.82E-10	*	*GRMZM2G150374*	China
qAT-116	5	chr5.S_200686561	200,686,561	4.40E-10	*	*GRMZM2G150374*	China
qAT-117	5	chr5.S_200822060	200,822,060	2.44E-08	*	*GRMZM2G046748*	China
qAT-118	5	chr5.S_200824912	200,824,912	8.22E-09	*	*GRMZM2G046748*	China
qAT-119	5	PUT-163a-148963345-597	200,824,929	1.18E-06	*	*GRMZM2G046748*	China
qAT-120	5	SYN5678	200,871,732	1.17E-08	*	*GRMZM2G347767*	China
qAT-121	5	PZE-105148878	200,877,505	1.59E-07	*	*GRMZM2G046244*	China
qAT-122	5	chr5.S_200878876	200,878,876	3.30E-10	*	*GRMZM2G046244*	China
qAT-123	5	chr5.S_200878917	200,878,917	4.57E-10	*	*GRMZM2G046244*	China
qAT-124	5	chr5.S_200878929	200,878,929	1.05E-09	*	*GRMZM2G046244*	China
qAT-125	5	chr5.S_201072279	201,072,279	1.00E-06	*	*GRMZM2G050734*	China
qAT-126	5	chr5.S_201073030	201,073,030	1.58E-06	*	*GRMZM2G050734*	China
qAT-127	5	chr5.S_201226344	201,226,344	1.95E-07	*	*GRMZM2G417229*	China
qAT-128	5	chr5.S_201267187	201,267,187	6.67E-09	*	*GRMZM2G320307*	China
qAT-129	5	chr5.S_201403633	201,403,633	6.96E-07	*	*GRMZM2G140739*	China
qAT-130	5	chr5.S_201471104	201,471,104	1.04E-06	*	*GRMZM2G147377*	China
qAT-131	5	chr5.S_201938646	201,938,646	9.39E-10	*	*GRMZM2G009506*	China
qAT-132	5	chr5.S_202054574	202,054,574	5.94E-08	*	*GRMZM2G020040*	China
qAT-133	5	chr5.S_202058856	202,058,856	4.37E-08	*	*GRMZM2G020040*	China
qAT-134	5	chr5.S_202059377	202,059,377	5.94E-08	*	*GRMZM2G020040*	China
qAT-135	5	SYN35167	202,061,590	7.65E-08	*	*GRMZM2G315127*	China
qAT-136	5	chr5.S_202063233	202,063,233	5.79E-08	*	*GRMZM2G315127*	China
qAT-137	5	SYN35165	202,065,247	4.30E-07	*	*GRMZM2G315127*	China
qAT-138	5	chr5.S_202084979	202,084,979	3.49E-08	*	*GRMZM2G180983*	China
qAT-139	5	chr5.S_202085266	202,085,266	5.78E-08	*	*GRMZM2G180983*	China

	qAT-140	5	SYN35180	202,085,442	1.27E-07	*	*GRMZM2G180983*	China
	qAT-141	5	SYN35179	202,085,459	8.76E-08	*	*GRMZM2G180983*	China
	qAT-142	5	chr5.S_202095425	202,095,425	6.34E-08	*	*GRMZM2G481605*	China
	qAT-143	5	chr5.S_202221152	202,221,152	3.91E-07	*	*GRMZM2G475349*	China
	qAT-144	5	chr5.S_202286259	202,286,259	1.94E-07	*	*GRMZM2G164912*	China
	qAT-145	5	chr5.S_202286260	202,286,260	1.94E-07	*	*GRMZM2G164912*	China
	qAT-146	5	chr5.S_202286261	202,286,261	7.21E-07	*	*GRMZM2G164912*	China
	qAT-147	5	chr5.S_202287726	202,287,726	2.18E-07	*	*GRMZM2G164912*	China
	qAT-148	5	chr5.S_213281088	213,281,088	1.17E-06	*	*GRMZM2G018484*	China
	qAT-149	5	chr5.S_213281249	213,281,249	2.22E-07	*	*GRMZM2G018484*	China
	qAT-150	5	chr5.S_213282225	213,282,225	4.01E-08	*	*GRMZM2G018103*	China
	qAT-151	5	chr5.S_213282760	213,282,760	4.27E-08	*	*GRMZM2G018103*	China
**GT**	*qGT-1*	1	chr1.S_228564342	228,564,342	1.04E-07	*	*GRMZM5G888696*	China
	qGT-2	1	chr1.S_228564433	228,564,433	1.04E-07	*	*GRMZM5G888696*	China
	qGT-3	1	chr1.S_228564475	228,564,475	1.04E-07	*	*GRMZM5G888696*	China
	qGT-4	1	chr1.S_228564518	228,564,518	1.04E-07	*	*GRMZM5G888696*	China
	qGT-5	1	chr1.S_228564574	228,564,574	7.31E-08	*	*GRMZM5G888696*	China
	qGT-6	1	PZE-101183878	228,564,617	2.20E-07	*	*GRMZM5G888696*	China
	qGT-7	5	chr5.S_175728543	175,728,543	3.33E-07	*	*GRMZM2G024739*	China
	qGT-8	6	chr6.S_92311076	92,311,076	1.19E-06	*	*GRMZM2G146190*	China
	qGT-9	6	chr6.S_92311381	92,311,381	1.19E-06	*	*GRMZM2G146190*	China
	qGT-10	6	chr6.S_92311403	92,311,403	1.19E-06	*	*GRMZM2G146190*	China
	qGT-11	6	chr6.S_92311428	92,311,428	1.19E-06	*	*GRMZM2G146190*	China
	qGT-12	6	chr6.S_92311455	92,311,455	1.19E-06	*	*GRMZM2G146190*	China
	qGT-13	6	chr6.S_92311522	92,311,522	1.19E-06	*	*GRMZM2G146190*	China
	qGT-14	6	chr6.S_92311686	92,311,686	1.19E-06	*	*GRMZM2G146190*	China
	qGT-15	6	chr6.S_104858461	104,858,461	6.75E-08	*	*GRMZM2G169089*	China
	qGT-16	6	chr6.S_104858493	104,858,493	2.21E-08	*	*GRMZM2G169089*	China
	qGT-17	8	chr8.S_38490100	38,490,100	3.66E-07	*	*GRMZM2G097103*	China
**TT**	*qTT-1*	1	PZE-101132612	170,961,674	1.16E-06	*	*GRMZM2G115615*	China
	qTT-2	2	chr2.S_149186669	149,186,669	6.06E-08	*	*GRMZM2G175008*	China
	qTT-3	2	chr2.S_149187319	149,187,319	3.29E-07	*	*GRMZM2G175008*	China
	qTT-4	2	chr2.S_149204826	149,204,826	5.88E-07	*	*GRMZM5G816552*	China
	qTT-5	2	chr2.S_149204828	149,204,828	5.88E-07	*	*GRMZM5G816552*	China
	qTT-6	2	chr2.S_149514761	149,514,761	1.32E-07	*	*GRMZM2G079127*	China
	qTT-7	2	chr2.S_149515487	149,515,487	3.42E-08	*	*GRMZM2G079127*	China
	qTT-8	2	chr2.S_149520851	149,520,851	3.09E-09	*	*GRMZM2G079236*	China
	qTT-9	2	chr2.S_149522783	149,522,783	3.09E-09	*	*GRMZM2G079236*	China
	qTT-10	2	chr2.S_149522937	149,522,937	3.09E-09	*	*GRMZM2G079236*	China
	qTT-11	2	chr2.S_149522941	149,522,941	3.09E-09	*	*GRMZM2G079236*	China
	qTT-12	2	chr2.S_149522977	149,522,977	3.09E-09	*	*GRMZM2G079236*	China
	qTT-13	2	chr2.S_149785952	149,785,952	3.65E-09	*	*GRMZM2G377797*	China
	qTT-14	2	chr2.S_150700155	150,700,155	1.11E-07	*	*GRMZM2G007283*	China
	qTT-15	2	chr2.S_150700305	150,700,305	4.77E-09	*	*GRMZM2G007283*	China
	qTT-16	2	chr2.S_150702210	150,702,210	7.20E-09	*	*GRMZM2G007283*	China
	qTT-17	2	chr2.S_150703980	150,703,980	7.38E-09	*	*GRMZM2G007283*	China
	qTT-18	2	chr2.S_150704064	150,704,064	7.38E-09	*	*GRMZM2G007283*	China
	qTT-19	2	chr2.S_150704088	150,704,088	7.38E-09	*	*GRMZM2G007283*	China
	qTT-20	2	chr2.S_207378625	207,378,625	1.90E-09	*	*GRMZM2G022213*	China
	qTT-21	2	chr2.S_207378663	207,378,663	1.90E-09	*	*GRMZM2G022213*	China
	qTT-22	2	chr2.S_207380989	207,380,989	7.97E-08	*	*GRMZM2G022213*	China
	qTT-23	3	chr3.S_2218034	2,218,034	1.87E-07	*	*GRMZM2G159307*	China

	qTT-24	3	chr3.S_2218607	2,218,607	6.77E-07	*	*GRMZM2G159307*	China
	qTT-25	3	chr3.S_2220756	2,220,756	1.87E-07	*	*GRMZM2G159388*	China
	qTT-26	4	chr4.S_18686833	18,686,833	1.65E-07	*	*GRMZM5G892742*	China
	qTT-27	4	chr4.S_18686834	18,686,834	1.65E-07	*	*GRMZM5G892742*	China
	qTT-28	4	PZE-104028481	34,338,513	8.34E-07	*	*GRMZM2G000481*	China
	qTT-29	4	chr4.S_34338552	34,338,552	1.60E-06	*	*GRMZM2G000481*	China
	qTT-30	4	chr4.S_34467924	34,467,924	1.60E-06	*	*GRMZM2G157306*	China
	qTT-31	4	chr4.S_34473783	34,473,783	1.60E-06	*	*GRMZM2G157306*	China
	qTT-32	4	chr4.S_151748204	151,748,204	8.20E-07	*	*GRMZM2G150714*	China
	qTT-33	5	chr5.S_6803634	6,803,634	1.09E-06	*	*GRMZM2G086287*	China
	qTT-34	5	chr5.S_6803876	6,803,876	1.06E-06	*	*GRMZM2G086287*	China
	qTT-35	5	chr5.S_6803903	6,803,903	1.06E-06	*	*GRMZM2G086287*	China
	qTT-36	5	chr5.S_6803934	6,803,934	1.09E-06	*	*GRMZM2G086287*	China
	qTT-37	5	chr5.S_6804176	6,804,176	1.09E-06	*	*GRMZM2G086287*	China
	qTT-38	5	chr5.S_6804312	6,804,312	1.09E-06	*	*GRMZM2G086287*	China
	qTT-39	5	chr5.S_6804406	6,804,406	1.09E-06	*	*GRMZM2G086287*	China
	qTT-40	5	chr5.S_6805126	6,805,126	1.09E-06	*	*GRMZM2G086287*	China
	qTT-41	5	chr5.S_6805234	6,805,234	1.09E-06	*	*GRMZM2G086287*	China
	qTT-42	5	chr5.S_6805341	6,805,341	1.09E-06	*	*GRMZM2G086287*	China
	qTT-43	5	chr5.S_6805362	6,805,362	1.09E-06	*	*GRMZM2G086287*	China
	qTT-44	5	chr5.S_6805384	6,805,384	1.09E-06	*	*GRMZM2G086287*	China
	qTT-45	5	chr5.S_6805416	6,805,416	1.09E-06	*	*GRMZM2G086287*	China
	qTT-46	5	chr5.S_6805480	6,805,480	1.09E-06	*	*GRMZM2G086287*	China
	qTT-47	5	chr5.S_6805760	6,805,760	9.49E-07	*	*GRMZM2G086287*	China
	qTT-48	5	chr5.S_6806066	6,806,066	9.49E-07	*	*GRMZM2G086287*	China
	qTT-49	5	chr5.S_6806090	6,806,090	9.49E-07	*	*GRMZM2G086287*	China
	qTT-50	5	chr5.S_6806177	6,806,177	1.23E-06	*	*GRMZM2G086287*	China
	qTT-51	5	chr5.S_6806219	6,806,219	9.49E-07	*	*GRMZM2G086287*	China
	qTT-52	5	chr5.S_20773412	20,773,412	7.12E-07	*	*GRMZM2G002874*	China
	qTT-53	5	chr5.S_26095529	26,095,529	2.40E-07	*	*GRMZM2G022175*	China
	qTT-54	5	chr5.S_26095624	26,095,624	2.40E-07	*	*GRMZM2G022175*	China
	qTT-55	5	chr5.S_188606921	188,606,921	1.28E-06	*	*GRMZM2G139858*	China
	qTT-56	6	chr6.S_104858404	104,858,404	4.02E-08	*	*GRMZM2G169089*	China
	qTT-57	8	chr8.S_8399315	8,399,315	1.46E-08	*	*GRMZM2G066057*	China
	qTT-58	8	chr8.S_23889464	23,889,464	1.08E-06	*	*GRMZM2G099483*	China
	qTT-59	8	chr8.S_38520871	38,520,871	1.36E-08	*	*GRMZM2G003022*	China
	qTT-60	10	PZE-110061746	116,894,888	5.61E-07	*	*GRMZM2G054632*	China
	qTT-61	10	PZE-110062783	117,774,209	1.05E-06	*	*GRMZM2G009888*	China
**RT**	*qRT-1*	2	chr2.S_5424718	5,424,718	5.95E-07	*	*GRMZM2G055752*	China
	qRT-2	4	chr4.S_54553527	54,553,527	1.71E-06	*	*GRMZM2G121776*	China
	qRT-3	4	chr4.S_136059154	136,059,154	1.42E-06	*	*GRMZM2G067417*	China
	qRT-4	5	chr5.S_1936	1,936	4.87E-08	*	*GRMZM2G356204*	China
	qRT-5	5	chr5.S_108368	108,368	2.97E-08	*	*GRMZM2G089498*	China
	qRT-6	5	chr5.S_108369	108,369	2.97E-08	*	*GRMZM2G089498*	China
	qRT-7	5	chr5.S_109095	109,095	7.78E-08	*	*GRMZM2G089498*	China
	qRT-8	5	chr5.S_2582651	2,582,651	1.67E-06	*	*GRMZM2G110952*	China
	qRT-9	5	chr5.S_2582687	2,582,687	1.67E-06	*	*GRMZM2G110952*	China
	qRT-10	5	chr5.S_196508766	196,508,766	6.50E-08	*	*GRMZM2G022645*	China
	qRT-11	5	chr5.S_196511108	196,511,108	7.03E-07	*	*GRMZM2G022645*	China
	qRT-12	5	chr5.S_199862910	199,862,910	1.70E-08	*	*GRMZM2G021270*	China
	qRT-13	5	chr5.S_199871369	199,871,369	2.80E-12	*	*GRMZM2G317738*	China
	qRT-14	5	chr5.S_199984630	199,984,630	2.09E-11	*	*AC233960.1_FG002*	China

	qRT-15	5	PZE-105147361	200,115,761	7.65E-07	*	*GRMZM2G108996*	China	
	qRT-16	5	chr5.S_200292540	200,292,540	1.87E-07	*	*GRMZM2G034876*	China	
	qRT-17	5	chr5.S_200292661	200,292,661	2.49E-07	*	*GRMZM2G034876*	China	
	qRT-18	5	chr5.S_200293660	200,293,660	2.58E-08	*	*GRMZM2G034876*	China	
	qRT-19	5	chr5.S_200293667	200,293,667	3.64E-08	*	*GRMZM2G034876*	China	
	qRT-20	5	chr5.S_200293668	200,293,668	2.67E-08	*	*GRMZM2G034876*	China	
	qRT-21	5	chr5.S_200293669	200,293,669	2.67E-08	*	*GRMZM2G034876*	China	
	qRT-22	5	chr5.S_200293670	200,293,670	2.67E-08	*	*GRMZM2G034876*	China	
	qRT-23	5	chr5.S_200293693	200,293,693	7.60E-09	*	*GRMZM2G034876*	China	
	qRT-24	5	PZE-105147766	200,370,065	4.98E-10	*	*GRMZM2G035213*	China	
	qRT-25	5	chr5.S_200437162	200,437,162	6.90E-10	*	*GRMZM2G325019*	China	
	qRT-26	5	chr5.S_200438572	200,438,572	9.69E-10	*	*GRMZM2G325038*	China	
	qRT-27	5	chr5.S_200438920	200,438,920	3.89E-07	*	*GRMZM2G325038*	China	
	qRT-28	5	chr5.S_200481780	200,481,780	6.74E-08	*	*GRMZM2G092959*	China	
	qRT-29	5	chr5.S_200527129	200,527,129	7.51E-07	*	*GRMZM2G108087*	China	
	qRT-30	5	chr5.S_200527135	200,527,135	7.51E-07	*	*GRMZM2G108087*	China	
	qRT-31	5	chr5.S_200527246	200,527,246	6.41E-07	*	*GRMZM2G108087*	China	
	qRT-32	5	chr5.S_200528699	200,528,699	1.15E-07	*	*GRMZM2G108087*	China	
	qRT-33	5	chr5.S_200552153	200,552,153	2.64E-08	*	*GRMZM2G092749*	China	
	qRT-34	5	chr5.S_200552162	200,552,162	2.64E-08	*	*GRMZM2G092749*	China	
	qRT-35	5	chr5.S_200778988	200,778,988	1.26E-06	*	*GRMZM2G046909*	China	
	qRT-36	5	chr5.S_200779131	200,779,131	1.51E-06	*	*GRMZM2G046909*	China	
	qRT-37	5	SYN32527	202,132,225	1.40E-06	*	*GRMZM2G341957*	China	
	qRT-38	5	chr5.S_212093838	212,093,838	6.04E-07	*	*GRMZM2G039683*	China	
	qRT-39	6	chr6.S_7137764	7,137,764	8.17E-07	*	*GRMZM2G127299*	China	
	qRT-40	9	chr9.S_141452577	141,452,577	5.43E-07	*	*GRMZM5G818232*	China	
	qRT-41	10	chr10.S_4694476	4,694,476	2.62E-07	*	*GRMZM2G004534*	China	
	qRT-42	10	chr10.S_4695464	4,695,464	1.21E-07	*	*GRMZM2G004534*	China	
	qRT-43	10	chr10.S_4695681	4,695,681	3.70E-07	*	*GRMZM2G004534*	China	
	qRT-44	10	chr10.S_4695862	4,695,862	1.25E-06	*	*GRMZM2G004534*	China	
	qRT-45	10	chr10.S_4696252	4,696,252	1.75E-06	*	*GRMZM2G004534*	China	
	qRT-46	10	chr10.S_10600038	10,600,038	2.17E-07	*	*GRMZM2G402493*	China	
	qRT-47	10	chr10.S_10600039	10,600,039	2.17E-07	*	*GRMZM2G402493*	China	
	qRT-48	10	chr10.S_10600040	10,600,040	2.17E-07	*	*GRMZM2G402493*	China	
	qRT-49	10	chr10.S_136945275	136,945,275	1.76E-06	*	*AC235534.1_FG001*	China	
	qRT-50	10	chr10.S_140994710	140,994,710	6.19E-08	*	*GRMZM2G109753*	China	
	qRT-51	10	chr10.S_140994750	140,994,750	6.19E-08	*	*GRMZM2G109753*	China	
**AT**	*qAT-1*	1	chr1_88972694	88972694	2.06E-06	0.1542	GRMZM2G089291	China	(Xiao et al., 2020)
	qAT-2	2	chr2_99655269	99655269	1.98E-06	0.1602	AC196212.3_FG001	China	
	qAT-3	5	chr5_199221258	199221258	1.74E-07	0.2062	GRMZM2G141975	China	
	qAT-4	5	chr5_199340929	199340929	3.71E-08	0.2623	GRMZM2G060834	China	
	qAT-5	5	chr5_199422724	199422724	7.76E-08	0.2447	GRMZM2G376067	China	
	qAT-6	5	chr5_200376352	200376352	8.33E-10	0.3215	GRMZM2G035213	China	
	qAT-7	6	chr6_24552456	24552456	3.42E-06	0.1521	GRMZM2G111579	China	
	qAT-8	6	chr6_51279284	51279284	7.99E-07	0.1588	GRMZM2G000707	China	
	qAT-9	6	chr6_119248607	119248607	1.61E-06	0.1505	GRMZM2G104511	China	
**BT**	*qBT-1*	1	chr1_21361394	21361394	4.64E-08	0.2348	GRMZM5G896834	China	
	qBT-2	1	chr1_27801286	27801286	3.16E-06	0.1704	GRMZM2G040121	China	
	qBT-3	1	chr1_58731650	58731650	1.88E-06	0.1517	GRMZM2G097843	China	
	qBT-4	1	chr1_83332454	83332454	3.85E-07	0.1951	GRMZM2G099467	China	
	qBT-5	1	chr1_88972694	88972694	1.88E-08	0.2117	GRMZM2G089291	China	

	qBT-6	1	chr1_96013126	96013126	2.23E-06	0.1754	GRMZM2G042538	China
	qBT-7	2	chr2_137684715	137684715	4.53E-06	0.1544	No	China
	qBT-8	3	chr3_131370095	131370095	6.56E-07	0.1685	GRMZM2G447657	China
	qBT-9	3	chr3_204172506	204172506	5.66E-06	0.2048	GRMZM2G330298	China
	qBT-10	3	chr3_228566338	228566338	4.51E-06	0.1418	AC185276.2_FG006	China
	qBT-11	4	chr4_7038838	7038838	9.11E-08	0.2292	GRMZM2G702335	China
	qBT-12	5	chr5_3322754	3322754	4.01E-08	0.2247	AC210013.4_FG005	China
	qBT-13	5	chr5_66308848	66308848	5.76E-08	0.1949	GRMZM2G006282	China
	qBT-14	5	chr5_193510153	193510153	1.12E-06	0.2219	GRMZM2G362848	China
	qBT-15	5	chr5_199221258	199221258	5.03E-10	0.2834	GRMZM2G141975	China
	qBT-16	5	chr5_199340929	199340929	5.11E-08	0.2103	GRMZM2G060834	China
	qBT-17	6	chr6_9685268	9685268	1.49E-06	0.1664	GRMZM2G055238	China
	qBT-18	6	chr6_20381982	20381982	4.78E-06	0.1521	No	China
	qBT-19	6	chr6_24552456	24552456	1.36E-06	0.1594	GRMZM2G111579	China
	qBT-20	6	chr6_67456686	67456686	4.55E-06	0.1424	GRMZM2G137440	China
	qBT-21	6	chr6_72442017	72442017	6.07E-06	0.1384	AC194478.3_FG005	China
	qBT-22	6	chr6_73214934	73214934	3.82E-06	0.1414	No	China
	qBT-23	6	chr6_82489783	82489783	4.85E-06	0.1428	GRMZM2G150348	China
	qBT-24	6	chr6_113453200	113453200	1.76E-07	0.2042	GRMZM2G020742	China
	qBT-25	6	chr6_117903333	117903333	4.56E-06	0.1452	GRMZM2G435357	China
	qBT-26	6	chr6_119248607	119248607	6.38E-06	0.1358	GRMZM2G104511	China
	qBT-27	7	chr7_105008316	105008316	5.42E-06	0.1371	GRMZM2G150832	China
	qBT-28	7	chr7_161885914	161885914	1.45E-06	0.1628	GRMZM2G411916	China
	qBT-29	9	chr9_4311642	4311642	5.03E-06	0.139	GRMZM2G465046	China
	qBT-30	9	chr9_94675466	94675466	1.16E-07	0.2044	No	China
	qBT-31	9	chr9_123234213	123234213	5.63E-06	0.1424	GRMZM2G057865	China
	qBT-32	9	chr9_126210698	126210698	4.53E-07	0.177	AC207309.3_FG010	China
**DT**	*qDT-1*	4	chr4_169577228	169577228	1.41E-06	0.1626	GRMZM2G123818	China
**RT**	*qRT-1*	1	chr1_80813652	80813652	6.42E-06	0.1321	GRMZM2G049852	China
	qRT-2	1	chr1_88972694	88972694	1.89E-07	0.1809	GRMZM2G089291	China
	qRT-3	2	chr2_101807859	101807859	5.75E-06	0.1385	GRMZM5G878497	China
	qRT-4	5	chr5_199221258	199221258	2.64E-07	0.1913	GRMZM2G141975	China
	qRT-5	5	chr5_199422724	199422724	8.82E-08	0.2346	GRMZM2G376067	China
	qRT-6	5	chr5_200376352	200376352	3.00E-10	0.3098	GRMZM2G035213	China
	qRT-7	9	chr9_14027435	14027435	1.87E-07	0.1999	GRMZM2G149126	China
	qRT-8	9	chr9_50743440	50743440	6.35E-06	0.1573	GRMZM2G408256	China
	qRT-9	9	chr9_58232718	58232718	1.45E-06	0.1759	GRMZM2G076272	China
	qRT-10	9	chr9_59379168	59379168	4.86E-06	0.1367	GRMZM2G005664	China
	qRT-11	9	chr9_60230402	60230402	3.74E-06	0.1553	GRMZM2G104481	China
	qRT-12	9	chr9_92205956	92205956	6.65E-06	0.1439	GRMZM2G076526	China
	qRT-13	9	chr9_98638085	98638085	4.54E-07	0.1625	GRMZM2G177659	China
**TT**	*qTT-1*	2	chr2_99655269	99655269	3.24E-06	0.1587	AC196212.3_FG001	China
	qTT-2	5	chr5_199340929	199340929	2.95E-07	0.2133	GRMZM2G060834	China
	qTT-3	6	chr6_24552456	24552456	7.25E-07	0.1768	GRMZM2G111579	China
	qTT-4	6	chr6_51279284	51279284	4.41E-06	0.1421	GRMZM2G000707	China
**AT3**	*qAT3-1*	4	chr4_179282037	179282037	2.70E-06	0.1472	GRMZM5G848268	China
	qAT3-2	5	chr5_199422724	199422724	4.11E-07	0.228	GRMZM2G376067	China
	qAT3-3	5	chr5_200376352	200376352	5.03E-10	0.3032	GRMZM2G035213	China
**RT3**	*qRT3-1*	1	chr1_15966886	15966886	5.33E-06	0.1877	GRMZM2G156486	China
	qRT3-2	1	chr1_29073536	29073536	2.77E-06	0.1548	GRMZM2G447504	China
	qRT3-3	1	chr1_86530409	86530409	3.78E-06	0.1675	GRMZM2G017011	China
	qRT3-4	1	chr1_89170681	89170681	9.22E-08	0.2201	AC204552.3_FG001	China

qRT3-5	1	chr1_118638363	118638363	4.26E-06	0.1405	No	China
qRT3-6	1	chr1_124668839	124668839	5.36E-06	0.145	GRMZM2G359822	China
qRT3-7	1	chr1_132598690	132598690	6.62E-07	0.1642	GRMZM2G170839	China
qRT3-8	1	chr1_151897716	151897716	3.39E-06	0.1523	No	China
qRT3-9	1	chr1_209701182	209701182	2.10E-06	0.149	GRMZM5G825854	China
qRT3-10	1	chr1_211870007	211870007	1.20E-06	0.1591	GRMZM2G043819	China
qRT3-11	1	chr1_214620377	214620377	1.51E-07	0.1923	GRMZM2G536825	China
qRT3-12	1	chr1_269769913	269769913	5.46E-06	0.1551	GRMZM2G077147	China
qRT3-13	1	chr1_294952643	294952643	3.49E-06	0.1432	GRMZM2G123876	China
qRT3-14	2	chr2_1320971	1320971	2.36E-06	0.1581	GRMZM2G147854	China
qRT3-15	2	chr2_58478180	58478180	1.35E-06	0.1564	GRMZM2G062504	China
qRT3-16	2	chr2_143586615	143586615	5.67E-06	0.1496	GRMZM2G168829	China
qRT3-17	2	chr2_147917250	147917250	4.05E-07	0.1711	GRMZM2G424491	China
qRT3-18	2	chr2_179437626	179437626	4.23E-06	0.1407	GRMZM2G703265	China
qRT3-19	2	chr2_213974400	213974400	3.87E-06	0.146	GRMZM2G354013	China
qRT3-20	2	chr2_222865364	222865364	2.69E-06	0.1695	GRMZM2G154716	China
qRT3-21	2	chr2_229961991	229961991	1.58E-06	0.154	GRMZM2G333448	China
qRT3-22	3	chr3_54193516	54193516	5.28E-06	0.1451	GRMZM5G830319	China
qRT3-23	3	chr3_57167369	57167369	5.12E-06	0.1436	GRMZM2G016754	China
qRT3-24	3	chr3_81038099	81038099	1.87E-06	0.1504	GRMZM2G156033	China
qRT3-25	3	chr3_101087707	101087707	3.04E-06	0.1467	GRMZM2G401179	China
qRT3-26	3	chr3_113948740	113948740	6.40E-06	0.1504	AC230011.2_FG002	China
qRT3-27	3	chr3_197797519	197797519	3.92E-06	0.1414	GRMZM2G043212	China
qRT3-28	3	chr3_198303954	198303954	2.89E-06	0.1478	GRMZM2G141299	China
qRT3-29	3	chr3_210354275	210354275	7.81E-07	0.1802	GRMZM2G437460	China
qRT3-30	3	chr3_224713856	224713856	4.18E-06	0.1413	GRMZM2G034684	China
qRT3-31	4	chr4_2969678	2969678	2.91E-06	0.1758	GRMZM2G469483	China
qRT3-32	4	chr4_11223788	11223788	3.32E-07	0.2017	GRMZM2G068330	China
qRT3-33	4	chr4_23192577	23192577	6.39E-06	0.153	GRMZM2G389937	China
qRT3-34	4	chr4_31532503	31532503	2.01E-07	0.2022	GRMZM2G052670	China
qRT3-35	4	chr4_145549902	145549902	1.91E-06	0.1538	AC205672.3_FG001	China
qRT3-36	4	chr4_177073590	177073590	5.09E-06	0.1523	GRMZM2G006297	China
qRT3-37	4	chr4_228578491	228578491	2.91E-06	0.1475	GRMZM2G044848	China
qRT3-38	5	chr5_92429962	92429962	4.56E-06	0.1405	GRMZM2G179454	China
qRT3-39	5	chr5_200376352	200376352	3.72E-10	0.2835	GRMZM2G035213	China
qRT3-40	6	chr6_34160278	34160278	2.28E-06	0.1668	GRMZM2G147804	China
qRT3-41	6	chr6_80802049	80802049	2.50E-06	0.1577	GRMZM2G017421	China
qRT3-42	7	chr7_54479667	54479667	4.30E-06	0.145	GRMZM2G032766	China
qRT3-43	7	chr7_107374028	107374028	5.19E-06	0.1526	No	China
qRT3-44	8	chr8_60106765	60106765	4.34E-06	0.1419	GRMZM2G088291	China
qRT3-45	8	chr8_88681491	88681491	6.38E-06	0.1474	GRMZM2G044531	China
qRT3-46	8	chr8_99084878	99084878	1.66E-06	0.1717	GRMZM2G046824	China
qRT3-47	8	chr8_125477938	125477938	4.08E-06	0.1407	GRMZM5G855411	China
qRT3-48	9	chr9_3901902	3901902	3.89E-06	0.1412	GRMZM2G048243	China
qRT3-49	9	chr9_11330574	11330574	2.70E-06	0.1624	GRMZM2G404855	China
qRT3-50	9	chr9_84233825	84233825	1.36E-06	0.1544	GRMZM2G092256	China
qRT3-51	9	chr9_94815283	94815283	2.59E-06	0.1838	GRMZM5G805627	China
qRT3-52	9	chr9_96620660	96620660	2.30E-06	0.1518	GRMZM2G162623	China
qRT3-53	9	chr9_100954354	100954354	3.76E-06	0.1451	AC226445.2_FG002	China
qRT3-54	9	chr9_103569663	103569663	3.16E-06	0.1466	GRMZM2G010863	China
qRT3-55	9	chr9_138495501	138495501	5.68E-06	0.1592	GRMZM2G145451	China
qRT3-56	10	chr10_47855681	47855681	5.27E-08	0.2002	GRMZM2G570790	China

**TT3**	*qTT3-1*	7	chr7_15499854	15499854	2.68E-06	0.1486	GRMZM2G179090	China	
**ATLOG**	*qATLOG-1*	4	rs131181045	36451477	[-$\log_{10}$=4.5]	10.14	*	Portugal	(Alves et al., 2020)
	qATLOG-2	4	rs129686170	44985143	[-$\log_{10}$=4.2]	10.00	*	Portugal	
	qATLOG-3	4	rs129686172	44985307	[-$\log_{10}$=4.2]	10.00	*	Portugal	
	qATLOG-4	4	rs129686173	44985374	[-$\log_{10}$=4.2]	10.00	*	Portugal	
	qATLOG-5	4	rs129686175	44985417	[-$\log_{10}$=4.2]	10.00	*	Portugal	
	qATLOG-6	4	rs129686679	45151080	[-$\log_{10}$=4.2]	10.00	*	Portugal	
	qATLOG-7	4	rs131452300	45215363	[-$\log_{10}$=4.2]	10.00	*	Portugal	
	qATLOG-8	4	rs129686902	45289528	[-$\log_{10}$=4.2]	10.00	*	Portugal	
	qATLOG-9	4	rs131452316	45289828	[-$\log_{10}$=4.2]	10.00	*	Portugal	
	qATLOG-10	5	rs130180529	200420347	[-$\log_{10}$=5.6]	13.31	*	Portugal	
	qATLOG-11	5	rs130180536	200421939	[-$\log_{10}$=5.6]	13.31	*	Portugal	
	qATLOG-12	5	rs130200955_1	212731495	[-$\log_{10}$=4.6]	10.20	*	Portugal	
	qATLOG-13	5	rs130200955_2	212731495	[-$\log_{10}$=4.6]	10.20	*	Portugal	
	qATLOG-14	10	rs128647552	149506640	[-$\log_{10}$=4.3]	9.58	*	Portugal	
**DTLOG**	*qDTLOG-1*	1	rs128990610	298814922	[-$\log_{10}$=5.9]	19.67	*	Portugal	
	qDTLOG-2	1	rs128990610	298814922	[-$\log_{10}$=4.6]	13.37	*	Portugal	
	qDTLOG-3	1	rs128990613	298815104	[-$\log_{10}$=5.9]	19.67	*	Portugal	
	qDTLOG-4	1	rs128990613	298815104	[-$\log_{10}$=4.6]	13.37	*	Portugal	
	qDTLOG-5	2	rs131335950	41312594	[-$\log_{10}$=4.4]	12.81	*	Portugal	
	qDTLOG-6	2	rs129211723	204555793	[-$\log_{10}$=4.1]	12.14	*	Portugal	
	qDTLOG-7	3	rs131178368	17449753	[-$\log_{10}$=5.7]	13.75	*	Portugal	
	qDTLOG-8	3	rs131178368	17449753	[-$\log_{10}$=4.1]	12.21	*	Portugal	
	qDTLOG-9	3	rs129286980	170499908	[-$\log_{10}$=5.4]	12.60	*	Portugal	
	qDTLOG-10	3	rs129286980	170499908	[-$\log_{10}$=4.6]	12.87	*	Portugal	
	qDTLOG-11	4	rs129619500	2985173	[-$\log_{10}$=4.0]	13.48	*	Portugal	
	qDTLOG-12	4	rs131462576	71242320	[-$\log_{10}$=4.0]	10.26	*	Portugal	
	qDTLOG-13	5	rs131510695	33437545	[-$\log_{10}$=4.0]	11.98	*	Portugal	
	qDTLOG-14	6	rs131553173	22002210	[-$\log_{10}$=4.2]	11.44	*	Portugal	
	qDTLOG-15	6	rs131568062	59267250	[-$\log_{10}$=4.6]	14.10	*	Portugal	
	qDTLOG-16	6	rs131568326	60040082	[-$\log_{10}$=5.2]	16.20	*	Portugal	
	qDTLOG-17	6	rs131568350	60174784	[-$\log_{10}$=5.3]	18.31	*	Portugal	
	qDTLOG-18	7	rs131179571	135606083	[-$\log_{10}$=4.2]	15.45	*	Portugal	
	qDTLOG-19	7	rs131181519	157314622	[-$\log_{10}$=4.1]	13.09	*	Portugal	
**GT**	*qGT-1*	1	rs131269931	82014738	[-$\log_{10}$=4.3]	9.50	*	Portugal	
	qGT-2	1	rs128653362	82031110	[-$\log_{10}$=4.0]	8.54	*	Portugal	
	qGT-3	1	rs131280450	101025418	[-$\log_{10}$=4.1]	7.22	*	Portugal	
	qGT-4	4	rs129940387	225448671	[-$\log_{10}$=4.2]	7.80	*	Portugal	
	qGT-5	8	rs131179677	26077645	[-$\log_{10}$=4.8]	9.27	*	Portugal	
**δT**	*qδT*	1	Zm00001d028680	2,788,600-42,791,622	2.625E-06	*	Ankyrin repeat-containing protein	Aurora, NY	Hershberger et al. (2022)
**γT**	*qγT*	1	Zm00001d028680	42,788,600-42,791,622	5.774E-06	*	Ankyrin repeat-containing protein	Aurora, NY	
**ΣT**	*qΣT*	1	Zm00001d028680	42,788,600-42,791,622	1.968E-05	*	Ankyrin repeat-containing protein	Aurora, NY	
**γT**	*qγT*	1	Zm00001d034433	292,905,093-292,912,832	1.804E-05	*	Heat stress transcription factor 11	Aurora, NY	
**ΣT**	*qΣT*	1	Zm00001d034433	292,905,093-292,912,832	2.045E-05	*	Heat stress transcription factor 11	Aurora, NY	
**ΣT**	*qΣT*	2	Zm00001d007121	222,781,921-222,806,658	2.388E-07	*	CW-type Zinc Finger	Aurora, NY	
**γT**	*qγT*	2	Zm00001d007121	222,781,921-222,806,658	7.702E-07	*	CW-type Zinc Finger	Aurora, NY	

**ΣT3**	*qΣT3*	2	Zm00001d007489	232,843,576-232,847,278	8.183E-07	*	Zinc-finger domain of monoamine-oxidase A repressor R1	Aurora, NY
**ΣT**	*qΣT*	3	Zm00001d040089	27,065,943-27,068,338	1.975E-05	*	Nodulin MtN21 /EamA-like transporter family protein	Aurora, NY
**ΣT3**	*qΣT3*	3	Zm00001d042231	156,958,516-156,959,137	2.572E-06	*	Basal endosperm transfer layer10	Aurora, NY
**ΣT3**	*qΣT3*	3	Zm00001d043479	201,465,990-201,469,783	6.036E-06	*	Coiled-coil domain-containing protein 25	Aurora, NY
**αT**	*qαT*	4	Zm00001d049809	45,664,408-45,668,447	3.294E-06	*	*	Aurora, NY
**γT**	*qγT*	4	Zm00001d053899	243,343,911-243,347,704	1.066E-05	*	Rho-related protein from plants2	Aurora, NY
**αT/γT**	*q(αT/γT)*	5	Zm00001d017746	205,825,586-205,829,216	1.133E-15	*	Vitamin E synthesis4	Aurora, NY
**γT/(γT+αT)**	*qγT/(γT+αT)*	5	Zm00001d017746	205,825,586-205,829,216	3.105E-15	*	Vitamin E synthesis4	Aurora, NY
**αT**	*qαT*	5	Zm00001d017746	205,825,586-205,829,216	3.100E-11	*	Vitamin E synthesis4	Aurora, NY
**δT/αT**	*q(δT/αT)*	5	Zm00001d017746	205,825,586-205,829,216	3.030E-08	*	Vitamin E synthesis4	Aurora, NY
**γT**	*qγT*	5	Zm00001d017746	205,825,586-205,829,216	3.847E-08	*	Vitamin E synthesis4	Aurora, NY
**γT**	*qγT*	5	Zm00001d018086	213,976,676-213,977,392	1.329E-05	*	*	Aurora, NY
**ΣT**	*qΣT*	6	Zm00001d036727	99,181,767-99,186,018	2.166E-05	*	Alpha/beta-Hydrolases superfamily protein	Aurora, NY
**ΣT**	*qΣT*	6	Zm00001d038608	160,896,290-160,898,350	9.560E-06	*	Class VI heat shock protein	Aurora, NY
**ΣT3+ΣT**	*q(ΣT3+ΣT)*	6	Zm00001d039066	169,497,057-169,501,065	4.789E-07	*	Galactose mutarotase-like superfamily protein	Aurora, NY
**ΣT**	*qΣT*	4	Zm00001d011365	148,696,781-148,699,784	1.03E-05	*	Alpha/beta-Hydrolases superfamily protein	Aurora, NY
**ΣT**	*qΣT*	5	Zm00001d011825	162,158,586-162,160,692	1.71E-05	*	Probable gamma-secretase subunit PEN-2	Aurora, NY
**ΣT**	*qΣT*	2	Zm00001d011923	164,678,915-164,684,859	3.01E-06	*	TNF receptor-associated factor 26	Aurora, NY
**γT**	*qγT*	4	Zm00001d011923	164,678,915-164,684,859	1.01E-05	*	TNF receptor-associated factor 26	Aurora, NY

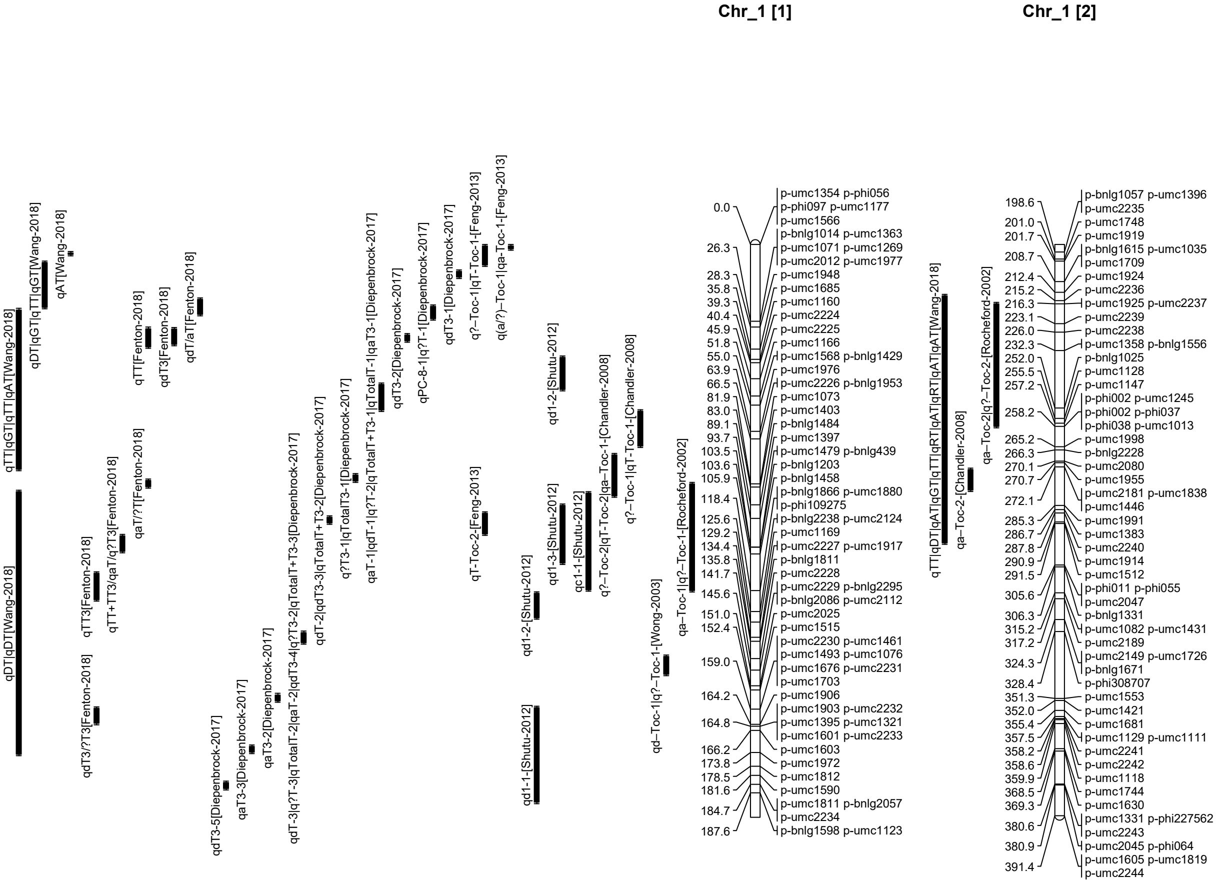

Figure S1. Chromosome locations of QTL that control seed α–tocopherol (α–Toc), γ–tocopherol (γ–Toc), δ–tocopherol (δ–Toc), total tocopherol (T–Toc) contents and (α/γ)–Toc ratio in maize from 2002 to 2022.

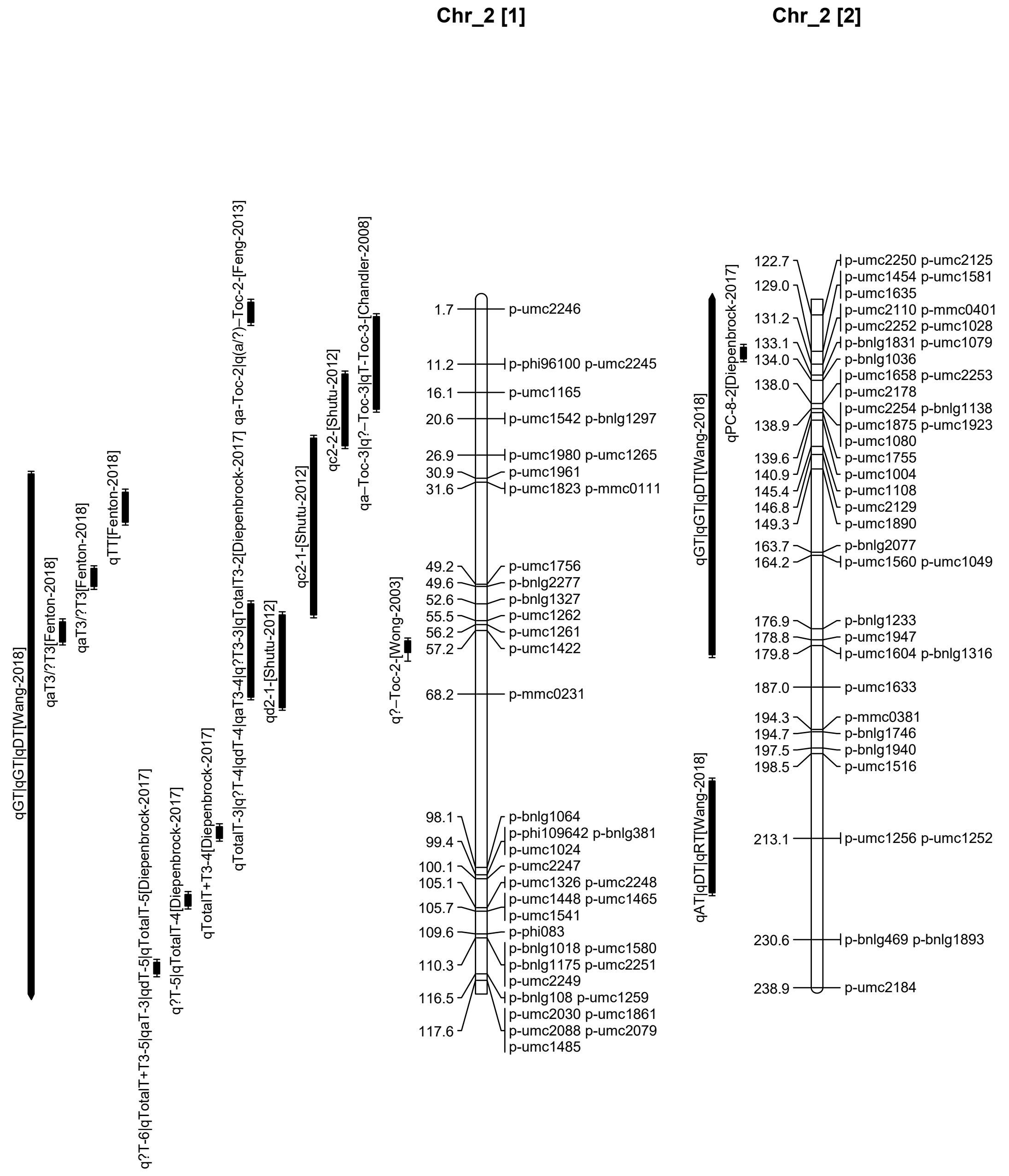

Figure S1. Continued.

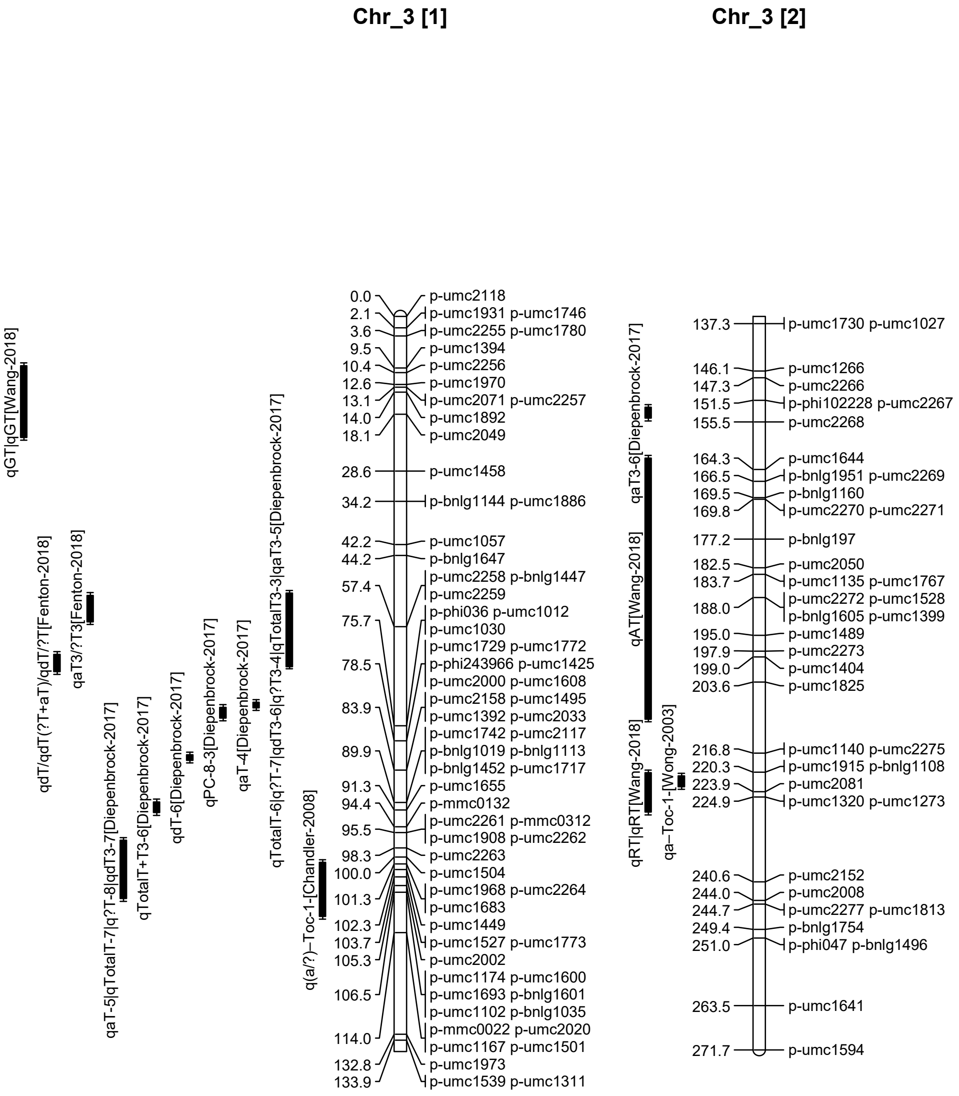

Figure S1. Continued.

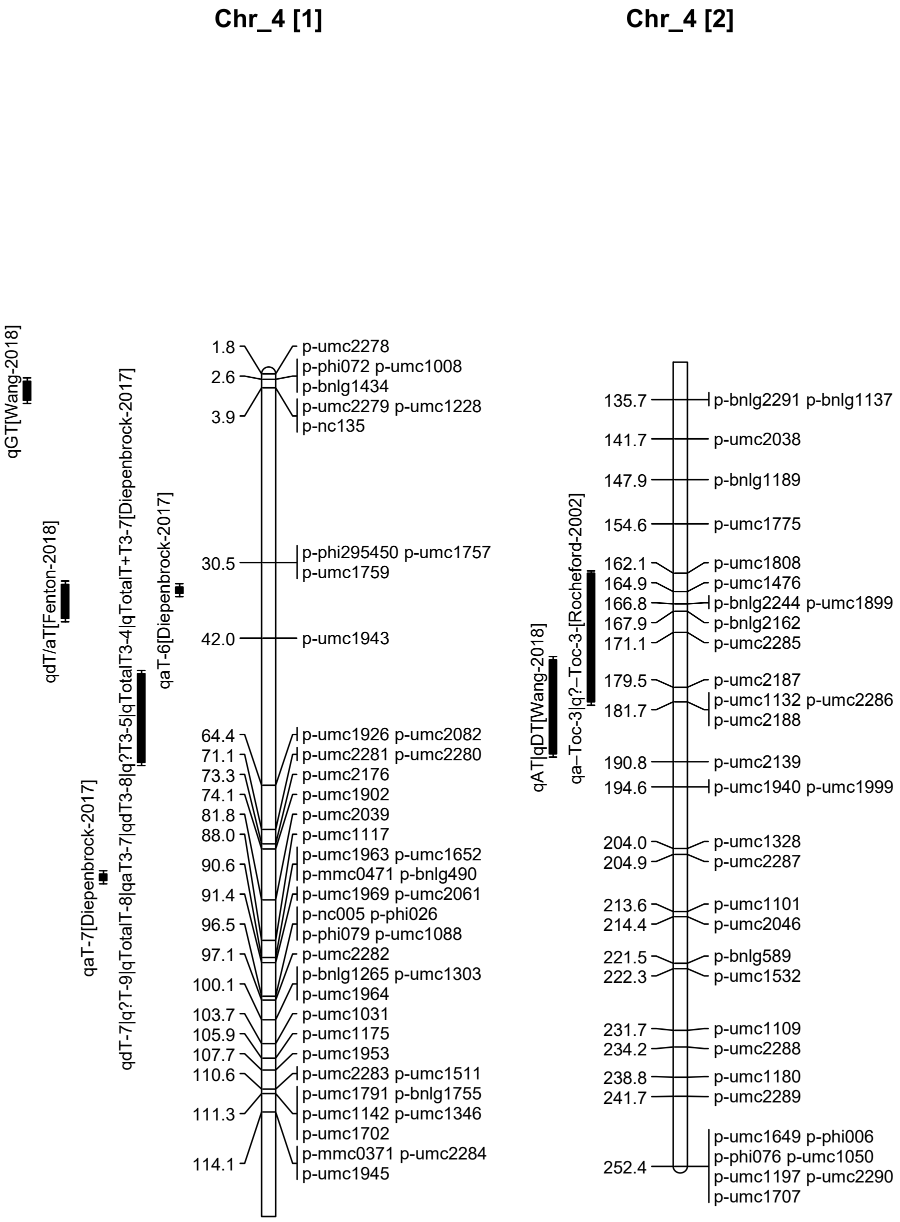

Figure S1. Continued.

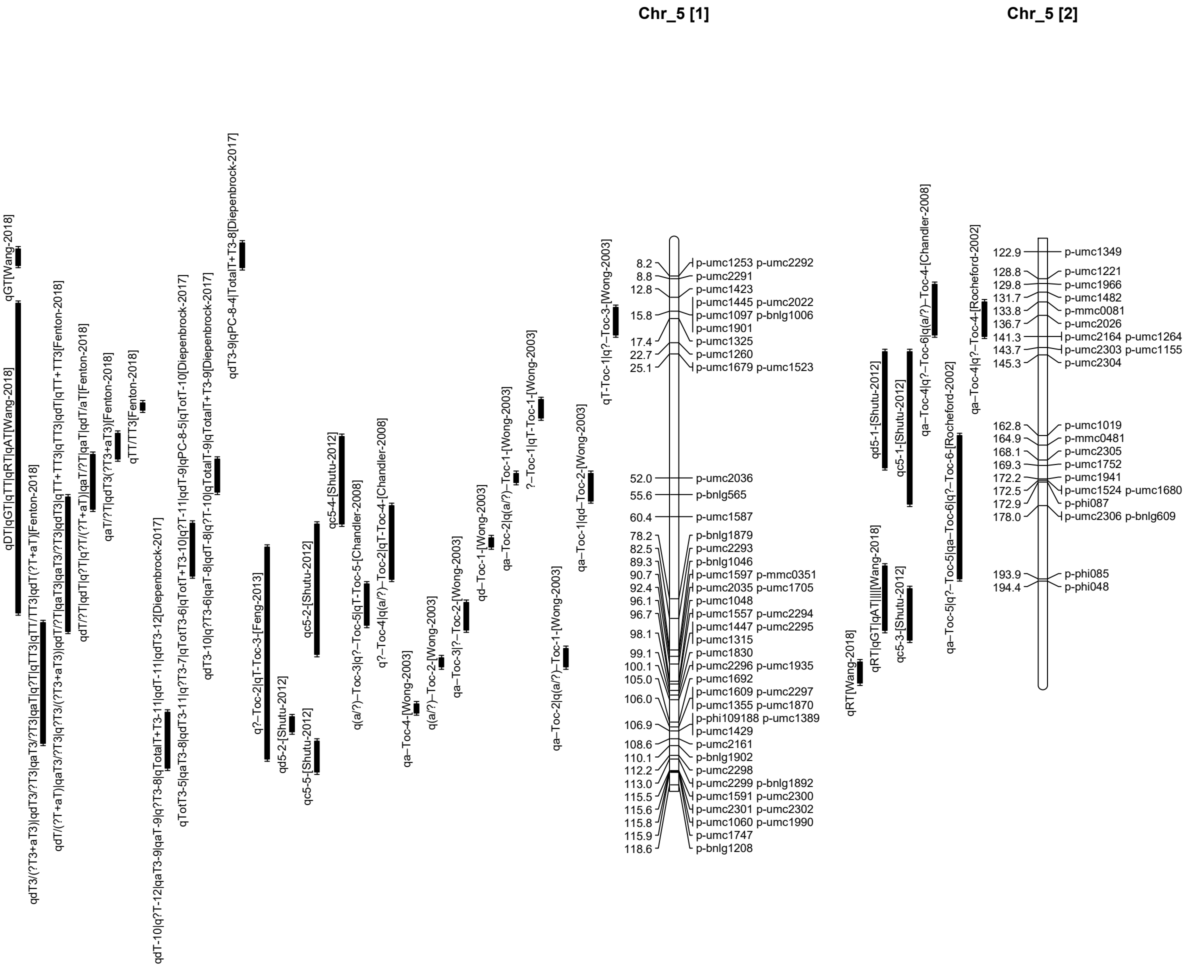

Figure S1. Continued.

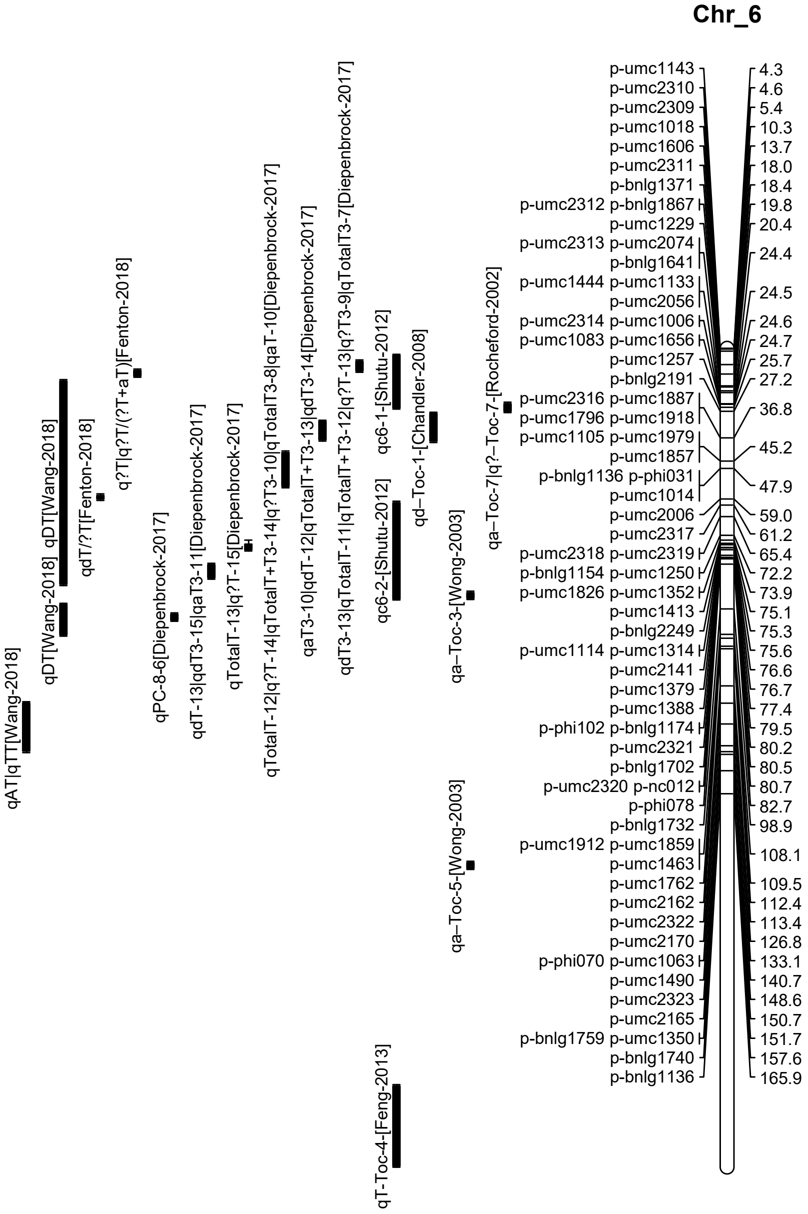

Figure S1. Continued.

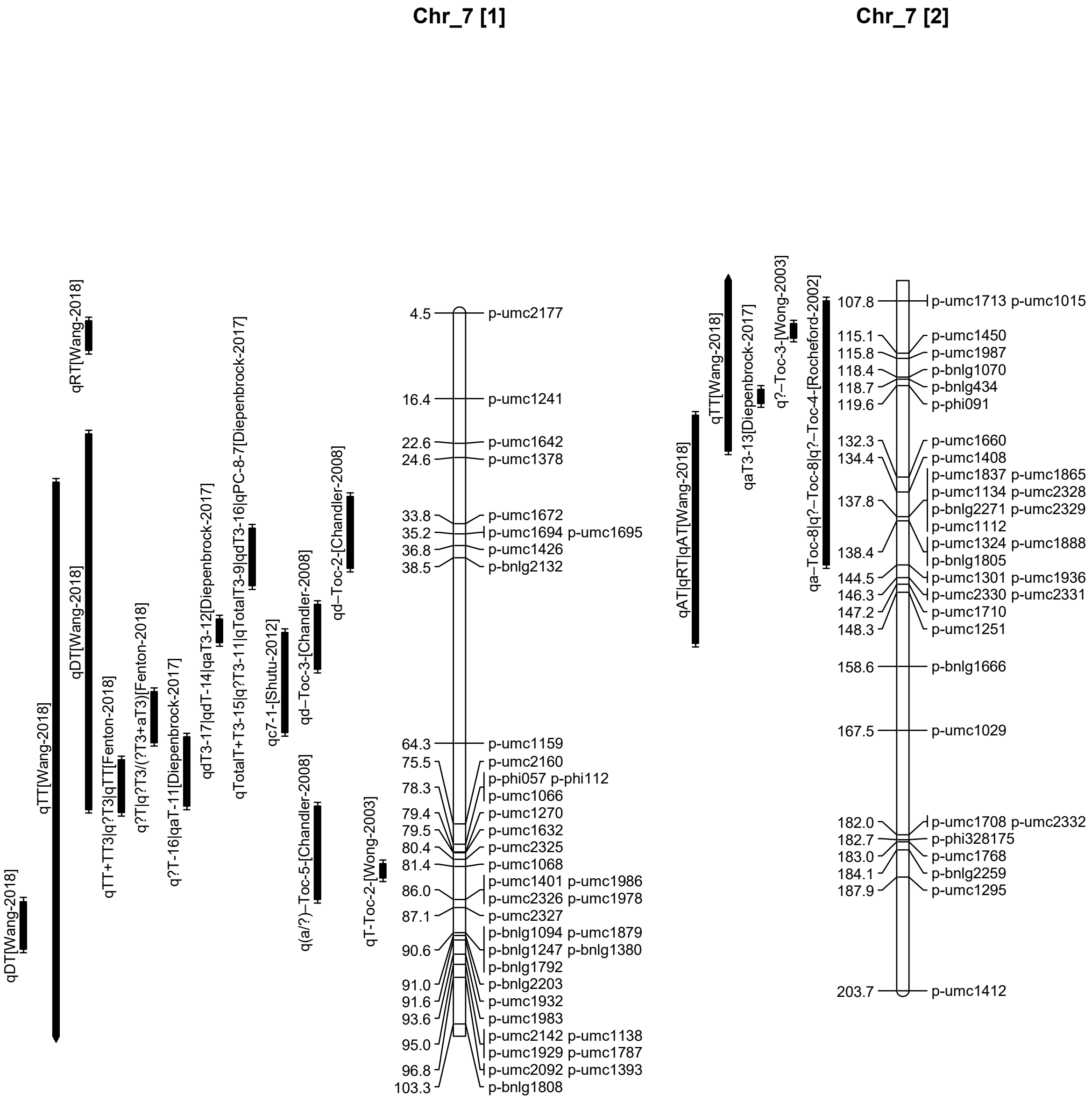

Figure S1. Continued.

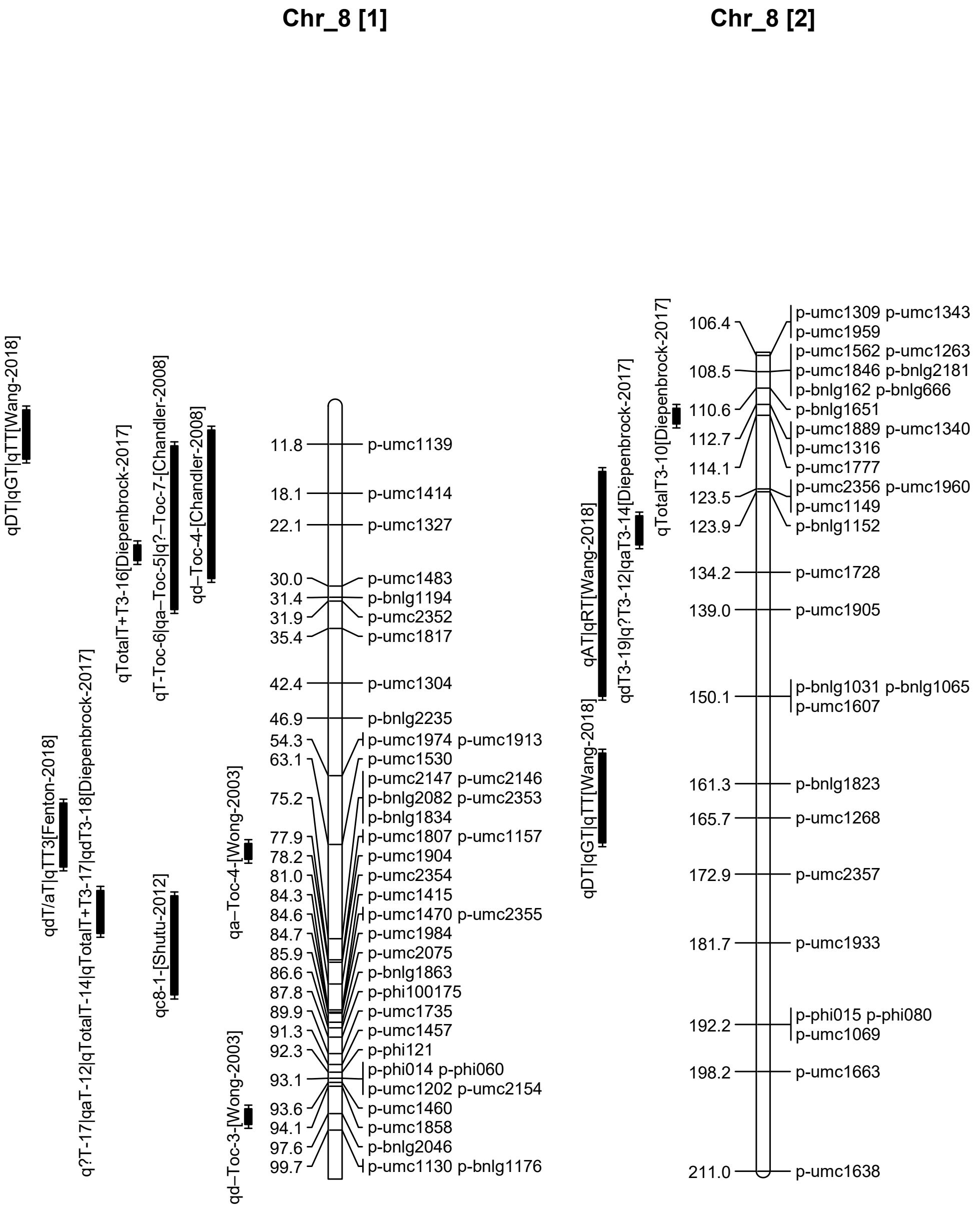

Figure S1. Continued.

Chr_9

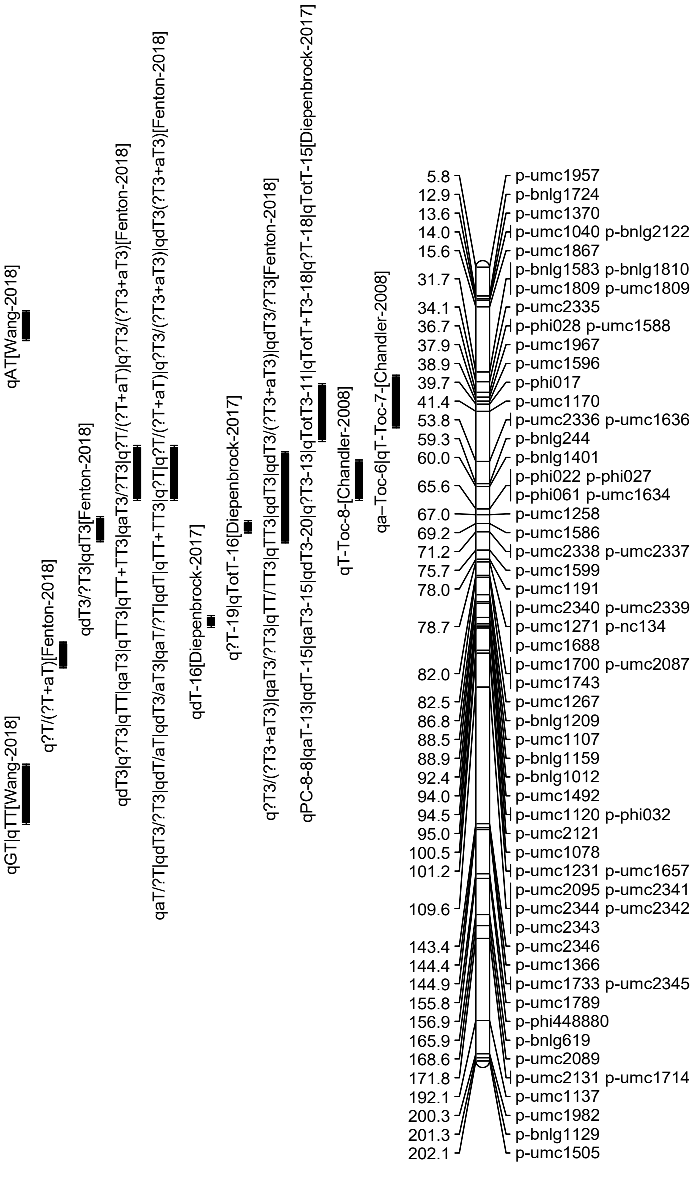

Figure S1. Continued.

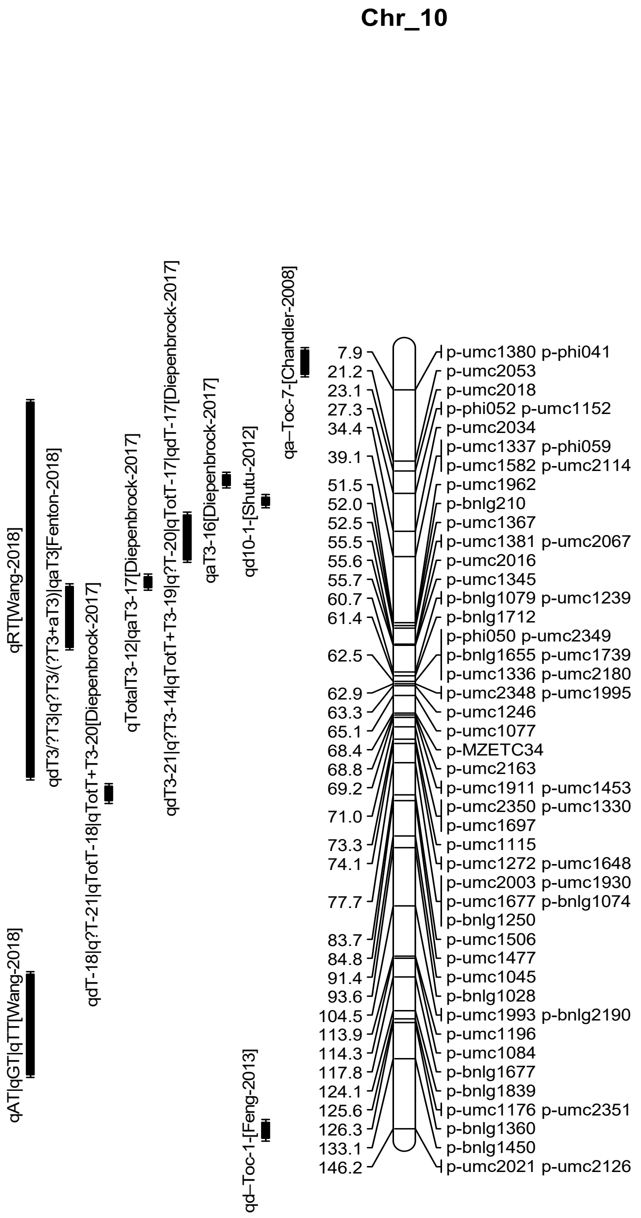

Figure S1. Continued.
